# Design Principles of Responsive Relaxometric ^19^F Contrast Agents: Evaluation from the Point of View of Relaxation Theory and Experimental Data

**DOI:** 10.1021/acs.inorgchem.2c03451

**Published:** 2022-11-16

**Authors:** Mariusz Zalewski, Dawid Janasik, Adrianna Wierzbicka, Tomasz Krawczyk

**Affiliations:** Department of Chemical Organic Technology and Petrochemistry, Faculty of Chemistry, Silesian University of Technology, Krzywoustego 4, 44-100Gliwice, Poland

## Abstract

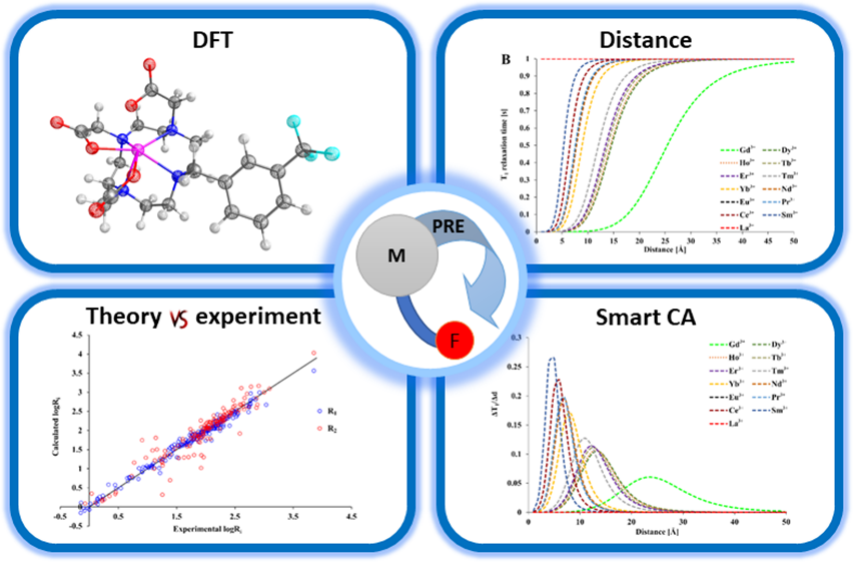

^19^F magnetic resonance imaging (MRI) is a promising tool in medical diagnostics. An important class of ^19^F MRI contrast agents is based on paramagnetic resonance enhancement. This effect allows an improvement in sensitivity by increasing the number of scans per unit of time or facilitates the development of responsive contrast agents that are based on changes in relaxation rates as a detection principle. In this work, Bloch–Redfield–Wangsness relaxation theory was used to predict the relaxation properties of existing lanthanoid and transition metal complexes of fluoroorganic ligands and to evaluate several design strategies for responsive contrast agents. Electron–nucleus dipole–dipole, Curie relaxation, and contact interactions were included in the model. Potential significance of chemical shift anisotropy–anisotropic dipolar shielding cross-correlation was discussed. The calculated and experimental results were well aligned. The presented model, along with the optimized field-dependent values of electronic relaxation times, could be used for the preliminary selection of the optimal metal ion for applications in ^19^F MRI. The results indicate potential advantages of other metal ions in addition to Gd^3+^ particularly Cu^2+^, Mn^2+^, Ni^2+^, Fe^3+^, and other lanthanoids as a part of ^19^F contrast agents.

## Abstract 2

The relationship between the structure of fluoroorganic paramagnetic complexes and their relaxation properties was investigated from the lens of relaxation theory. The field dependence of the electronic relaxation times was determined for selected lanthanoids and transition metals based on ^19^F relaxation data. Design principles and limitations of various types of ^19^F contrast agents were discussed. The usefulness of ions other than Gd^3+^ in the development of novel ^19^F MRI contrast agents was presented. Theoretical prediction of transverse and longitudinal ^19^F relaxation rates of a variety of fluoroorganic paramagnetic complexes was attempted based on Bloch−Redfield−Wangsness relaxation theory. The main factors that affect the results and the contribution of different relaxation mechanisms were discussed. Based on the obtained data, a suitable library of paramagnetic ions for potential use in the design of ^19^F contrast agents was presented.

## Introduction

The physical foundations of nuclear magnetic resonance (NMR) were established at the turn of the 1950s and 1960s. Since then, this technique has found widespread use in physics, chemistry, and biochemistry, especially for structural studies. In the 1970s, the use of MR as a safe, non-invasive imaging technique in medicine gained traction, and it is one of the leading techniques for the diagnosis of pathological changes in soft tissues. ^1^H magnetic resonance imaging (MRI) uses subtle differences in the relaxation of water protons in different tissues. To obtain more reliable results, allowing for unambiguous differentiation between normal organs and pathological changes, paramagnetic contrast agents are frequently used to affect the relaxation of protons present in their vicinity.^1^ Gd^3+^, Mn^2+^, and Fe^3+^ are commonly used as paramagnetic ions, with gadolinium complexes being the largest group of commercial contrast agents. The potential accumulation of gadolinium ions in the body can lead, in some cases, to dangerous complications, such as nephrogenic systemic fibrosis.^2^ An alternative way to improve the diagnostic value of MR images is the use of heteronuclear resonances such as ^3^He, ^13^C, ^23^Na, ^31^P, ^129^Xe, and foremost ^19^F.^3,4^ The undoubted advantages of using ^19^F are its 100% natural abundance and similar sensitivity to ^1^H (83%).^5^ Furthermore, it is possible to use existing clinical MR scanners for the ^1^H and ^19^F modalities after slight hardware modifications (i.e., tuning at 282 MHz for ^19^F instead of 300 MHz for ^1^H).^6−8^ Because fluorine is not present in soft tissues, ^19^F MRI is essentially a background-free technique^9^ since only fluorine atoms introduced with the contrast agent are visible. Typically, the ^19^F MR image is superimposed with the ^1^H anatomical images for precise localization of the injected fluorinated tracer.^10,11^

An interesting class of contrast agents is responsive agents, sometimes referred to as “smart” agents, which are activated under the influence of a specific trigger in the environment. These triggers may be an enzyme,^12^ pH gradient,^13^ metal ions,^14^ or a change in the oxygen concentration.^15^ Due to many potential applications in medical diagnostics, the field of responsive contrast agents is a growing area of research.^9^ The detection principle generally relies on a chemical shift^16−18^ or changes in relaxation times due to paramagnetic relaxation enhancement (PRE)^19,20^ exerted by paramagnetic complexes. Relaxation rates can be affected by other stimuli such as self-assembly,^21^ disassembly,^12,22^ or conformational changes^23^ that lead to mobility changes manifested in *T*_2_-weighted images. Such transformation may be irreversible or reversible. Because the ^19^F NMR signal is concentration-dependent, a means of calibration is required for practical applications. The PRE phenomenon is also important in the design of simple ^19^F contrast agents consisting of paramagnetic metal ion complexes with pendant fluoroorganic moieties. Similarly, the design of ^19^F contrast agents enables the tuning of relaxation times to maximize the number of scans per unit time and, consequently, improve the S/N ratio. There are several ions with constant magnetic moments which could be useful as PRE agents. These ions have one or several unpaired electrons and are trivalent lanthanoids or transition metal ions such as Mn^2+^, Cr^3+^, Co^2+^, Fe^2+^, Fe^3+^, Ni^2+^, and Cu^2+^. Metal-free paramagnetic labels such as nitroxide are another option.^24^ Most of the ^19^F MRI contrast agents developed are predominantly Gd^3+^ complexes with only several instances of Ni^2+^,^25^ Co^2+^,^26^ and Fe^2+^^27^ complexes used in standard contrast agents, while Eu^2+/3+^,^19^ Mn^2+/3+^,^28^ and Co^2+/3+^^29^ were proposed for smart contrast agents along with some investigations focused on other lanthanoids.^20,30^ The research in this field is highly dependent on the experimental discovery of new agents, focusing on the structure of the ligand. Moreover, less attention is paid to the selection of a metal ion based on theoretical calculations for the prediction of relaxation properties. Because the design of new contrast agents must take into account a multitude of factors affecting the relaxation properties, such as the distance and relative position of ^19^F nuclei from the paramagnetic center,^31^ temperature, strength of the magnetic field, and a wide range of possible paramagnetic ions, the use of a reliable theoretical approach is of great value.

Bloch–Redfield–Wangsness (BRW)^32^ relaxation theory is well established and is extensively used for NMR structural studies in biochemistry and in other fields with great success.^33^ Several authors used theoretical calculations to outline design principles of paramagnetic ^19^F contrast agents, especially with regard to the effect of the magnetic field on their properties,^20,27,34^ for analysis of results in terms of contribution of different relaxation mechanisms^26^ or to model experimental results.^32^ BRW relaxation theory was also used in studies of paramagnetic proton NMR relaxation to model the field dependence.^35,36^

**Figure 1:**
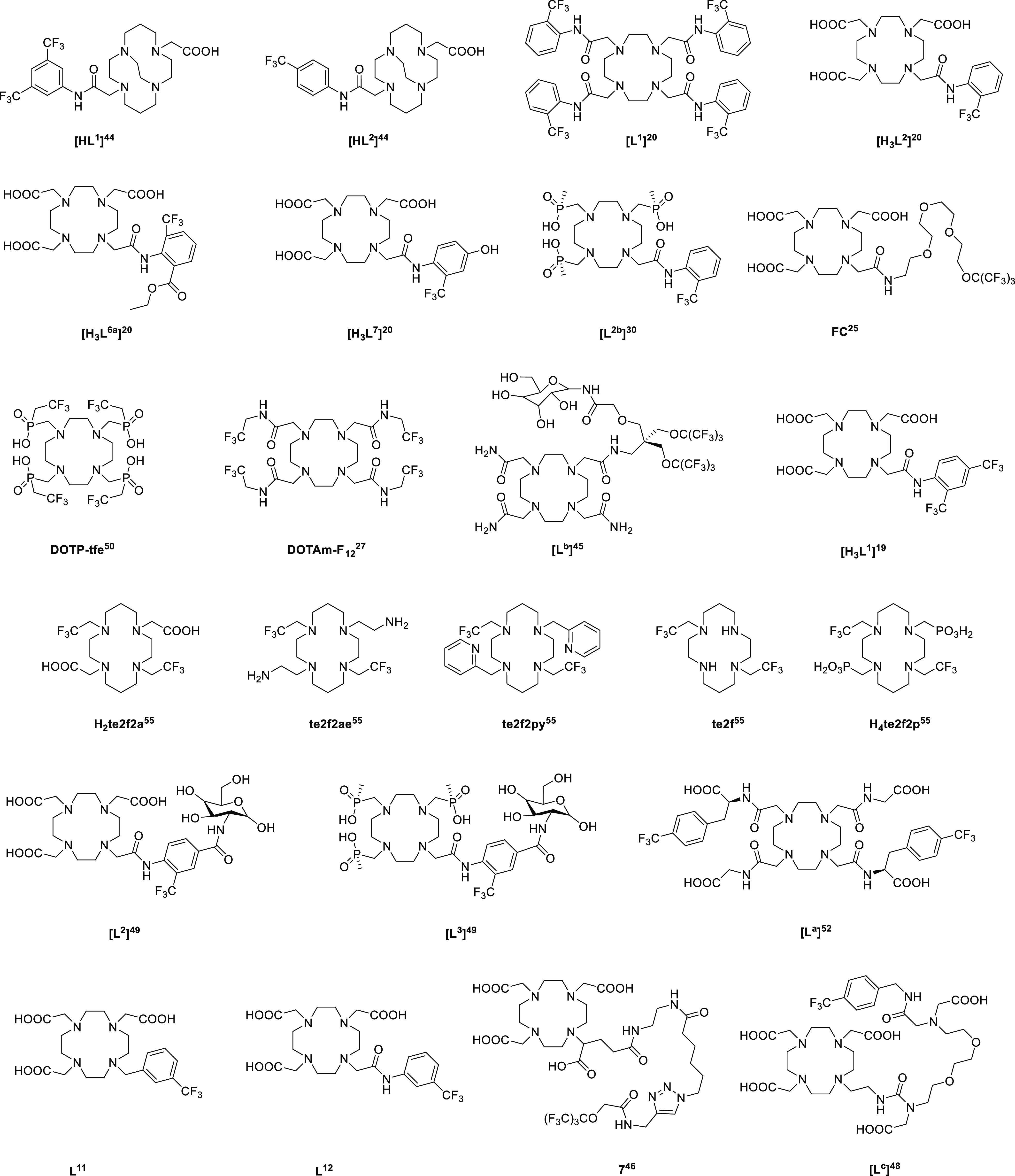
Structures of ligands with available ^19^F relaxation data of corresponding paramagnetic metal ion complexes taken from the literature. Ligands L^11^ and L^12^ were obtained for the purpose of this work.

In this work, we compared ^19^F experimental relaxation data with the predicted values based on BRW relaxation theory to evaluate the reliability of the theoretical calculation. Two series of isostructural complexes of two cyclen-derived ligands, L^11^ and L^12^, were obtained to complement the literature data. L^11^ and L^12^ differed in the number of groups that could participate in the coordination of the metal ion. Their structures are presented in Figure 1. Both ligands are of a similar structure to those most commonly found in the literature. However, they differ in terms of the distance between the fluorine nuclei and the paramagnetic center. In most cases described in the literature, the metal–fluorine (M–F) distances are in the range of 5–7.5 Å and greater than 9 Å. The M–F distances of the L^11^ and L^12^ complexes are 8–9 Å. The proposed structure of L^11^ also allowed the assessment of the effect of chemical exchange on the observed relaxation times and the reliability of theoretical calculations under such conditions. In total, 217 and 143 instances of longitudinal and transverse ^19^F relaxation data, respectively, from the literature and experimental data were collected. The optimized electronic relaxation times and rotational correlation times allowed to further evaluate potential design strategies of various classes of contrast agents. The focus of this study was only on the relaxation rates. The changes in the chemical shift^18,20,27,34^ were not investigated.

## Experimental Section

### Materials

1,4,7,10-Tetraazacyclododecane (95%) was supplied by ABCR GmbH (Karlsruhe, Germany). *tert*-Butyl bromoacetate (99%), 3-(trifluoromethyl)benzyl bromide (99%), and trifluoroacetic acid were supplied by Fluorochem (Glossop, UK). 3-(Trifluoromethyl)aniline (99%), neodymium(III) chloride (99.99%), erbium(III) nitrate pentahydrate (99.99%), and terbium(III) chloride hexahydrate (99.9%) were supplied by Acros Organics (Geel, Belgium). Samarium(III) oxide (99%) and dysprosium(III) oxide (99%) were supplied by Johnson Matthey (UK). Europium(III) chloride hexahydrate (99.99%), gadolinium(III) chloride hexahydrate (99%), holmium(III) chloride hexahydrate (99.9%), and ytterbium(III) trifluoromethanesulfonate (99.99%) were supplied by Sigma-Aldrich (Steinheim, Germany). Praseodymium(III) oxide (99.9%) was supplied by The British Drug Houses (Poole, UK). Manganese(II) chloride tetrahydrate (analytical grade), copper(II) sulfate pentahydrate (reagent grade), nickel(II) chloride hexahydrate (reagent grade), cobalt(II) chloride hexahydrate (reagent grade), ammonium tetrafluoroborate (reagent grade), chromium(III) chloride hexahydrate, iron(II) chloride tetrahydrate (analytical grade), and potassium carbonate (analytical grade) were supplied by POCh (Gliwice, Poland). Cerium(III) nitrate hexahydrate (analytical grade) was supplied by Dr. Theodor Schuchardt GmbH (Munchen, Germany). Iron(III) chloride (reagent grade) was supplied by Fisher Scientific (Loughborough, UK). Yttrium(III) nitrate (99.9%) was supplied by Fluka. Sodium bicarbonate (reagent grade) was supplied by ChemPur (Piekary Slaskie, Poland). DyCl_3_, PrCl_3_, and SmCl_3_ were obtained from the respective oxides in a reaction with an aqueous 3M HCl solution followed by freeze drying.

### Characterization Techniques

The products were characterized using ^1^H and ^13^C NMR in CDCl_3_ or dimethyl sulfoxide (DMSO), while ^19^F NMR spectra were recorded in an aqueous solution (10% D_2_O). The spectra were referenced internally using residual protonated solvent resonances relative to tetramethylsilane (δ = 0 ppm), trifluoroacetic acid (^19^F NMR, δ = −76.5 ppm), or ammonium tetrafluoroborate (δ = −151.5 ppm) as an internal standard. The *T*_1_ and *T*_2_ measurements were performed using inversion recovery and Carr–Purcell–Meiboom–Gill **(**CPMG) sequences, respectively. Samples of complexes for ^19^F NMR relaxation experiments were prepared by mixing 500 μL of aqueous solution (30 mmol dm^–3^) with 50 μL of 22 mmol dm^–3^ aqueous solution of ammonium tetrafluoroborate and 50 μL of D_2_O. An Agilent 400 MR instrument was used for all NMR experiments. High-resolution mass spectrometry studies were performed using a Xevo G2 QTof instrument (Waters) equipped with an electrospray ionization (ESI) source.

### DFT Calculations

All density functional theory (DFT) calculations were performed using Orca 4.2.1 software. Full geometry optimizations of the Gd^3+^ complexes of all investigated ligands were performed in aqueous solution using the hybrid meta-generalized gradient approximation, with the TPSSh exchange correlation functional.^37^ In these calculations, an energy-consistent large-core quasi-relativistic effective core potential and its associated [5s4p3d]-Gaussian type orbital valence basis set for lanthanoids were employed, whereas the ligand atoms were described using the standard 6-31G(d) basis set. Hyperfine coupling tensors (Aiso) for all NMR-active ^19^F nuclei were also calculated in Orca 4.2.1. with a series of hybrid Perdew–Burke–Ernzerhof functionals with the Hartree–Fock exchange set at 30%. The input files and molecular plots were prepared using Avogadro software.^38^ Rotational correlation times were calculated using HYDRONMR^39^ based on the previously optimized structures of Gd^3+^ complexes for all investigated ligands.

### General Synthesis Method

**Scheme 1:**
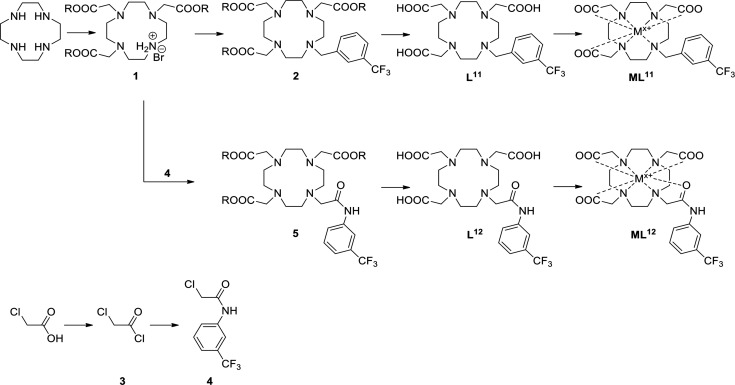
Synthesis of ML^11^ and ML^12^ Metals used to obtain complexes are listed in Table S2. R = *t*-Bu.

The synthesis of the complexes was carried out according to Scheme 1.

### 4,7-Tri(*tert*-butoxycarbonylmethyl)1,4,7,10-tetraazacyclododecane, Hydrobromide Salt (**1**)^40^

40 mL of anhydrous acetonitrile, 2.77 g (33 mmol) of sodium bicarbonate, and 1.72 g (10 mmol) of 1,4,7,10-tetraazacyclododecane were mixed in a 100 mL round bottom flask in an ice bath under argon. Then, 4.81 mL (33 mmol) of *tert*-butyl bromoacetate was slowly added dropwise. The reaction was carried out for 48 h at room temperature and monitored by ultra-performance liquid chromatography (UPLC)-MS. After completion of the reaction, the mixture was filtered, and the solvent was evaporated. The resulting yellow–brown precipitate was recrystallized several times in hot toluene until a white solid was obtained (3.01 g, 45%). ESI-MS: *m*/*z* calculated for C_26_H_51_N_4_O_6_^+^ [M + H]^+^ 515.3809, found 515.3801. ^1^H NMR (400 MHz, CDCl_3_): δ 10.00 (s, 2H), 3.38 (s, 4H), 3.30 (s, 2H), 3.14–3.07 (m, 4H), 2.98–2.83 (m, 12H), 1.49–1.43 (m, 27H). ^13^C NMR (100 MHz, CDCl_3_): δ 170.47, 169.59, 81.80, 81.64, 58.16, 51.33, 49.20, 47.46, 28.20, 28.15.

### 1,4,7-Tris(carboxymethyl)-10-(3-trifluoromethylbenzyl)-1,4,7,10-tetraazacyclododecane (**L**^**11**^)^41^

50 mL of acetonitrile, 2 g (3.9 mmol) of **1**, and 1.5 g (10.9 mmol) of potassium carbonate were added to a 100 mL round bottom flask. The suspension was stirred for 20 min. Then, 0.89 mL of 3-trifluoromethylbenzyl bromide was added dropwise. The reaction was carried out for 24 h at room temperature and monitored by UPLC-MS. After completion of the reaction, the mixture was filtered, and the solvent was evaporated under reduced pressure. The crude product 1,4,7-tris(*tert*-butoxycarbonylmethyl)-10-(3-trifluoromethylbenzyl)-1,4,7,10-tetraazacyclododecane (**2**) was obtained as a yellow oil and used in the next step without purification. ESI-MS: *m*/*z* calculated for C_34_H_55_N_4_O_6_F_3_^+^ [M + H]^+^, 673.4152; found, 673.4140.

10 mL of CH_2_Cl_2_ and 1.5 g (2.2 mmol) of **2** were added to a 50 mL round bottom flask. The solution was stirred for 5 min. Next, 10 mL of concentrated trifluoroacetic acid was added dropwise. The reaction was carried out for 24 h and monitored by UPLC-MS. After completion of the reaction, the solvent was evaporated under reduced pressure. The residue was dissolved in a minimal amount of methanol (≈1 mL) and precipitated with ethyl ether. The resulting brown precipitate was recrystallized several times in a hot ethanol/water (99:1) solution until a white solid was obtained (0.98 g, 88%). ESI-MS: *m*/*z* calculated for C_22_H_31_N_4_O_6_F_3_^+^ [M + H]^+^, 505.2274; found, 505.2267. ^1^H NMR (400 MHz, DMSO-*d*_6_): δ 7.90 (s, 1H), 7.83 (d, 1H, *J* = 8 Hz), 7.77 (d, 1H, *J* = 8 Hz), 7.64 (t, 1H, *J* = 8 Hz), 4.34 (s, 2H), 4.00–2.77 (m, 25H). ^13^C NMR (100 MHz, DMSO): δ 171.7, 158.6 (q, *J* = 30 Hz), 135.6, 130.3, 129.9, 129.6, 128.3, 126.1, 125.8, 123.0, 122.0, 119.0, 116.0, 113.1, 65.3, 56.0, 54.4, 53.7, 50.7, 49.7, 49.2, 48.5.

### Chloroacetyl Chloride (**3**)^42^

SOCl_2_ (9.5 g, 79.9 mmol) and dimethylformamide (1 mL) were added to a 25 mL round bottom flask. Next, chloroacetic acid (5 g, 52.9 mmol) was slowly added. The reaction mixture was stirred for 24 h at room temperature. After the reaction was completed, the solution was distilled under reduced pressure (10 mmHg, 50 °C) to obtain colorless liquid (3.83 g, 64%). ^1^H NMR (400 MHz, CDCl_3_): δ 4.50 (s, 2H). ^13^C NMR (100 MHz, CDCl_3_): δ 167.56, 48.81.

### 2-Chloro-*N*-(3-trifluoromethylphenyl)-acetamide (**4**)^20^

3-Trifluoromethylaniline (0.65 g, 4.03 mmol) and Et_3_N (0.49 g, 4.84 mmol) in anhydrous CH_2_Cl_2_ (10 mL) were added to a 25 mL round bottom flask and placed in an ice bath under argon. Next, chloroacetyl chloride (**3**) (0.55 g, 4.84 mmol) was added slowly to the solution. The reaction was stirred for 12 h and monitored by thin layer chromatography. After the substrates were completely consumed, the solution was washed with 1 M HCl (3 × 10 mL), followed by H_2_O (3 × 10 mL). The organic phase was dried over MgSO_4_, and the solvent was removed under reduced pressure. The crude product was purified using flash silica gel chromatography (CH_2_Cl_2_). The product was obtained as a pale-yellow solid (0.83 g, 87%). mp 73–74.5 °C. ^1^H NMR (400 MHz, CDCl_3_): δ 8.38 (s, 2H), 7.85 (s, 1H), 7.76 (d, *J* = 8.0 Hz, 1H), 7.47 (dd, *J*_1_ = 17 Hz, *J*_2_ = 8.0 Hz, 1H), 7.43 (d, *J* = 8.0 Hz, 1H) 4.21 (s, 2H). ^13^C NMR (100 MHz, CDCl_3_): δ 164.25, 137.35, 131.73, 129.86, 125.18, 123.31, 121.96 (q, *J* = 3.8 Hz), 117.01 (q, *J* = 3.9 Hz), 42.92.

### 1,4,7-Tris(carboxymethyl)-10-[3-(trifluoromethylphenyl)acetamide]-1,4,7,10-tetraazacyclododecane (**L**^**12**^)^20^

2-Chloro-*N*-(3-trifluoromethylphenyl)acetamide (0.167 g, 0.91 mmol) was added to a stirred solution of 1,4,7-tris(*tert*-butoxycarbonylmethyl)-1,4,7,10-tetraazacyclododecane (**1**) (0.30 g, 0.58 mmol), KI (10 mg), and K_2_CO_3_ (0.80 g, 0.58 mmol) in anhydrous CH_3_CN (20 mL) under argon. The mixture was left to boil under reflux for 15 h. The precipitate was removed by filtration, and the residue was washed with CH_2_Cl_2_ (2 × 15 mL). The solvent was removed under reduced pressure, and the resulting solid was purified by silica gel column chromatography (eluent: gradient, 100% CH_2_Cl_2_ to 5% CH_3_OH/CH_2_Cl_2_) to give a pale-orange solid (**5**), which was used in the next step. ESI-MS: *m*/*z* calculated for C_35_H_56_N_5_O_7_F_3_Na^+^ [M + Na]^+^, 738.4030; found, 738.4010.

2.5 mL of CH_2_Cl_2_ and 0.2 g (0.28 mmol) of 1,4,7-tris(*tert*-butoxycarbonylmethyl)-10-(3-(trifluoromethylphenyl)acetamide)-1,4,7,10-tetraazacyclododecane (**5**) were added to a 10 mL round bottom flask. The solution was stirred for 5 min. Next, 2.5 mL of concentrated trifluoroacetic acid was added dropwise. The reaction was carried out for 24 h and was monitored by UPLC-MS. After reaction completion, the solvent was evaporated under reduced pressure. The residue was dissolved in a minimal amount of methanol and precipitated with ethyl ether. The orange precipitate was dissolved in H_2_O. The solution was centrifuged, and the supernatant was lyophilized to yield a pale-yellow solid (0.13 g, 65%). ESI-MS: *m*/*z* calculated for C_24_H_33_N_5_O_7_F_3_^+^ [M + H]^+^, 548.2332; found, 548.2331. ^1^H NMR (400 MHz, DMSO-*d*_6_): δ 12.81 (br s, 3H), 8.29 (s, 1H), 8.1–7.3 (br m, 4H), 4.52 (s, 2H) 4.1–3.0 (br m, 22H). ^19^F NMR (376 MHz, DMSO-*d*_6_): δ −64.08 (s, CF_3_).

### Synthesis of **L**^**11**^ and **L**^**12**^ Complexes^27^

0.02 g of **L**^**11**^ or **L**^**12**^ was dissolved in 10 mL of water/methanol 1:1 solution. A 0.1 M NaOH solution was then added until the pH was ∼7. Then, 1.2 mol % aqueous solution of a metal salt chloride (Nd^3+^, Tb^3+^, Sm^3+^, Dy^3+^, Eu^3+^, Gd^3+^, Ho^3+^, Pr^3+^, Mn^2+^, Ni^2+^, Co^2+^, Cr^3+^, Fe^3+^, and Fe^2+^), nitrate (Er^3+^, Ce^3+^, and Y^3+^), trifluoromethanesulfonate (Yb^3+^), or sulfate (Cu^2+^)] was added to the solution. The reaction was carried out for 24 h at 60 °C, and the pH was adjusted to 7 if needed. The progress was monitored by the UPLC-MS method. After the completion of the reaction, the pH was adjusted to ∼10 with excess NaOH. The precipitated hydroxide was then separated by centrifugation, and the pure complex was obtained by lyophilization.

### Relaxation Data of ^19^F Agents and the Properties of Metal Ions

The effective magnetic moment (μ_eff_) and other physical constants (Table 1) for the calculations were taken from the available literature.^20,43,44^ Relaxation data were also taken from the literature^19,20,25,27,30,45−53^ (Table 2) and supplemented with the measurements of properties for ML^11^ and ML^12^ complexes (Table S2). In most cases, the ^19^F NMR spectrum had one signal from the fluorine nuclei. In the case where more signals were observed, only the one with the highest intensity was considered. Ions lacking literature data, such as Cr^3+^, and the investigation into the impact of chemical exchange in the case of the heptadentate ligand L^11^ were of particular interest. The literature search was focused on non-Gd^3+^ complexes, especially when isostructural series or multiple field data were available. R_2_ data were available for about 50% of complexes. Diamagnetic references were scarcely used.

**Table 1:** Physical Constants Used for Relaxation Data Analysis^54^

μ_0_	1.25663706212 × 10^–6^ [H m^–1^]	vacuum permeability
γ_F_	251.8148 × 10^6^ [rad s^–1^ T^–1^]	magnetogyric ratio of the fluorine nuclei
h̵	1.05457181313131 × 10^–34^ [J s]	reduced Planck constant
k_B_	1.38065 × 10^–23^ [J K^–1^]	Boltzmann constant
T	300 [K]	temperature
g_J_	2.00232	electron *g*-factor
μ_B_	9.274 009 994 × 10^–24^ [J T^–1^]	Bohr magneton

**Table 2:** Literature Data Concerning ^19^F Relaxation and the Properties of Paramagnetic Ions

Metal ion	μ_eff_/μ_B_^43,54,55^	S	*T*_1e_ [s] literature data^43,54,55^	field range [T]	sources of ^19^F relaxation data
Ce^3+^	2.55	5/2	1 × 10^–13^	7–11.7	(25, 51)^a^
Co^2+^	4.7	3/2	10^–11^ to 10^–13^ (HS)	1.4–9.4	(46)^a^
			10^–9^ to 10^–10^ (LS)		
Cr^3+^	3.8	3/2	5 × 10^–9^ to 1 × 10^–10^	9.4	a
Cu^2+^	1.9	1/2	(1–5) × 10^–9^	9.4–11.7	(25)^a^
Dy^3+^	10.3	15/2	10^–12^ to 10^–13^	4.7–16.5	(19, 20, 25, 27, 30, 47−51)^a^
Er^3+^	9.4	15/2	1 × 10^–13^	4.7–16.5	(20, 25, 27, 30, 48)^a^
Eu^3+^	3.5	7/2	1 × 10^–13^	5.9–11.7	(25, 27, 47, 49, 53)^a^
Fe^2+^	5.4	2	10^–12^ to 10^–13^ (HS)	1.4–9.4	(27, 46)^a^
Fe^3+^	7	5/2	10^–11^ to 10^–13^ (LS)	9.4–11.7	(25)^a^
			10^–9^ to 10^–10^ (HS)		
Gd^3+^	7.63	7/2	1 × 10^–8^	5.9–11.7	(25, 27, 47, 49, 53)^a^
Ho^3+^	10.4	8	1 × 10^–13^	4.7–16.5	(19, 20, 25, 27, 30, 48, 50, 51)^a^
Mn^2+^	5.8	5/2	1 × 10^–8^	9.4	a
Nd^3+^	3.69	9/2	1 × 10^–13^	9.4–11.7	(25)^a^
Ni^2+^	3.5	1	10^–10^ to 10^–12^	0.94–11.7	(25, 45, 46, 56)^a^
Pr^3+^	3.47	4	10^–13^ to 10^–14^	9.4–11.7	(25)^a^
Sm^3+^	1.58	5/2	10^–13^ to 10^–14^	9.4–11.7	(25)^a^
Tb^3+^	9.8	6	1 × 10^–13^	4.7–11.7	(19, 20, 25, 27, 30, 47, 49, 53)^a^
Tm^3+^	7.6	6	10^–12^ to 10^–13^	4.7–16.5	(19, 20, 27, 30, 48, 49, 51)
Yb^3+^	4.7	7/2	1 × 10^–13^	4.7–11.7	(19, 25, 27, 51)^a^

a ML^11^ and ML^12^ data obtained based on our study (Table S2). μ_eff_—effective magnetic moment, S—spin–spin coupling, *T*_1e_—electronic relaxation time, LS—low spin, and HS—high spin.

## Results and Discussion

### Relaxation Theory

According to BRW theory,^57−59^ the relaxation of ^19^F nuclei in the presence of paramagnetic species in non-viscous solutions occurs mainly due to five distinct mechanisms. The most important mechanisms are the electron–nucleus dipole–dipole (DD) interaction and Curie (Cur) relaxation given by
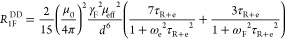
1
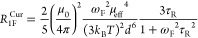
2
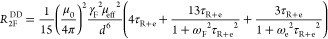
3
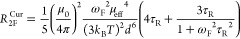
4

The Curie relaxation is typically treated as isotropic, but lanthanoid-induced nuclear relaxation is anisotropic.^60^ The effect is more significant for ^13^C and ^15^N compared to that for ^1^H or ^19^F, and these contrast agents strongly affect the relaxation rates of nuclei less than 4 Å away from the paramagnetic ion.^60−62^ Because the M–F distance in all of the investigated ligands was greater than 5 Å, the anisotropy was not factored into the calculations. Potentially, the significance of anisotropy can be identified if the distance calculated from relaxation data substantially deviates from the DFT or X-ray distances. However, in PRE measurements, these deviations are not typically observed.^62,63^

The third mechanism of ^19^F nucleus relaxation is based on contact interactions (Con) which may be significant in the case of transverse (*T*_2_) relaxation of fluoroorganic complexes of d-elements. Con is given by
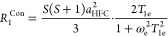
5
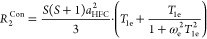
6

In the specific case of ^19^F relaxation in the lanthanoid complexes,  contributions may safely be ignored.^20^ Fermi contact hyperfine coupling constants (*a*_HFC_) can be obtained from quantum chemistry calculations if a structure of a complex is known.^27^ It is worth noting that *a*_HFC_ in rad·s^–1^ is used in eqs 5 and 6. In the case of d-metals, this mechanism usually contributes 1–2 Hz in relaxation rates and is often ignored. The significance of Con relaxation can be easily identified if predicted *R*_2_ is strongly underestimated compared to accurately predicted *R*_1_. This is because of the greater impact of the contact mechanism on *R*_2_^Con^ than on *R*_1_^Con^ (eq 7, Table 3).
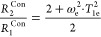
7

**Table 3:** Ratio of Transverse and Longitudinal Contact Relaxation Rates (Eq 7) in the d-Metal Complexes Assuming Non-Zero a_HFC_^a^

metal ion	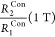	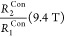
Cr^3+^	2.5 × 10^3^	2.2 × 10^5^
Mn^2+^	1.8 × 10^6^	4.2 × 10^9^
Fe^3+^	27	2.3 × 10^3^
Fe^2+^	1.3	106
Co^2+^	1.8	68
Ni^2+^	1.3	6.0 × 10^3^
Cu^2+^	3.9 × 10^5^	3.4 × 10^7^

a Electronic relaxation times were taken from Table 4.

The remaining two mechanisms, chemical shift anisotropy and internuclear dipole–dipole interaction, are minor contributors to BRW theory and can be ignored in most ^19^F PRE applications.^20^ Special attention should be paid to cross-correlation effects that are ignored by BRW theory. These cross-correlation effects occur due to the simultaneous presence of multiple relaxation mechanisms such as dipolar, Curie spin, and chemical shift anisotropy relaxations. The importance of cross-correlation effects can be estimated from various two-dimensional NMR experiments^64^ or observed in relaxation rate measurements in solution structure refinement of proteins.^65^ The greatest cross-correlation effects can be expected when the Curie spin relaxation mechanism plays a dominant role. This is the case of paramagnetic ions with rapidly relaxing electronic spins (i.e., Dy^3+^ and Yb^3+^).^66^ Transverse relaxation times are affected more than the longitudinal relaxation times by cross-correlation effects and are both distance- and angular-dependent.^67^ Overall, experimental *R*_2_ can differ from predicted values depending on the relative position of the paramagnetic center and particular nuclei.^65^

The chemical shift anisotropy–anisotropic dipolar shielding cross-correlation (CSA × DSA) is the most important one, especially when rotational correlation times are greater than 1–5 ns.^66^ In the case of low-molecular weight complexes of τ_r_ ≈ 0.25 ns, relaxation rates *R*_1_ and *R*_2_ arising from CSA×DSA were estimated for a series of putative lanthanoid complexes using eqs S2–S5 assuming an anisotropy of the chemical shift tensor  of 100 ppm.^68^ The CSA × DSA cross-correlation effect is negligible at 1–3 T, but its importance increases with the magnetic field. The results at 9.4 T (Tables S3 and S4) indicate that depending on the angle between the principal axes of chemical shift anisotropy and dipolar shielding anisotropy tensors θ^(CSA,DSA)^, the cross-correlation effect can be responsible for the increase or the decrease in longitudinal and transverse relaxation rates by up to 7 Hz for ions having a small effective magnetic moment (Ce^3+^, Pr^3+^, Nd^3+^, Sm^3+^, and Eu^3+^). In the case of ions exerting strong PRE (Tb^3+^, Dy^3+^, Ho^3+^, Er^3+^, and Tm^3+^), the effect is higher and can reach 40 and 60 Hz for longitudinal and transverse relaxation rates, respectively. Comparing with the contribution of contact and Curie spin relaxation (Table S5), this means that the CSA × DSA cross-correlation effect can dominate at large distances (10 Å) between the paramagnetic ion and fluorine nuclei regardless of the metal ion. At short, typically observed, distances (6 Å), the effect can contribute at most 10% in the case of Tb^3+^, Dy^3+^, Ho^3+^, Er^3+^, and Tm^3+^. Such high contributions are possible only for θ^(CSA,DSA)^ close to 0 or 90° with zero contribution at 54.7°. For this reason, the cross-correlation effects were not considered in this work. Additionally, relaxation data of a single ^19^F resonance do not allow deconvolution of potential cross-correlation effects from the dipolar and Curie spin relaxation rate enhancements if the distance, correlation time, and θ^(CSA,DSA)^ must be found by data fitting.

The relaxation rates resulting from various relaxation mechanisms are additive
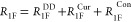
8
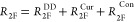
9

The remaining variables are given by:
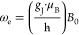
10
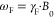
11
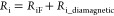
12
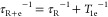
13
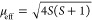
14where *T* is the temperature, ω_e_ and ω_F_ are the electron and nuclear Larmor angular frequencies, respectively, and the physical constants have their usual meaning. *R*_i_diamagnetic_ (eq 12) are the relaxation rates observed for complexes containing nonparamagnetic metals such as La^3+^ or Y^3+^. These rates can be directly measured or extrapolated from relaxation rates in a series of isostructural complexes of different paramagnetic ions, and they are typically 1 and 2 Hz or greater for *R*_1_ and *R*_2_, respectively. When the effect of PRE is significantly large (*R*_iF_ > 50 Hz), then the diamagnetic contribution can be ignored. Equation 13 defines correlation time (τ_R+e_) and is valid in solution in the absence of chemical exchange. Note that this equation was frequently misprinted in the literature without the multiplicative inverse of the left side.

The effective magnetic moment (μ_eff_) can be calculated based on eq 14. However, such approximation is not generally valid because strong ligand field effects exist.^32,35,36,61^ Effective magnetic moments are usually determined experimentally using the Evans method^69^ or SQUID magnetometry^70^ for a given compound. Most authors use tabularized data (Table 2) obtained from the measurements of various macrocyclic complexes of paramagnetic ions. The same approach was used here. In this way, the ligand effects were at least partially included in magnetic moment calculations. The use of tabularized data is also justified due to great structural similarities between investigated fluoroorganic ligands. The remaining parameters that are necessary to predict relaxation rates are the following: electronic relaxation time (*T*_1e_), rotational correlation time (τ_R_), and metal–nucleus distances (*d*). These parameters are usually obtained during independent experiments or derived from relaxation data.

### Fitting of the Experimental Relaxation Data to BRW Theoretical Equations

An iterative fitting procedure of the experimental relaxation data was performed using BRW theoretical eqs 1–4. *T*_1e_, τ_R_, and d were allowed to change. An agreement between experimental and predicted log *R*_1_ and log *R*_2_ with equal weighting was sought (eq S1). The boundaries of *T*_1e_ were 0.1–9 times the literature values. τ_R_ values ranged from 0.1 to 0.9 ns, and d was ±1 Å of the expected M–F distance (calculated using DFT for Gd^3+^ complexes or taken from X-ray data). In the case of ligands where the data did not produce well defined minima during the fitting procedure, τ_R_ was fixed at 0.25 ns, or the exact distance was taken from X-ray data when available. This occurred in the absence of field-dependent data when only a single metal complex was investigated with a particular ligand. In the second step, resulting *T*_1e_ was fitted to eq 15. In this equation, the correlation time (τ_v_) indicated the field dependency, whereas τ_s0_ was the correlation time under the zero field.^71^
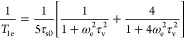
15

The field dependence of *T*_1e_ was determined for Dy^3+^, Er^3+^, Tm^3+^, Tb^3+^, Ho^3+^, Ce^3+^, Ni^2+^, Co^2+^, and Fe^2+^. For other metals, single T_1e_ was obtained as an estimate within 4.7–11.7 T (Table 4). In the next iteration, only τ_R_ and *d* were optimized again. Finally, *d* was allowed to vary by ±0.5 Å from the average distance for each individual complex. For selected complexes displaying significantly greater observed *R*_2_ compared to the calculated value, contact interaction (*a*_HFC_) was determined by data fitting (Table S6). There was no systematic deviation between the observed and predicted data for any metal ion or ligand. However, the *R*_2_ data were predicted with lower precision overall.

**Table 4:** Calculated Values of τ_s0_, τ_v_ (According to Eq 15 and ^19^F Relaxation Data), and Resultant *T*_1e_^a^

metal ion	τ_s0_ [ps]^b^	τ_v_ [ps]^b^	*T*_1e_(9.4 T) [s]
Cr^3+^	395 ± 50		4 × 10^–10^
Mn^2+^	55000 ± 5000		5.5 × 10^–8^
Fe^3+^	39 ± 1		3.9 × 10^–11^
Fe^2+^	1.9 ± 0.06	1.0 ± 0.03	1.6 × 10^–11^
Co^2+^	0.9 ± 0.03	0.5 ± 0.03	2.5 × 10^–12^
Ni^2+^	1.7 ± 0.06	3.8 ± 0.08	1.7 × 10^–10^
Cu^2+^	5000 ± 100		5 × 10^–9^
Ce^3+^	0.1 ± 0.03	0.7 ± 0.2	5.4 × 10^–13^
Pr^3+^	0.1 ± 0.01		9.9 × 10^–14^
Nd^3+^	0.3 ± 0.01		2.8 × 10^–13^
Sm^3+^	0.09 ± 0.01		9 × 10^–14^
Eu^3+^	0.1 ± 0.01		1 × 10^–14^
Gd^3+^	10000 ± 5000		1 × 10^–8^
Tb^3+^	1.4 ± 0.03	0.5 ± 0.05	4.3 × 10^–12^
Dy^3+^	1.2 ± 0.06	0.7 ± 0.03	5.6 × 10^–12^
Ho^3+^	0.2 ± 0.01	1.4 ± 0.04	2.9 × 10^–12^
Er^3+^	0.2 ± 0.04	1.4 ± 0.07	3.5 × 10^–12^
Tm^3+^	0.5 ± 0.05	0.9 ± 0.06	3.5 × 10^–12^
Yb^3+^	0.3 ± 0.01		2.6 × 10^–12^

a Missing τ_v_ values indicate the lack of data for the calculation of field dependency of electronic relaxation time.

b Uncertainties correspond to the maximal change of the loss function of 1% resulting from changes in τ_s0_ or τ_v_.

### Electronic Relaxation times

It is necessary to emphasize that transverse and longitudinal relaxation times are field- and ligand-dependent.^72−74^ Ligand dependence was ignored, and only the average values of *T*_1e_ suitable for predicting the properties of typical fluoroorganic complexes were calculated. The theoretical expressions (eqs 1–6) given above are more complicated if *T*_1e_ ≠ *T*_2e_. However, the existence of a single (average) electronic relaxation time was assumed. In the literature, only approximate values of *T*_1e_ are often presented for a given metal ion. Otherwise, *T*_1e_ values are calculated based on BRW equations for each case to achieve the best fit between observed and predicted relaxation rates. The most precise values can be determined from nuclear magnetic relaxation dispersion data^72^ or from relaxation studies using several instruments encompassing a wide range of magnetic fields. For most of the collected literature regarding ^19^F relaxation data, the effect of the magnetic field on *T*_1e_ could be ignored. However, in the case of Ni^2+^ complexes,^52^ no reasonable agreement between the predicted and experimental values could be achieved without taking into account the field dependency of *T*_1e_. Based on the τ_s0_ and τ_v_ values (Table 4), the electronic relaxation time (*T*_1e_) can be calculated for any field strength. On average, resultant *T*_1e_ at 1 and 16.5 T is about 20 and 250% of the value at 9.4 T, respectively.

### Rotation Correlation Times

**Figure 2:**
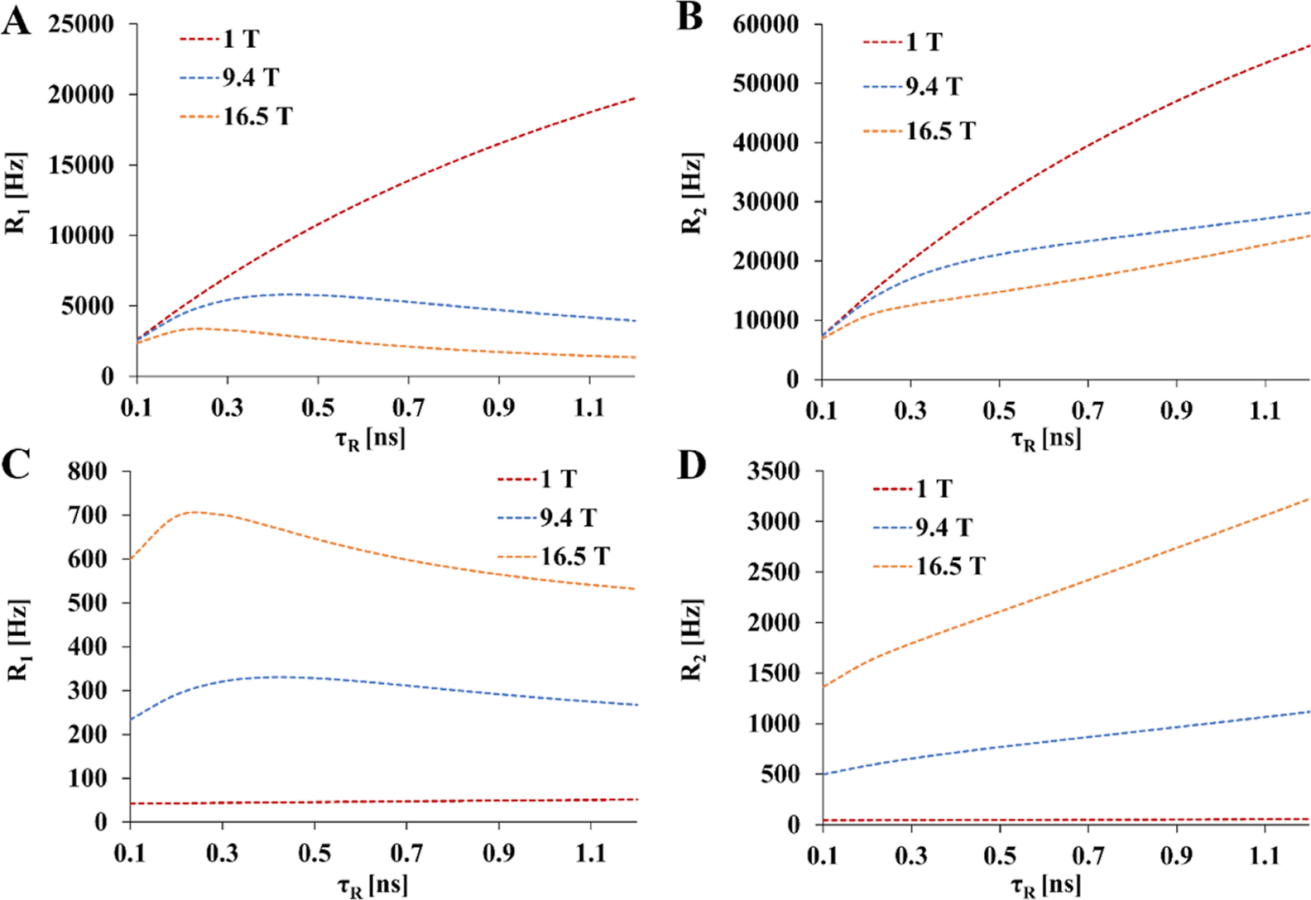
Relationship between rotational correlation time and predicted ^19^F relaxation rates for hypothetical paramagnetic complexes: (A) R_1_ Gd^3+^, (B) R_2_ Gd^3+^, (C) R_1_ Ho^3+^, and (D) R_2_ Ho^3+^. Distance: 6 Å. Diamagnetic reference: *T*_1_ = 1 s and *T*_2_ = 0.5 s. The mathematical model encompasses eqs 1–4, 12, and 15. Electronic relaxation data from Table 4.

τ_R_ is associated with the rotation of a molecule and is dependent on the size, shape, and molecular dynamics. τ_R_ is often estimated using the Stokes–Einstein–Debye equation,^75^ and one may add an appropriate shape correction factor^76^ for non-spherical molecules. More sophisticated hydrodynamic calculations based on the bead model (implemented in the HYDRONMR program) have become popular recently.^39^ Another approach for calculating τ_R_ relies on field-dependent relaxation data that enable experimental determination of τ_R_ for a given compound by fitting the data to BRW theoretical equations.^20^ This approach was used here. In the absence of such data, *R*_1_ and *R*_2_ results measured in a single magnetic field, especially from a series of isostructural complexes, still enable sufficient estimates of τ_R_. If only the *R*_1_ data are available, the fitting procedure is not reliable without knowledge of T_1e_ and *d.* This is illustrated in Figure 2. Typically, there are two values of τ_R_ with the same predicted longitudinal relaxation rate, but *R*_2_ data show a monotonous increase in *R*_2_, whereas the increase in τ_R_ is in the 0.1–1 ns range. The pattern is strongly field-dependent.

Unfortunately, τ_R_ obtained from HYDRONMR is always greater than values calculated using the Stokes–Einstein–Debye equation, and even lower values are obtained by fitting the ^19^F relaxation data for the same molecule (Table S1). One can determine τ_R_ from ^1^H NMR relaxation dispersion (NMRD) data, but this procedure is known to underestimate its value.^20^ For low-molecular weight dodecane tetraacetic acid (DOTA) Gd complexes, correlation times near 0.1 ns are typically reported from ^1^H NMRD data.^77^ Other heteronuclear NMR measurements can also be used, but the resulting values would likely be different due to differences in mobility between functional groups. For example, from the field variable ^31^P relaxation data of complexes of a comparable structure, ≈0.3 ns correlation times were identified,^32^ whereas rather high values (1–2 ns) were determined for a Cd^2+^ complex with a molecular weight of 454 g mol^–1^ from ^13^C relaxation data.^78^

When analyzing the results of ^19^F relaxation data fitting to the theoretical equations for DOTA-type paramagnetic complexes, the average value of the rotational correlation time was 0.25 ns regardless of the type of −CF_3_ group (aromatic or aliphatic) or the size of the complex (Table S1). This rotational correlation time value could be used for prediction of relaxation rates for all investigated complexes leading to sufficient agreement between the predicted and experimental data. However, an exception was the macromolecular Ho^3+^ [L^2^-chitosan] complexes^50^ which exhibited a longer correlation time (3.8 ns). Interestingly, in the case of similar Dy^3+^ complexes of [L^3^-chitosan], the calculated correlation time was 0.2 ns (Table S1).

### Metal–Nucleus Distances

**Figure 3:**
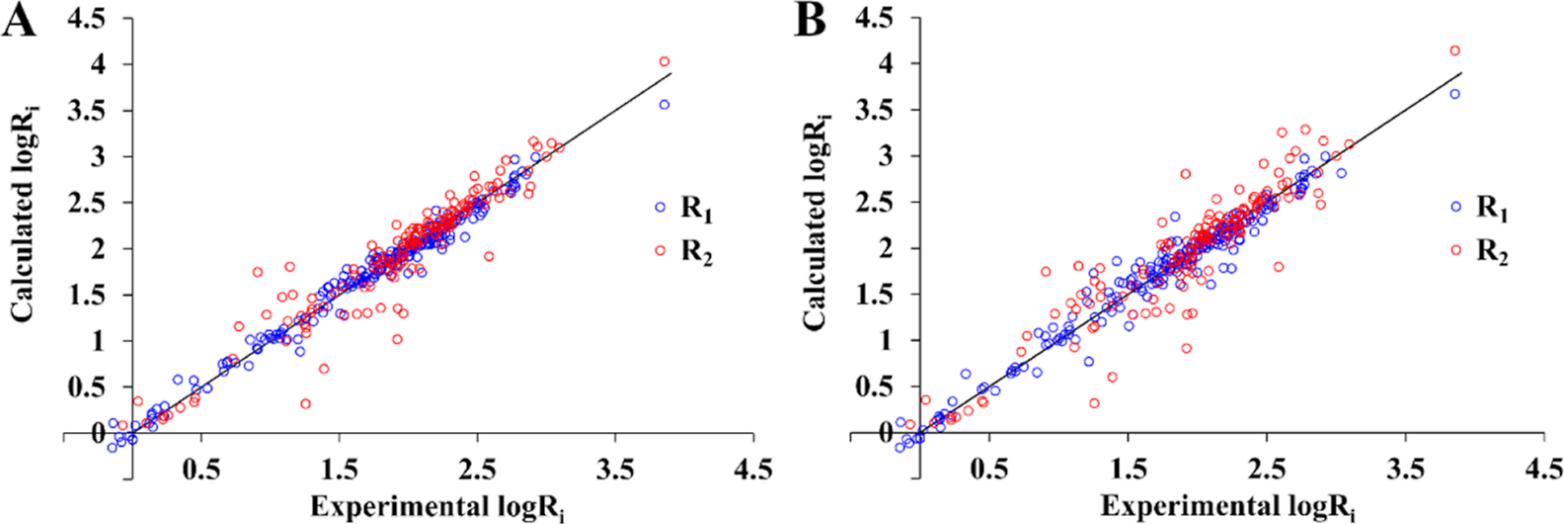
Comparison between observed and predicted *R*_1_ and *R*_2_ data with optimized distances for each individual complex (A) or only optimized average distances for each ligand from Table S1 (B). *T*_1e_ values calculated from Table 4. τ_R_ values were taken from Table S1. The mathematical model encompasses eqs 1–6, 12, and 15.

The metal–nucleus distance (*d*) is the most important factor that affects the relaxation properties. This distance can be determined from the quantum chemistry calculations, but such data should be treated with caution because of the potential existence of a conformer that is different, more stable than the one identified by DFT calculations. X-ray data lack this shortcoming, but in both cases, the observed M–F distances of different fluorine atoms of a CF_3_ group must be averaged. The correct approach is to average the calculated d^–6^ values. Otherwise, the distance can be overestimated because fluorines closer to the paramagnetic center have a greater impact on the observed relaxation rates. This is especially evident for the complex developed by Yu et al.^46^ where the average metal–nucleus distance calculated from the DFT-optimized structure for the Gd^3+^ complex was 8.4 Å, whereas the average of (*d*^–6^)^−1/6^ was 7.8 Å. For comparison, the distance calculated from BRW equations was 7.3 Å ([L^b^]—Table S1). In most investigated cases, the difference was less than 0.5 Å. The average distances can also be determined from relaxation data in a series of isostructural complexes, but the M–F distances vary considerably between metals. For example, in a complex [L^2b^],^30^ the DFT distances were between 6.1 Å (Gd^3+^) and 6.7 Å (Fe^3+^) (6.3–6.4 Å on average for other metals). The distances obtained using DFT (using Gd^3+^ as the model) were within 1 Å of those calculated by fitting relaxation data to theoretical equations (Figure S6). During the fitting procedure, the assumption of a constant distance in a series of complexes was initially assumed, leading to sufficient agreement between the predicted and experimental data. When the assumption was dropped and the distances were allowed to vary by ±0.5 Å from the mean value, the accuracy further improved (Figure 3A).

### Fermi Contact Hyperfine Coupling Constants *a*_HFC_

Among the published ^19^F relaxation data, the contact mechanism was explicitly identified for some Co^2+^ complexes.^26^ Examining all available data with respect to the differences between predicted and experimental relaxation rates indicated that the contact mechanism was potentially significant in the case of several complexes of Cu^2+^(L^12^ and FC-Cu^2+^),^25^ Fe^3+^(FC–Fe^3+^),^25^ Co^2+^ (L^11^), and Mn^2+^ (L^12^). The resulting *a*_HFC_ values from the fitting procedure were only partially confirmed by DFT calculations (Table S6), but in most cases, DFT yielded *a*_HFC_ ≈ 0. These results could be a consequence of higher uncertainties of *R*_2_ data or a contribution of other mechanisms of relaxation such as chemical exchange line broadening^79^ which was significant in the case of L^11^ complexes.

The practical significance of the contact mechanism in the design of contrast agents is illustrated in Table 3 which was based on eq 7 and the optimized electronic relaxation times (Table 4). In the case where field-dependent data were missing, *T*_1e_ at 1 T was assumed to be ≈20% of the value at 9.4 T. Because the transverse contact relaxation rate can be several orders of magnitude greater than the longitudinal relaxation rate, this mechanism should be especially considered for Mn^2+^, Cr^3+^, and Cu^2+^ and to a lesser extent for Ni^2+^, Fe^2+^, Fe^3+^, and Co^2+^. The field dependence of the contact mechanism is also evident, which should be considered during the development of new contrast agents. Overall, the contribution of the contact mechanism is comparable to the diamagnetic contribution in *R*_2_ rates, but the contact mechanism is not significant in the case of *R*_1_ rates. In most of the investigated cases, the dipolar and Curie mechanisms dominate and are responsible for at least 90% of PRE. However, a greater contribution of the contact mechanism in R_2_ relaxation was found in the case of Cu^2+^, Ni^2+^, and Mn^2+^ complexes (Figure S5).

### Agreement between Predicted and Observed Results

The reliability of the theoretical calculations for *R*_2_ and *R*_1_ is presented in Figure 3. The calculated data were generally consistent with experimental values regardless of the metal or ligand type. The deviations of *R*_1_ were typically ±25% (Figure S3A) with several instances where the deviations reached +125 or −40%. In the case of *R*_2_, the consistency was lower. However, in most cases, the *R*_2_ values were within the ±50% range.

It is worth noting that many of the investigated complexes displayed relaxation rates similar to their respective diamagnetic references. Additionally, even small absolute differences between the calculated and observed rates resulted in large relative deviations. The highest discrepancies of *R*_1_ occurred in the cases of Gd^3+^, Fe^3+^, and Mn^2+^. In the case of *R*_2_, Pr^3+^ and Nd^3+^ had the highest discrepancies. In the case of Gd^3+^, these discrepancies were due to a very strong PRE effect and frequent difficulties in measuring very short relaxation times (<1 ms). Therefore, even a small underestimation or overestimation of relaxation times translates into significant differences between calculations and observed values. The case of iron is more complex due to the two possible oxidation and spin states with different properties that might coexist in the sample. An analogous situation can occur in europium complexes. The contamination with ferrimagnetic FeO·Fe_2_O_3_, which may form during complexation under basic conditions, is another possibility that contributes to measurement uncertainty and higher experimental relaxation rates. Another potential factor is the high contribution of other relaxation mechanisms, especially the chemical exchange line broadening.^79^ This is in agreement with the short relaxation times observed by Jiang et al. in complexes of several diamagnetic ions (Bi^3+^, Pb^3+^, and Hg^2+^).^25^ This is particularly visible in the case of our data for the DO3A-based ligand L^11^. In this case, the diamagnetic reference showed the highest observed *R*_2_ value (19 Hz, Table S2) indicating strong line broadening due to the free coordination site of the metal. The reliability of the calculations was also much lower compared to that of the corresponding complexes of the L^12^ ligand (Figure S4). Another potential factor is the contribution of chemical shift anisotropy–anisotropic dipolar shielding cross-correlation that may lead to either over- or underestimation of the calculated relaxation rates. Quantification of this effect would require the estimation of at least one additional variable [θ^(CSA,DSA)^] from only ^19^F *R*_1_ and *R*_2_ experimental data. This would require prior knowledge of τ_R_ or the F-metal distance in particular complex or multiple magnetic field relaxation data. Such data were not available for most complexes.

### Uncertainties of Predicted and Experimental Data

The uncertainties of the relaxation rate measurements are typically 5% when INVERC or CPMG sequences are used. This translates into 0.1 Å uncertainty of *d* if it is calculated from relaxation data assuming that *T*_1e_ and τ_R_ values are known. Conversely, the knowledge of *d* is critical for the accurate prediction of relaxation rates which are impacted by *T*_1e_, τ_R_, or μ_eff_ to a lesser degree. This is illustrated in Table 5 for a series of model lanthanoid complexes wherein the M–F distance was 6 Å. In all cases, even a small, 2% difference (0.1 Å) can be the sole factor contributing to observed uncertainty despite much larger assumed uncertainties of *T*_1e_ or τ_R_. The magnetic moment is also potentially important if actual μ_eff_ is different from the assumed one.

**Table 5:** Contribution of the Main Factors to the Overall Uncertainty of the Predicted Relaxation Rates from BRW Theoretical Equations

ion		distance [Å]	μ_eff_/μ_B_	τ_R_ [ns]	*T*_1e_ [s]	calculated *R*_1_
Gd^3+^	Properties	6.0	7.6	0.25	1 × 10^–8^	58,000 ± 600
	assumed uncertainty	2%	2%	40%	50%	
	contribution to the error of *R*_i_	99.9%	<0.1%	<0.1%	<0.1%	
Ho^3+^	properties	6.0	10.4	0.25	1.5 × 10^–12^	1891 ± 20
	assumed uncertainty	2%	2%	40%	50%	
	contribution to the error of *R*_i_	99.7%	0.3%	<0.1%	<0.1%	
Nd^3+^	properties	6.0	3.7	0.25	2.5 × 10^–13^	72 ± 1
	assumed uncertainty	2%	2%	40%	50%	
	contribution to the error of *R*_i_	99.9%	<0.1%	<0.1%	<0.1%	

As a practical conclusion, the two-significant digit precision of *T*_1e_ and τ_r_ is sufficient for the calculation of *R*_i_. Such limits are justified by the predicted impact of changes in *T*_1e_ and τ_r_ on relaxation times over a broad range of fields and effective magnetic moments (Figure 2). In the case of varying *T*_1e_, the relaxation rates (at 9.4 T) are only minimally affected if *T*_1e_ is either greater than 10^–9^ s or less than 10^–11^ s which is true for most of the paramagnetic ions except for Fe^2+/3+^ and Ni^2+^ (Table 4). The impact of rotational correlation time and field strength on the calculated relaxation times determined for a broad range of metals is illustrated in Figure 2. The results indicate that 50% changes in rotational correlation time translate into at least 20% changes of the resulting theoretical *R*_1_ and *R*_2_ but only under high field strength or when a paramagnetic ion with high effective magnetic moments is used regardless of the field strength. The data fitting procedure indicated that the 0.01 ns change of τ_r_ usually translated into 1% of the change in the loss function, and very narrow minima were observed, especially when multiple field relaxation data were available. Similarly, 0.01 Å changes in *d* produced a similar 1% change of the loss function (Table S1, eq S1). If such precision is used instead of the assumptions in Table 5, the uncertainty of *R*_1_ would be 5–6 times lower, and the contribution of distance to the overall uncertainty of *R*_1_ would only be 8–50% depending on the metal.

### Potential Design Strategies for Relaxometric Contrast Agents

The optimization procedure produced τ_v_, τ_s0_, and τ_r_ values that could be used to predict the properties of any contrast agent if the structure does not deviate significantly from the typical DOTA-type and contains the −CF_3_ group as a tag. For such compounds, constant τ_R_ (0.25 ns) can be reasonably expected, and the field dependence of *T*_1e_ should be considered. Weaker and stronger magnetic fields were studied separately because they reflect the conditions in medical and research-based MR scanners, respectively.

### Effect of Metal Ions on

#### ^19^F Relaxation at a Fixed Distance

**Figure 4:**
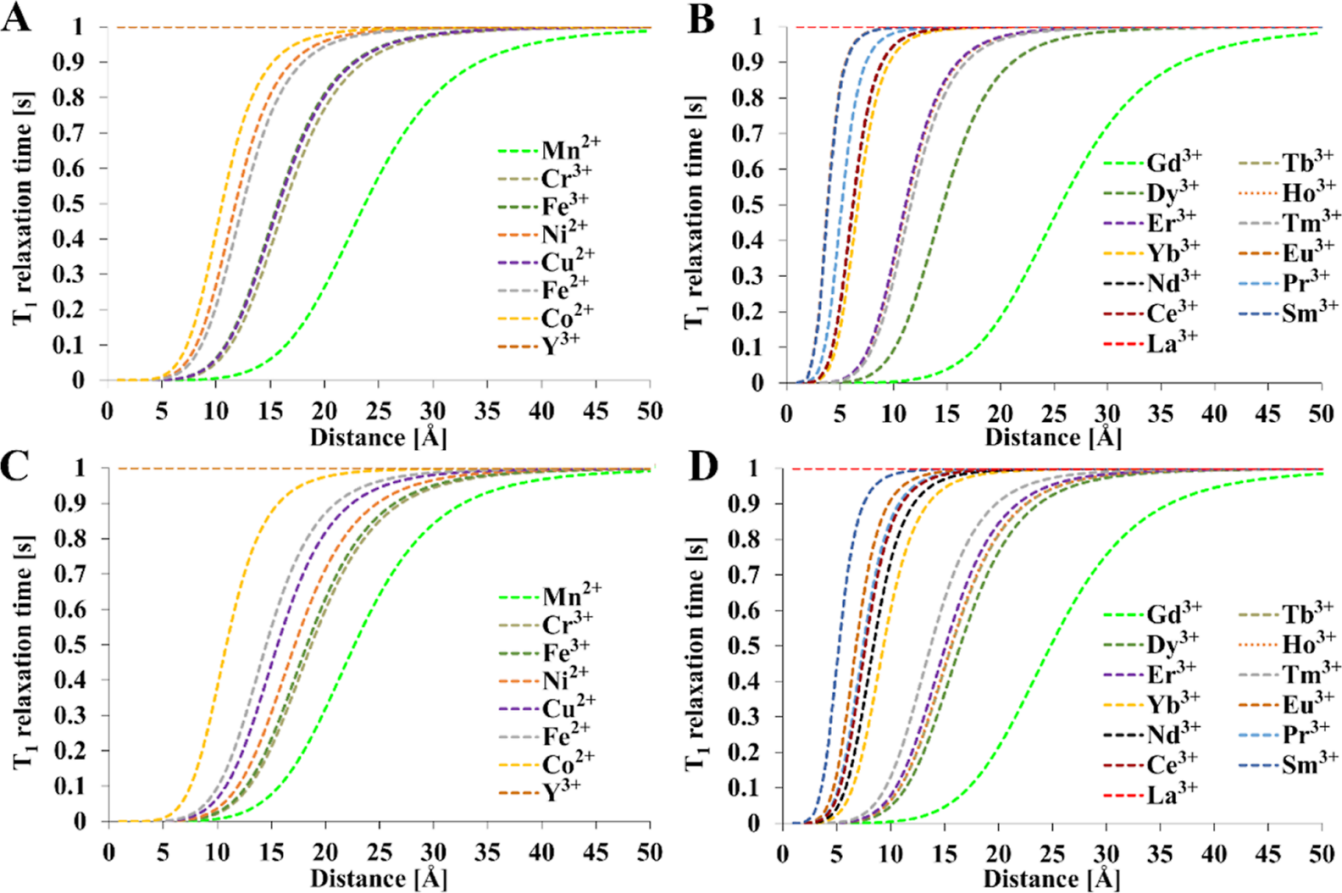
Theoretical relationship between the metal–fluorine nucleus distance and the longitudinal relaxation time [(A,B)—1 T and (C,D)—9.4 T]. Relaxation times of diamagnetic references: *T*_1_ = 1 s and *T*_2_ = 0.5 s, *T* = 300 K, τ_R_ = 0.25 ns. *T*_1e_ values were calculated from Table 4. Mathematical model encompasses eqs 1–4, 12, and 15.

To evaluate the suitability of various paramagnetic ions as components of ^19^F contrast agents, the first set of calculations aimed to demonstrate the impact of the ion type and distance on the relaxation time of the ^19^F nuclei due to the PRE effect. No further assumptions were made about the structure of the ligand. The literature values for the diamagnetic Y^3+^ or La^3+^ complexes were in the range of 0.5–1.5 s and 0.25–0.8 s for *T*_1_ and *T*_2_, respectively, and the mean values of 1 s and 0.5 s for *T*_1_ and *T*_2_, respectively, were used for the preparation of Figures 3 and 4. The results were intended to be a guide for ion selection or for design purposes, where only the distance needs to be determined.

**Figure 5:**
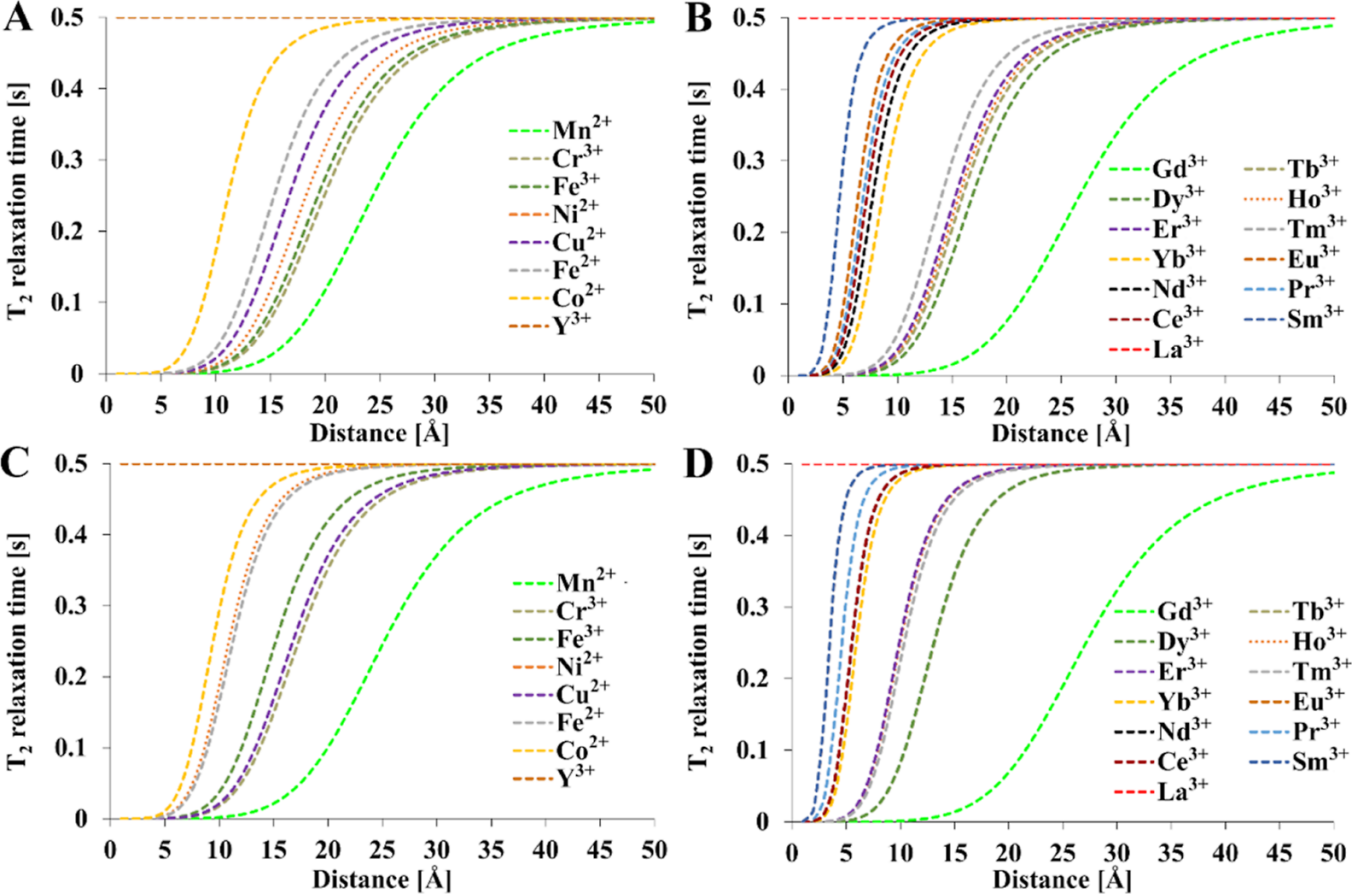
Theoretical relationship between the metal–fluorine nucleus distance and the transverse relaxation time [(A,B)—1 T and (C,D)—9.4 T]. Relaxation times of diamagnetic references: *T*_1_ = 1 s and *T*_2_ = 0.5 s, *T* = 300 K, τ_R_ = 0.25 ns. *T*_1e_ values were calculated from Table 4. Mathematical model encompasses eqs 1–4, 12, and 15.

All metal ions with sufficiently high effective magnetic moments and electronic relaxation times were used for the calculations. Some ions can have high- or low-spin states and different oxidation states that differ in PRE. In the case of iron and manganese, high-spin (HS) Fe^2+^ and HS and low-spin (LS) Mn^2+^, Mn^3+^, and Fe^3+^ are paramagnetic. Both Eu^2+^ and Eu^3+^ are paramagnetic with multiple LS states at room temperature for Eu^3+^ and a single LS state for Eu^2+^.^80^ Similarly, Co^2+^ is paramagnetic in both spin states, whereas Co^3+^ is diamagnetic. Copper has several oxidation states and single-spin states, but only Cu^2+^ is paramagnetic. In Figures 4 and 5, only HS states of Fe^3+^ and Co^2+^ are presented. In the case of Eu^2+^, the results are identical to those of Gd^3+^. Only the results for Gd^3+^ were presented. The results for all spin and oxidation states of Co, Eu, and Fe are presented in Figure S2. The field dependence was considered according to the data from Table 4. The data were presented as relaxation times.

In the case of the relaxation time *T*_1_ and *T*_2_ (Figures 4 and 5), the greatest reduction in relaxation time was observed for the Gd^3+^ ion. Upon assuming a minimal T_2_ relaxation time that enables signal acquisition in a typical MRI/NMR instrument of 10 ms, the complete blanking of the NMR signal occurs at ≈14 Å for Gd^3+^, ≈12.5 Å for Mn^2+^, and ≈8.5 Å for Cu^2+^ in a 9.4 T magnetic field. If 1 ms is assumed to be minimal, then the distances are 9.5 Å for Gd^3+^, 8.5 Å for Mn^2+^, and 6 Å for Cu^2+^. If a minimal *T*_1_ of 10 ms is assumed, the corresponding distances are shorter because of the accompanying line broadening associated with *T*_2_. Changing the magnetic field strength does not significantly affect the point where the signal is blanked. However, the use of a weaker magnetic field can slightly extend the working M–F distance, where the PRE effect is still significant. Even at a relatively large distance from fluorine of 25 Å, a gadolinium ion can halve the relaxation time of ^19^F compared to a diamagnetic compound. In the case of Mn^2+^, this occurs around 23 Å. At such large distances, the remaining paramagnetic ions do not show a significant PRE effect. Most ions exert significant PRE at a maximum distance of 5–6 Å. Samarium shows the weakest effect, where blanking can only be observed at short distances of 2 Å which are unlikely in typical fluoroorganic complexes where the distance is 5 Å or more (Table 2). If the distance of the paramagnetic ion from fluorine nuclei is within 5–15 Å, then the appropriate ion can be selected to fine-tune the desired relaxation rate.

The results indicate that several ions other than Gd^3+^, such as Mn^2+^, Cu^2+^, Cr^3+^, Fe^3+^, Tb^3+^, Dy^3+^, Ho^3+^, Er^3+^, and Tm^3+^, could be used as components of ^19^F contrast agents. However, they have rarely been used in practice. These ions exhibit a large effective magnetic moment (μ_eff_ × μ_B_^–1^ > 7) or long electronic relaxation time (>10^–9^ s). In the case of Gd^3+^ and Eu^2+^, both criteria are met.

Special attention should be paid to copper, manganese, and iron because of their presence in organisms as micronutrients and lower potential toxicity compared with that of exogenous metals. Although chromium and other lanthanoids might be useful, concerns about toxicity would likely limit their potential application.

#### *T*_2_/*T*_1_ Ratio in Paramagnetic Complexes

**Figure 6:**
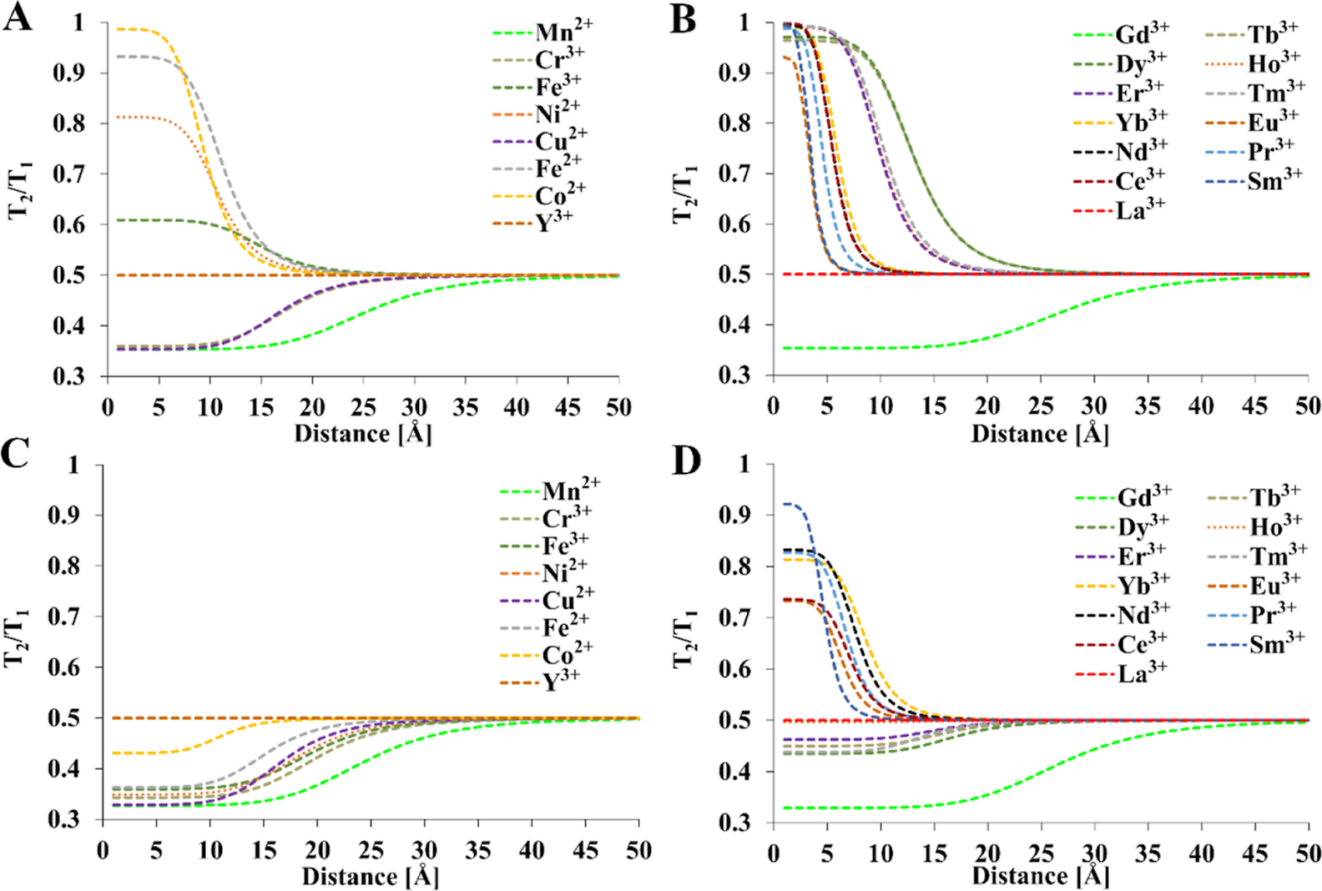
Relationship between the M–F distances in the *T*_2_/*T*_1_ ratio [(A,B)—1 T and (C,D)—9.4 T]. Relaxation times of diamagnetic references: *T*_1_ = 1 s and *T*_2_ = 0.5 s, *T* = 300 K, τ_R_ = 0.25 ns, *a*_HFC_ = 0. *T*_1e_ values were calculated from Table 4. Mathematical model encompasses eqs 1–4, 12, and 15.

To maximize the utility of the PRE effect, an appropriate paramagnetic ion at a carefully selected distance should be used to obtain an appropriate shortening of *T*_1_ relaxation time. An important concern is the excessive broadening of the signal due to the simultaneous shortening of *T*_2_.^27^ In the case of a short *T*_1_ but relatively long *T*_2_, it is possible to maximize the sensitivity of detection, but *T*_2_/*T*_1_ is always less than 1.^27^ Assuming *T*_1_ and *T*_2_ values of 1 and 0.5 s, respectively, for a diamagnetic complex, the relationship between the *T*_2_/*T*_1_ ratio and the M–F distance was calculated for all paramagnetic ions (Figure 6). The lanthanoids and transition metals were presented separately for two field strengths.

The results indicate that the *T*_2_/*T*_1_ ratio either increases or decreases with the M–F distance depending on *B*_0_ and the metal. Only Gd^3+^ exhibits an increase in *T*_2_/*T*_1_ with increasing distance regardless of the external field, whereas the reverse is true for most other metals and fields. Interestingly, there is a strong dependence between the field and the maximal *T*_2_/*T*_1_ ratio. For most d-metals, the ratio is strongly reduced at 9.4 T compared to that in 1 T fields, whereas lanthanoids are less sensitive to the magnetic field. This has strong implications for the design of contrast agents and the translation of these results into medical practice. First, one must investigate the relative suitability of a particular metal if only the *T*_2_/*T*_1_ ratio is considered. For magnetic fields 1 T or slightly greater, the order is constant for lanthanoids and does not depend on the distance. Knowing the order may greatly simplify the design of new agents. However, at strong field strengths (*B*_0_ = 9.4 T), the order of metal ions with respect to the *T*_2_/*T*_1_ ratio strongly depends on the M–F distance, especially within the 5–10 Å range typical for most fluoroorganic ligands. Because most of the research is currently done using 9–11 T NMR instruments or 1–3 T medical scanners, it is difficult to assess the suitability of contrast agents under different field strengths than that of initially used. Another concern is the contact interaction that may greatly decrease the *T*_2_/*T*_1_ ratio in the case of d-metals. This is especially important in the case of Mn^2+^ (Table 3).

#### Paramagnetic, Metal-Based Smart Contrast Agents

Another possible application of the PRE effect is in so-called smart or responsive contrast agents. Most contrast agents of this type use paramagnetic ions to completely turn off the ^19^F MRI signal when the paramagnetic ion is in the proximity of fluorine. As the distance between the paramagnetic ion and the fluorine increases, the ^19^F NMR signal increases. Cleavable linkers are the most popular approach to develop responsive contrast agents. These contrast agents will decompose under certain conditions such as a desired pH range or enzyme presence.^81^ The length of the cleavable linkers typically does not exceed 10 Å, and gadolinium is the most commonly used metal in these contrast agents due to its very strong PRE.^22,82−85^ After the linker is cleaved, the M–F distance increases to 50–1000 Å depending on the concentrations of the agent which is typically in the concentration range of 0.1–10 mM. However, this strategy is limited by the necessity for an internal standard for ^19^F signal quantification to avoid confounding the progress of activation with the concentration of the contrast agent. Redox-sensitive contrast agents can behave in a similar way. In such a case, the PRE effect can be switched on or off by means of metal ion oxidation/reduction which is tantamount to an increase in the distance between the paramagnet and fluorine to infinity if only one oxidation state is paramagnetic. Cu^+/2+^, Mn^2+/3+^, Co^2+/3+^, and Eu^2+/3+^ were proposed as redox-sensitive contrast agents due to their significant PRE and low redox potential achievable under in vivo conditions.^28,29,86−88^ When selecting a paramagnet for a contrast agent containing a cleavable linker, the linker length must be considered. Too long a linker in combination with a weak paramagnet will have a small impact on the image contrast because relaxation times will not significantly change.

**Figure 7:**
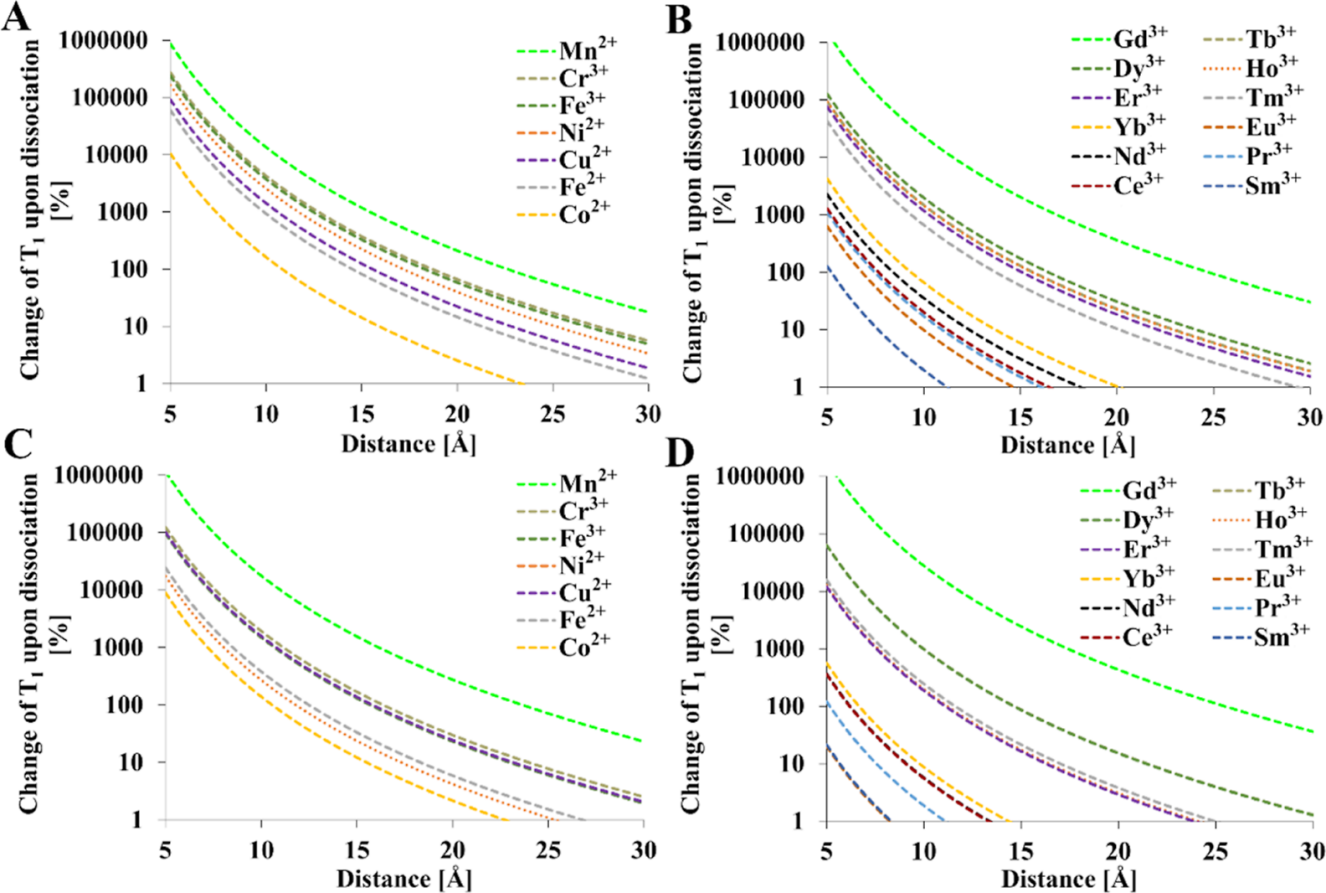
Change in fluorine longitudinal relaxation times during the activation of a hypothetical smart contrast agent with a cleavable linker. The horizontal axis represents the initial distance. The final distance was fixed at 50 Å [(A,B)—1 T and (C,D)—9.4 T]. Relaxation times of diamagnetic references: *T*_1_ = 1 s, *T* = 300 K, τ_R_ = 0.25 ns. *T*_1e_ values were calculated from Table 4. Mathematical model encompasses eqs 1–4, 12, and 15.

**Figure 8:**
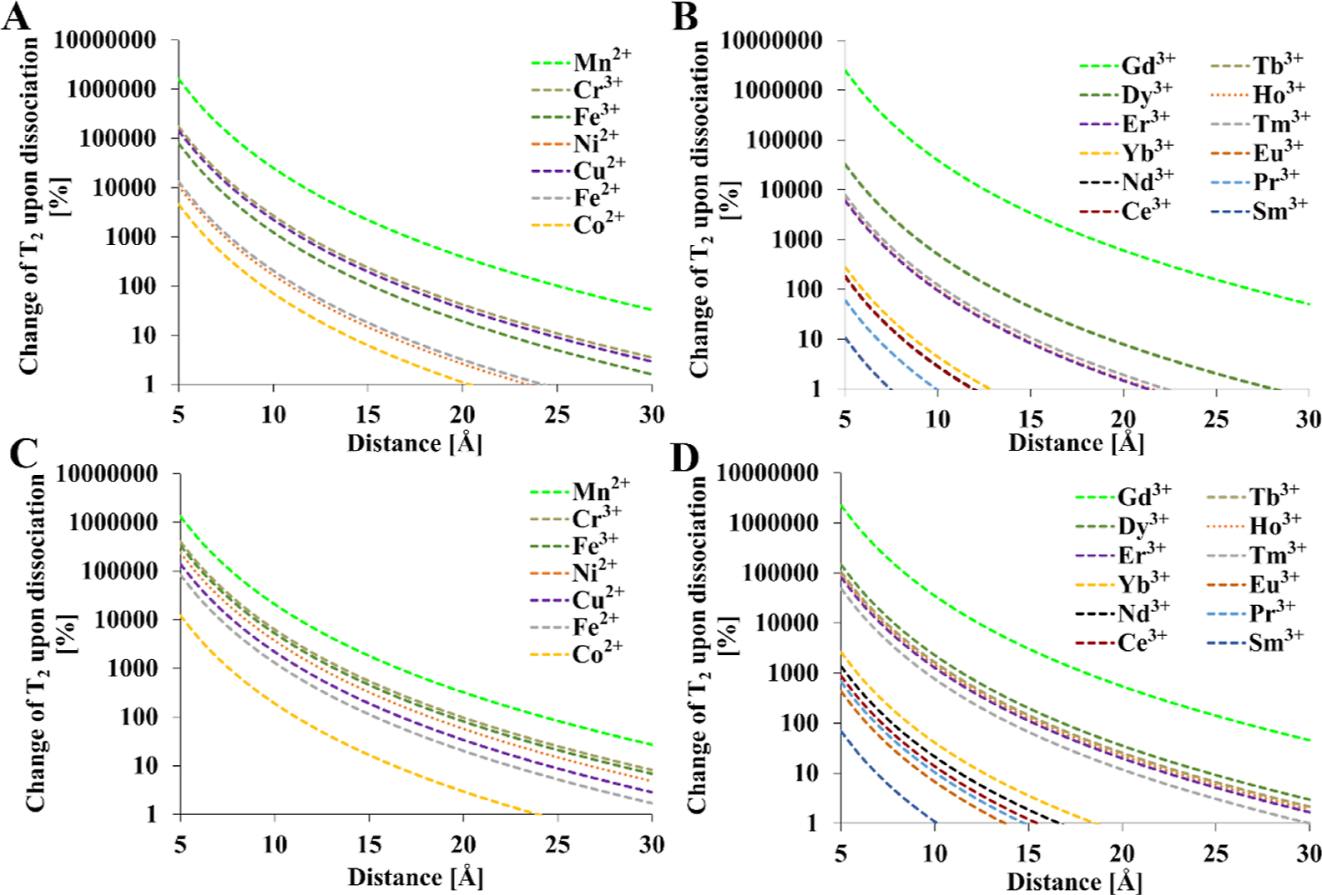
Change in fluorine transverse relaxation times during the activation of a hypothetical smart contrast agent. The horizontal axis represents the initial distance. The final distance was fixed at 50 Å [(A,B)—1 T and (C,D)—9.4 T]. Relaxation times of diamagnetic references: *T*_2_ = 0.5 s, *T* = 300 K, τ_R_ = 0.25 ns. *T*_1e_ values were calculated from Table 4. Mathematical model encompasses eqs 1–4, 12 and 15.

The effect of M–F distance on relaxation times is presented in Figures 7 and 8. Assuming that the distance after a 50 Å linker (≈10 mM solution) is cleaved, most of the paramagnetic ions could be used to turn off the ^19^F MRI signal as long as the initial distance is relatively short (<10 Å). Tb^3+^, Dy^3+^, Ho^3+^, Fe^3+^, Cr^3+^, Co^2+^, and Cu^2+^ provided an ≈1000% increase in relaxation times upon dissociation of a putative smart contrast agent if the initial distance was less than 10 Å. For longer initial distances, only Gd^3+^ and Mn^2+^ can be effectively used. This is reflected in the literature since Gd^3+^ was almost exclusively proposed as a paramagnetic ion for smart contrast agents. However, Mn^2+^ and other transition metals demonstrate potential for use in novel, smart contrast agents.

### Contrast Agents That Utilize the Variation of the Metal–Fluorine Distance as a Result of Isomerization or Conformational Changes

**Figure 9:**
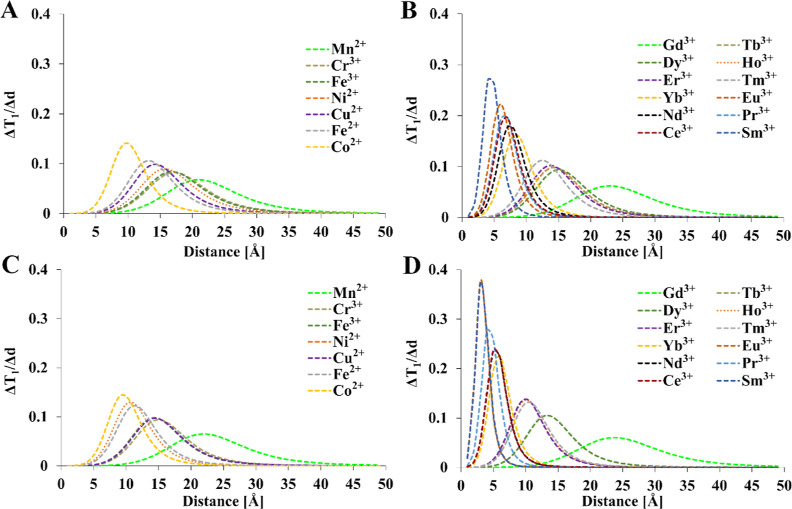
First derivatives of the longitudinal relaxation times from Figure 4 depending on the M–F distance [(A,B)—1 T and (C,D)—9.4 T]. Relaxation times of diamagnetic references: *T*_1_ = 1 s, *T* = 300 K, τ_R_ = 0.25 ns. *T*_1e_ values were calculated from Table 4. Mathematical model encompasses eqs 1–4, 12, and 15.

**Figure 10:**
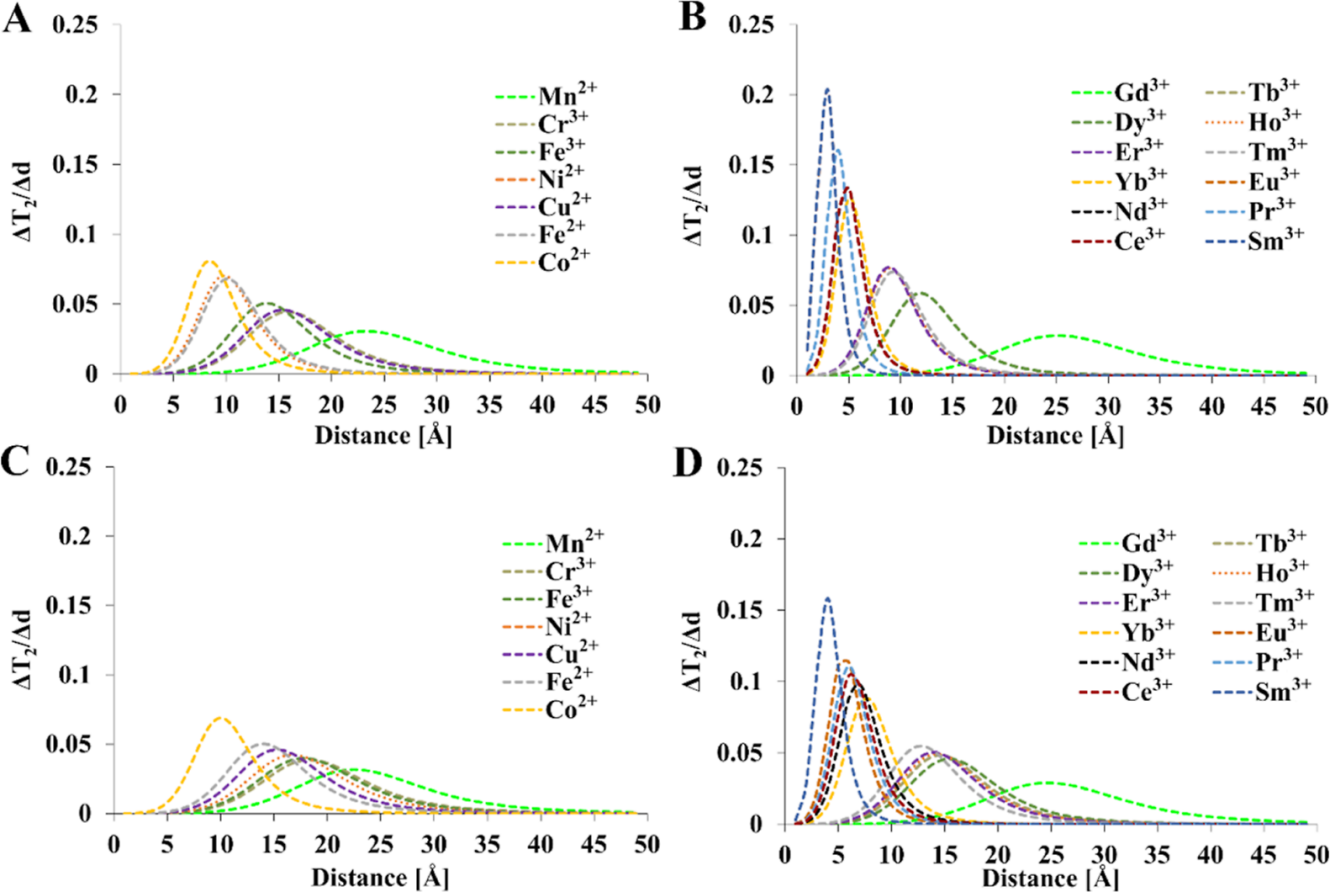
First derivatives of the transverse relaxation times from Figure 5 depending on the M–F distance [(A,B)—1 T and (C,D)—9.4 T]. Relaxation times of diamagnetic references: *T*_2_ = 0.5 s, *T* = 300 K, τ_R_ = 0.25 ns. *T*_1e_ values were calculated from Table 4. Mathematical model encompasses eqs 1–4, 12, and 15.

Another type of smart contrast agent uses non-destructive phenomena leading to more subtle changes in the M–F distance^23,25,89^ An example of such a system is fluoroorganic paramagnetic complexes in which the M–F distance is dependent on the presence of target ions such as Ca^2+^.^90^ A possible design approach could be based on the selection of the rate of the change of the PRE effect within a particular distance range. This is presented in Figures 9 and 10 by calculating the first derivative of Figures 4 and 5. The best ion can be selected based on the average distance between a paramagnetic ion and fluorine atoms in the on and off states.

Generally, the most suitable ions for initial experiments can be selected based on the position of the maxima in Figures 9 and 10. For example, if the observed changes are between 9 and 11 Å, the highest relative changes in relaxation times are observed for Co^2+^, Ni^2+^, Fe^2+^, Er^3+^, and Tm^3+^ at 1 T. In all cases, the potential response is slightly lower at 9.4 T compared to that in 1 T magnetic fields. Alternatively, relaxation rates for a range of metal ions can be calculated for expected distances using data from Table 4 and eqs 1–4, 12, and 15.

#### Redox Contrast Agents

**Figure 11:**
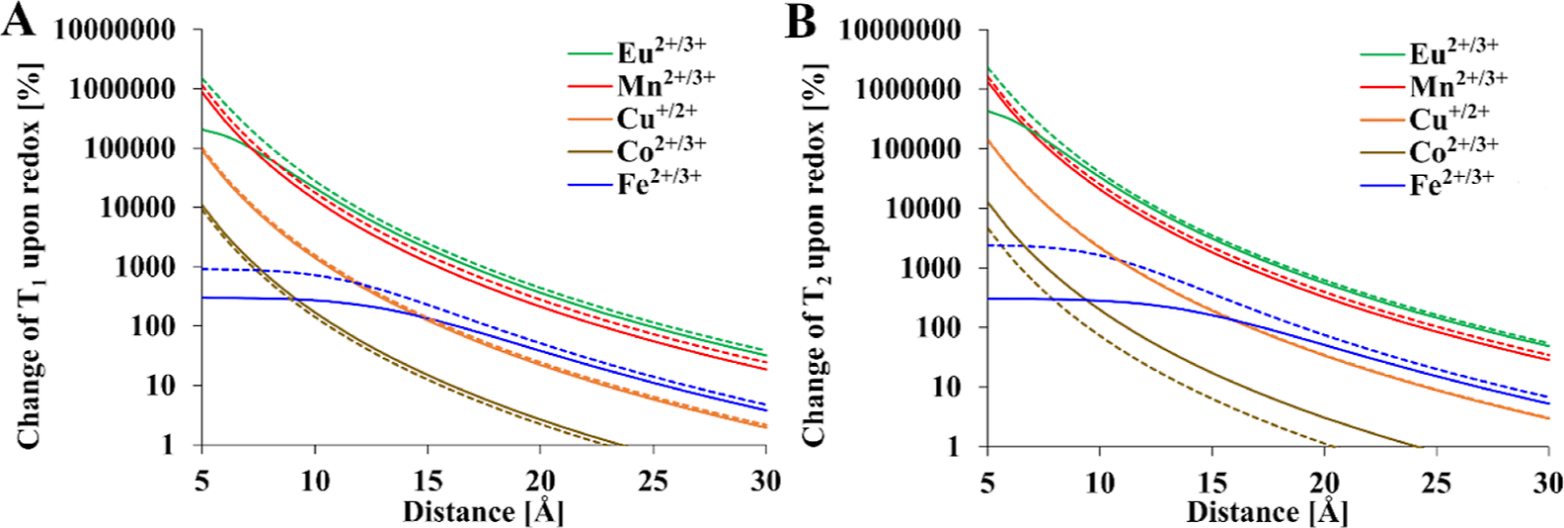
Change in fluorine relaxation times due to the redox state for a particular metal ion in a hypothetical smart contrast agent [(A) *T*_1_, (B) *T*_2_]. Solid line 9.4 T, dashed line 1 T. Relaxation times of diamagnetic references: *T*_1_ = 1 s, *T*_2_ = 0.5 s, *T* = 300 K, τ_R_ = 0.25 ns. Transitions between HS states were considered for all ions. *T*_1e_ values were calculated from Table 4. Mathematical model encompasses eqs 1–4, 12, and 15.

An interesting phenomenon is the change in the magnetic properties of metal ions with a change in the oxidation state. For ^19^F MRI, the most attractive contrast agents are low-molecular weight compounds whose metal ions change their oxidation state in a reducing or oxidizing environment. Consequently, these smart contrast agents can be used to track redox activity in living organisms using MRI.^7,91^ For example, in the pair of Mn^2+^ and Mn^3+^, the former oxidation state exerts much greater PRE effect than the latter.^28,91^ As a result, the ^19^F signal will change due to increased or decreased relaxation rates. In a similar way, other metal ion pairs can be utilized including Eu^2+/3+^,^88^ Mn^2+/3+^,^28^ Co^2+/3+^,^92^ Cu^+/2+^,^93^ and Fe^2+/3+^.^94^ The changes in redox states are also applicable for ^1^H MRI.^95^ The changes in relaxation rates were different for each metal ion, and the calculated effects are presented in Figure 11.

As the M–F distance increases, the results from changing the oxidation state gradually decrease. If the adopted criterion for selecting the appropriate pair is a 30-fold change in relaxation (Figure 11), Eu^2+/3+^ and Mn^2+/3+^ are the most favorable metal ion pairs. Moreover, Eu^2+/3+^ and Mn^2+/3+^ are suitable over a wide range of M–F distances (5–15 Å). In the case of Cu^+/2+^ and Co^2+/3+^, these ranges were shorter (5–10 Å and 5–7 Å for Cu^+/2+^ and Co^2+/3+^, respectively). Changes in relaxation time are more complicated for Fe^2+/3+^ because of the two possible spin states for Fe^2+^ and Fe^3+^. Both HS and LS states of Fe^3+^ are paramagnetic, but the LS state of Fe^2+^ is diamagnetic. For this reason, changes in the relaxation time induced by changes in the oxidation state are smaller and do not exceed 10-fold (Figure 11). The most suitable metal ion pairing for redox contrast agents would be the transition between LS Fe^2+^ (diamagnetic) and HS Fe^3+^ (30-fold change at 10 Å). The impact of the magnetic field is negligible in the case of *T*_1_, but the field strength does have a small effect on *T*_2_ as is evident in the case of the Co^2+/3+^ pair. The actual response may vary if contact relaxation mechanism plays a significant role.

### Spin Cross-Over Agents

**Figure 12:**
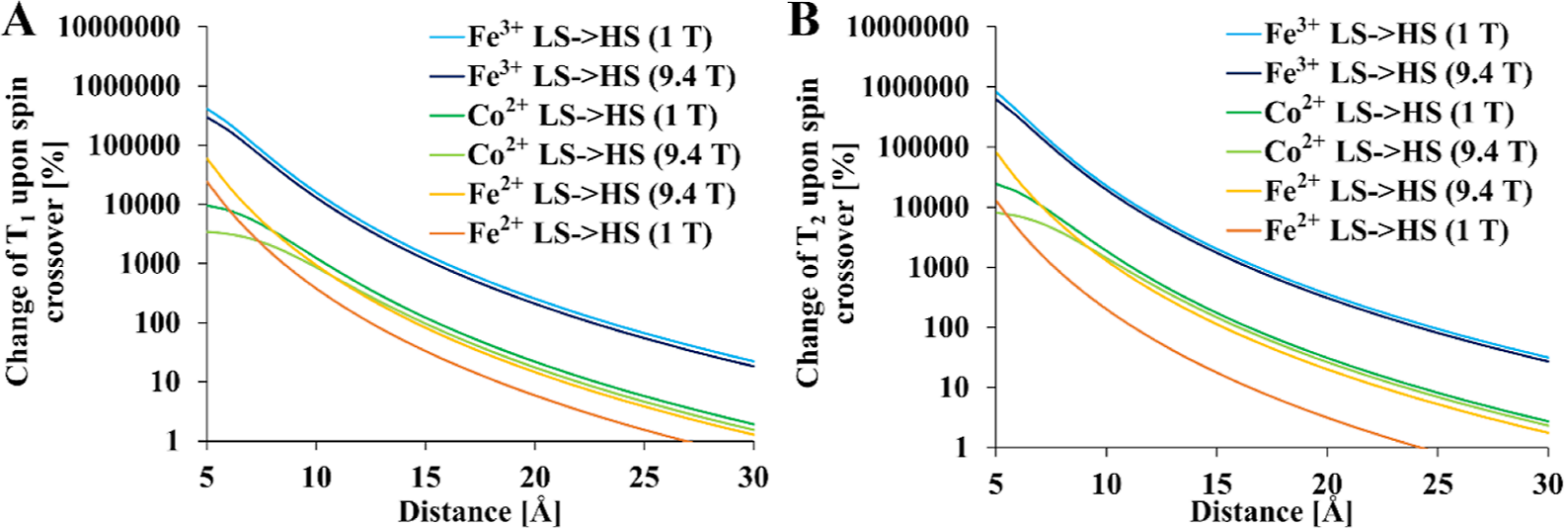
Change in fluorine relaxation times during the spin cross-over effect in a hypothetical smart contrast agent [(A) *T*_1_, (B) *T*_2_]. Relaxation times of diamagnetic references: *T*_1_ = 1 s, *T*_2_ = 0.5 s, *T* = 300 K, τ_R_ = 0.25 ns. *T*_1e_ values were calculated from Table 4. Mathematical model encompasses eqs 1–4, 12, and 15.

Spin cross-over is a transition between the LS and HS states of some metal complexes. This phenomenon may be induced by changes in temperature or pressure or may be caused by radiation.^96^ The possibility of modulating the spin state of a given metal ion theoretically allows one to obtain a new type of relaxometric contrast agent. A single contrast agent based on this mechanism has been described, but the detection was based on a change in the chemical shift and not a change in the relaxation time of fluorine.^97^ Metal ions with potential applications in such a system are Fe^2+^, Fe^3+^, and Co^2+^. The results of the prediction of their relaxation properties are shown in Figure 12.

Fe^3+^ had the greatest effect on relaxation times. Therefore, Fe^3+^ spin cross-over-based contrast agents would be feasible for fluorine–iron distances ranging from 5 to 15 Å regardless of the external field.

## Conclusions

Theoretical calculations of the relaxation rates for fluoroorganic complexes can aid in the design of new ^19^F contrast agents. Knowing the distance between the fluorine and paramagnetic ions is required to predict the extent of the PRE effect. Considering dipolar and Curie relaxation mechanisms leads to the correct predictions (eqs 1–4) of relaxation times. However, in some cases, Fermi contact interaction should also be considered. The predictions are fairly accurate in terms of the order of magnitude of the *T*_1_ and *T*_2_ reduction. These predictions are sufficient estimates for practical purposes, especially in the case of very short relaxation times. The prediction power of the model could be improved if field-dependent relaxation data were available for a wider range of fluoroorganic paramagnetic complexes that would allow more precise determination of τ_s0_ and τ_v_. Furthermore, more precise τ_s0_ and τ_v_ measurements are especially important for the translation of new contrast agents into medical practice where low-field instruments are dominant compared to the high-field strength (9.4–16.1 T) instruments used in research. Such data would also allow for precise calculation of correlation times and M–F distances and a better understanding of the behavior of new contrast agents, especially in terms of the significance of the different relaxation mechanisms. Multiple-field relaxation data would also allow the inclusion of chemical shift anisotropy–anisotropic dipolar shielding cross-correlation in the model with further improvement in accuracy. The presented results offer some general guidance for the selection of paramagnetic ions at the design stage of a contrast agent. Field strength and M–F distance are among the crucial factors. In the case of T_1_, Gd^3+^ shows great potential as a contrast agent because it reduces the relaxation time by several orders of magnitude compared to nonparamagnetic Y^3+^ in a broad range of distances up to 20 Å. However, fluorine relaxation times are extremely short for Gd^3+^ ions when M–F distances are shorter than 10 Å, thus rendering the contrast agent invisible in ^19^F MRI. This phenomenon is convenient for responsive contrast but not in the case of other types of contrast agents. For complexes where the M–F distance is less than 8 Å, the use of other ions, such as Co^2+^ (in the range of 5–7 Å) or Fe^3+^ and Ni^2+^ (in the range of 7–8 Å), is also warranted. Moreover, due to their common occurrence in the body, Co^2+^, Ni^2+^, and Fe^3+^ pose less of a toxicity risk compared to Gd^3+^. Depending on the field strength and the intended application, the suitable ions that enable a sufficient change in relaxation properties compared to diamagnetic compounds are presented in Table 6. Among the transition metals, Mn^2+^ has the greatest potential as a component in various types of contrast agents, which is not yet reflected in the literature. Unfortunately, the presence of significant contact interaction might preclude the use of this metal in some cases. Cr^3+^ demonstrates potential for standard contrast agents where the M–F distance is in the range of 9–11 Å. A considerable limitation of Cr^3+^ is the toxicity concern. Other lanthanoids, particularly Yb^3+^, could be used at short M–F distances of 4–5 Å, regardless of the magnetic field. Lanthanoids such as Ho^3+^, Er^3+^, Dy^3+^, and Tb^3+^ are also potentially useful and should be considered during the development of new paramagnetic contrast agents. However, these metals unfortunately suffer from the same potential side effects as those of Gd^3+^. The utility of paramagnetic ions with a small magnetic moment might be in nanoparticle contrast agents where the distance between ^19^F and the paramagnetic center is much shorter than that in typical fluoroorganic complexes.^98^ Potential design strategies could also take into consideration changes in the chemical shift of ^19^F induced by the paramagnetic ion in order to improve contrast quality.^18,20,27,34^ Dy^3+^ and Tb^3+^ could be the main focus of future research as they create significant ^19^F chemical shifts.

**Table 6:** Suitable^a^ Paramagnetic Ions for Different Types of ^19^F MRI Contrast Agents at Various Field Strengths

	field strength		
type of contrast	1 T	9.4 T	16.5 T
smart with a cleavable linker, initial distance 10 Å, after cleavage 50 Å^b^	Mn^2+^, Gd^3+^	Cr^3+^, Mn^2+^, Fe^3+^, Gd^3+^	Cr^3+^, Mn^2+^, Ni^2+^, Fe^3+^, Tb^3+^, Dy^3+^, Ho^3+^, Gd^3+^
smart of variable distance, initial distance 7 Å, after isomerization 14 Å^b^	Cr^3+^, Mn^2+^, Cu^2+^, Fe^3+^, Dy^3+^, Tb^3+^ Gd^3+^	Cr^3+^, Mn^2+^, Ni^2+^, Fe^2+^, Fe^3+^, Tb^3+^, Er^3+^, Tm^3+^, Dy^3+^, Ho^3+^, Gd^3+^	Cr^3+^, Mn^2+^, Ni^2+^, Fe^2+^, Fe^3+^, Tb^3+^, Er^3+^, Tm^3+^, Dy^3+^, Ho^3+^, Gd^3+^
standard contrast agent, fixed distance 5 Å^c^	Er^3+^, Ho^3+^, Tb^3+^, Dy^3+^, Tm^3+^, Cu^2+^, Ni^2+^, Fe^2+^, Fe^3+^	Er^3+^, Ho^3+^, Tb^3+^, Tm^3+^, Cu^2+^, Co^2+^, Fe^2+^	Tm^3+^, Cu^2+^, Co^2+^
standard contrast agent, fixed distance 10 Å^c^	Gd^3+^, Mn^2+^	Gd^3+^, Mn^2+^	Gd^3+^, Mn^2+^
redox pair^b^	Mn^2+^^/^^3+^(5–15 Å),Cu^+/2+^(5–10 Å),Co^2+/^^3+^(5–7 Å)		
spin cross-over pair^b^	Fe^3+^(5–15 Å),Co^2+^(5–8 Å)		

a The criteria of selection.

b At least 3000% increase in relaxation time due to cleavage or isomerization of a contrast agent.

c Reduction in relaxation time down to 1–10 ms.

## Supporting Information Available

NMR and MS spectra, DFT structural coordinates, additional figures, raw relaxation data, calculated cross-correlation, and rotational correlation time data (PDF)

## References

[ref1] Tirotta I.; Dichiarante V.; Pigliacelli C.; Cavallo G.; Terraneo G.; Bombelli F. B.; Metrangolo P.; Resnati G. 19 F Magnetic Resonance Imaging (MRI): From Design of Materials to Clinical Applications. Chem. Rev. 2015, 115, 1106–1129. 10.1021/cr500286d. 25329814

[ref2] Wermuth P. J.; Jimenez S. A. Nephrogenic Systemic Fibrosis. Scleroderma; Springer US: Boston, MA, 2012; pp 137–159.

[ref3] Morrow J. R.; Tóth É. Next-Generation Magnetic Resonance Imaging Contrast Agents. Inorg. Chem. 2017, 56, 6029–6034. 10.1021/acs.inorgchem.7b01277. 28578587

[ref4] Caschera L.; Lazzara A.; Piergallini L.; Ricci D.; Tuscano B.; Vanzulli A. Contrast Agents in Diagnostic Imaging: Present and Future. Pharmacol. Res. 2016, 110, 65–75. 10.1016/j.phrs.2016.04.023. 27168225

[ref5] Ruiz-Cabello J.; Barnett B. P.; Bottomley P. A.; Bulte J. W. M. Fluorine (19F) MRS and MRI in Biomedicine. NMR Biomed. 2011, 24, 114–129. 10.1002/nbm.1570. 20842758 PMC3051284

[ref6] Pujales-Paradela R.; Savić T.; Esteban-Gómez D.; Angelovski G.; Carniato F.; Botta M.; Platas-Iglesias C. Gadolinium(III)-Based Dual 1 H/19 F Magnetic Resonance Imaging Probes. Chem. Eur J. 2019, 25, 4782–4792. 10.1002/chem.201806192. 30690809

[ref7] Janasik D.; Krawczyk T. 19F MRI Probes for Multimodal Imaging. Chem.—Eur. J. 2022, 28, e202102556 10.1002/chem.202102556. 34705306

[ref8] Anisimov N. V.; Pavlova O. S.; Agafonnikova A. G.; Kosenkov A. V.; Fomina D. V. Multinuclear Applications on 0.5 T Magnetic Resonance Scanner. Appl. Magn. Reson. 2019, 50, 17–27. 10.1007/s00723-018-1081-3.

[ref9] Knight J. C.; Edwards P. G.; Paisey S. J. Fluorinated Contrast Agents for Magnetic Resonance Imaging; a Review of Recent Developments. RSC Adv. 2011, 1, 1415 10.1039/c1ra00627d.

[ref10] Wolters M.; Mohades S. G.; Hackeng T. M.; Post M. J.; Kooi M. E.; Backes W. H. Clinical Perspectives of Hybrid Proton-Fluorine Magnetic Resonance Imaging and Spectroscopy. Invest. Radiol. 2013, 48, 341–350. 10.1097/rli.0b013e318277528c. 23211551

[ref11] Schmid F.; Höltke C.; Parker D.; Faber C. Boosting 19 F MRI-SNR Efficient Detection of Paramagnetic Contrast Agents Using Ultrafast Sequences. Magn. Reson. Med. 2013, 69, 1056–1062. 10.1002/mrm.24341. 22628001

[ref12] Mizukami S.; Takikawa R.; Sugihara F.; Hori Y.; Tochio H.; Wälchli M.; Shirakawa M.; Kikuchi K. Paramagnetic Relaxation-Based 19 F MRI Probe To Detect Protease Activity. J. Am. Chem. Soc. 2008, 130, 794–795. 10.1021/ja077058z. 18154336

[ref13] Kenwright A. M.; Kuprov I.; De Luca E.; Parker D.; Pandya S. U.; Senanayake P. K.; Smith D. G. 19F NMR Based PH Probes: Lanthanide(Iii) Complexes with PH-Sensitive Chemical Shifts. Chem. Commun. 2008, 2514 10.1039/b802838a. 18506228

[ref14] Bar-Shir A.; Yadav N. N.; Gilad A. A.; van Zijl P. C. M.; McMahon M. T.; Bulte J. W. M. Single 19 F Probe for Simultaneous Detection of Multiple Metal Ions Using MiCEST MRI. J. Am. Chem. Soc. 2015, 137, 78–81. 10.1021/ja511313k. 25523816 PMC4304440

[ref15] Fu C.; Herbst S.; Zhang C.; Whittaker A. K. Polymeric 19F MRI Agents Responsive to Reactive Oxygen Species. Polym. Chem. 2017, 8, 4585–4595. 10.1039/c7py00986k.

[ref16] Tsitovich P. B.; Tittiris T. Y.; Cox J. M.; Benedict J. B.; Morrow J. R. Fe(ii) and Co(ii) *N* -Methylated CYCLEN Complexes as ParaSHIFT Agents with Large Temperature Dependent Shifts. Dalton Trans. 2018, 47, 916–924. 10.1039/c7dt03812g. 29260180

[ref17] O’Neill E. S.; Kolanowski J. L.; Bonnitcha P. D.; New E. J. A Cobalt(ii) Complex with Unique ParaSHIFT Responses to Anions. Chem. Commun. 2017, 53, 3571–3574. 10.1039/C7CC00619E. 28288215

[ref18] Harnden A. C.; Parker D.; Rogers N. J. Employing Paramagnetic Shift for Responsive MRI Probes. Coord. Chem. Rev. 2019, 383, 30–42. 10.1016/j.ccr.2018.12.012.

[ref19] Senanayake P. K.; Kenwright A. M.; Parker D.; van der Hoorn S. K. Responsive Fluorinated Lanthanide Probes for 19F Magnetic Resonance Spectroscopy. Chem. Commun. 2007, 2923 10.1039/b705844f. 17622432

[ref20] Chalmers K. H.; De Luca E.; Hogg N. H. â. M.; Kenwright A. M.; Kuprov I.; Parker D.; Botta M.; Wilson J. I.; Blamire A. M. Design Principles and Theory of Paramagnetic Fluorine-Labelled Lanthanide Complexes as Probes for 19 F Magnetic Resonance: A Proof-of-Concept Study. Chem. Eur. J. 2010, 16, 134–148. 10.1002/chem.200902300. 19957317

[ref21] Takaoka Y.; Sakamoto T.; Tsukiji S.; Narazaki M.; Matsuda T.; Tochio H.; Shirakawa M.; Hamachi I. Self-Assembling Nanoprobes That Display off/on 19F Nuclear Magnetic Resonance Signals for Protein Detection and Imaging. Nat. Chem. 2009, 1, 557–561. 10.1038/nchem.365. 21378937

[ref22] Mizukami S.; Takikawa R.; Sugihara F.; Shirakawa M.; Kikuchi K. Dual-Function Probe to Detect Protease Activity for Fluorescence Measurement and 19 F MRI. Angew. Chem., Int. Ed. 2009, 48, 3641–3643. 10.1002/anie.200806328. 19353604

[ref23] Sakamoto T.; Shimizu Y.; Sasaki J.; Hayakawa H.; Fujimoto K. Signal Turn-on Probe for Nucleic Acid Detection Based on 19F Nuclear Magnetic Resonance. Bioorg. Med. Chem. Lett. 2011, 21, 303–306. 10.1016/j.bmcl.2010.11.013. 21123064

[ref24] Nguyen H. V.-T.; Chen Q.; Paletta J. T.; Harvey P.; Jiang Y.; Zhang H.; Boska M. D.; Ottaviani M. F.; Jasanoff A.; Rajca A.; Johnson J. A. Nitroxide-Based Macromolecular Contrast Agents with Unprecedented Transverse Relaxivity and Stability for Magnetic Resonance Imaging of Tumors. ACS Cent. Sci. 2017, 3, 800–811. 10.1021/acscentsci.7b00253. 28776023 PMC5532724

[ref25] Jiang Z.-X.; Feng Y.; Yu Y. B. Fluorinated Paramagnetic Chelates as Potential Multi-Chromic 19F Tracer Agents. Chem. Commun. 2011, 47, 7233 10.1039/c1cc11150g. PMC3279916 21617807

[ref26] Blahut J.; Benda L.; Kotek J.; Pintacuda G.; Hermann P. Paramagnetic Cobalt(II) Complexes with Cyclam Derivatives: Toward 19F MRI Contrast Agents. Inorg. Chem. 2020, 59, 10071 10.1021/acs.inorgchem.0c01216. 32633944

[ref27] Srivastava K.; Weitz E. A.; Peterson K. L.; Marjańska M.; Pierre V. C. Fe- and Ln-DOTAm-F12 Are Effective Paramagnetic Fluorine Contrast Agents for MRI in Water and Blood. Inorg. Chem. 2017, 56, 1546–1557. 10.1021/acs.inorgchem.6b02631. 28094930

[ref28] Chen H.; Tang X.; Gong X.; Chen D.; Li A.; Sun C.; Lin H.; Gao J. Reversible Redox-Responsive 1 H/ 19 F MRI Molecular Probes. Chem. Commun. 2020, 56, 4106–4109. 10.1039/d0cc00778a. 32163087

[ref29] Yu M.; Xie D.; Phan K. P.; Enriquez J. S.; Luci J. J.; Que E. L. A Co II Complex for 19 F MRI-Based Detection of Reactive Oxygen Species. Chem. Commun. 2016, 52, 13885–13888. 10.1039/c6cc08207f. 27841393

[ref30] Chalmers K. H.; Botta M.; Parker D. Strategies to Enhance Signal Intensity with Paramagnetic Fluorine-Labelled Lanthanide Complexes as Probes for 19 F Magnetic Resonance. Dalton Trans. 2011, 40, 904–913. 10.1039/c0dt01232g. 21127807

[ref31] Xiao Y.-D.; Paudel R.; Liu J.; Ma C.; Zhang Z.-S.; Zhou S.-K. MRI Contrast Agents: Classification and Application (Review). Int. J. Mol. Med. 2016, 38, 1319–1326. 10.3892/ijmm.2016.2744. 27666161

[ref32] Funk A. M.; Fries P. H.; Harvey P.; Kenwright A. M.; Parker D. Experimental Measurement and Theoretical Assessment of Fast Lanthanide Electronic Relaxation in Solution with Four Series of Isostructural Complexes. J. Phys. Chem. A 2013, 117, 905–917. 10.1021/jp311273x. 23259577

[ref33] Miao Q.; Nitsche C.; Orton H.; Overhand M.; Otting G.; Ubbink M. Paramagnetic Chemical Probes for Studying Biological Macromolecules. Chem. Rev. 2022, 122, 9571–9642. 10.1021/acs.chemrev.1c00708. 35084831 PMC9136935

[ref34] Harvey P.; Kuprov I.; Parker D. Lanthanide Complexes as Paramagnetic Probes for 19 F Magnetic Resonance. Eur. J. Inorg. Chem. 2012, 2012, 2015–2022. 10.1002/ejic.201100894.

[ref35] Rogers N. J.; Finney K.-L. N. A.; Senanayake P. K.; Parker D. Another Challenge to Paramagnetic Relaxation Theory: A Study of Paramagnetic Proton NMR Relaxation in Closely Related Series of Pyridine-Derivatised Dysprosium Complexes. Phys. Chem. Chem. Phys. 2016, 18, 4370–4375. 10.1039/c5cp06755c. 26792243

[ref36] Funk A. M.; Harvey P.; Finney K.-L. N. A.; Fox M. A.; Kenwright A. M.; Rogers N. J.; Senanayake P. K.; Parker D. Challenging Lanthanide Relaxation Theory: Erbium and Thulium Complexes That Show NMR Relaxation Rates Faster than Dysprosium and Terbium Analogues. Phys. Chem. Chem. Phys. 2015, 17, 16507–16511. 10.1039/c5cp02210j. 26051749

[ref37] Tao J.; Perdew J. P.; Staroverov V. N.; Scuseria G. E. Climbing the Density Functional Ladder: Nonempirical Meta–Generalized Gradient Approximation Designed for Molecules and Solids. Phys. Rev. Lett. 2003, 91, 146401 10.1103/physrevlett.91.146401. 14611541

[ref38] Hanwell M. D.; Curtis D. E.; Lonie D. C.; Vandermeersch T.; Zurek E.; Hutchison G. R.; Vandermeersch T.; Zurek E.; Hutchison G. R. Avogadro: An Advanced Semantic Chemical Editor, Visualization, and Analysis Platform. J. Cheminform. 2012, 4, 17 10.1186/1758-2946-4-17. 22889332 PMC3542060

[ref39] de la Torre J. G.; Huertas M. .; Carrasco B. HYDRONMR: Prediction of NMR Relaxation of Globular Proteins from Atomic-Level Structures and Hydrodynamic Calculations. J. Magn. Reson. 2000, 147, 138–146. 10.1006/jmre.2000.2170. 11042057

[ref40] Junker A. K. R.; Tropiano M.; Faulkner S.; Sørensen T. J. Kinetically Inert Lanthanide Complexes as Reporter Groups for Binding of Potassium by 18-Crown-6. Inorg. Chem. 2016, 55, 12299–12308. 10.1021/acs.inorgchem.6b02063. 27934409

[ref41] Junker A. K. R.; Tropiano M.; Faulkner S.; Sørensen T. J. Kinetically Inert Lanthanide Complexes as Reporter Groups for Binding of Potassium by 18-Crown-6. Inorg. Chem. 2016, 55, 12299–12308. 10.1021/acs.inorgchem.6b02063. 27934409

[ref42] Egorov S. A.; Ishchenko M. A.; Prokopovich Y. V.; Ivanova V. I. Alkylation of 5-Substituted Tetrazoles with Various Alcohols in 1,2-Dichloroethane in the Presence of BF3·Et2O. Russ. J. Org. Chem. 2020, 56, 1196–1203. 10.1134/s107042802007012x.

[ref43] Xie D.; Yu M.; Kadakia R. T.; Que E. L. Magnetic Resonance Activity-Based Sensing Using Paramagnetic Metals. Acc. Chem. Res. 2020, 53, 2–10. 10.1021/acs.accounts.9b00352. 31809009

[ref44] Bertini I.; Luchinat C.; Parigi G.; Ravera E. NMR of Paramagnetic Molecules: Applications to Metallobiomolecules and Modelsetallobiomolecules and Models, 2017.

[ref45] Pujales-Paradela R.; Savić T.; Brandariz I.; Pérez-Lourido P.; Angelovski G.; Esteban-Gómez D.; Platas-Iglesias C. Reinforced Ni(Ii)-Cyclam Derivatives as Dual 1 H/19F MRI Probes. Chem. Commun. 2019, 55, 4115–4118. 10.1039/c9cc01204d. 30888361

[ref46] Yu M.; Bouley B. S.; Xie D.; Que E. L. Highly Fluorinated Metal Complexes as Dual 19 F and PARACEST Imaging Agents. Dalton Trans. 2019, 48, 9337–9341. 10.1039/c9dt01852b. 31168527 PMC6626988

[ref47] Hequet E.; Henoumont C.; Djouana Kenfack V.; Lemaur V.; Lazzaroni R.; Boutry S.; Vander Elst L.; Muller R. N.; Laurent S. Design, Characterization and Molecular Modeling of New Fluorinated Paramagnetic Contrast Agents for Dual 1H/19F MRI. Magnetochemistry 2020, 6, 8 10.3390/magnetochemistry6010008.

[ref48] Chalmers K. H.; Kenwright A. M.; Parker D.; Blamire A. M. 19 F-lanthanide Complexes with Increased Sensitivity for 19 F-MRI: Optimization of the MR Acquisition. Magn. Reson. Med. 2011, 66, 931–936. 10.1002/mrm.22881. 21381109

[ref49] Kadjane P.; Platas-Iglesias C.; Boehm-Sturm P.; Truffault V.; Hagberg G. E.; Hoehn M.; Logothetis N. K.; Angelovski G. Dual-Frequency Calcium-Responsive MRI Agents. Chem. Eur. J. 2014, 20, 7351–7362. 10.1002/chem.201400159. 24796323

[ref50] De Luca E.; Harvey P.; Chalmers K. H.; Mishra A.; Senanayake P. K.; Wilson J. I.; Botta M.; Fekete M.; Blamire A. M.; Parker D. Characterisation and Evaluation of Paramagnetic Fluorine Labelled Glycol Chitosan Conjugates For19F And1H Magnetic Resonance Imaging Topical Issue on Metal-Based MRI Contrast Agents. Guest Editor: Valerie C. Pierre. J. Biol. Inorg Chem. 2014, 19, 215–227. 10.1007/s00775-013-1028-y. 23955558

[ref51] Herynek V.; Martinisková M.; Bobrova Y.; Gálisová A.; Kotek J.; Hermann P.; Koucký F.; Jirák D.; Hájek M. Low-Molecular-Weight Paramagnetic 19F Contrast Agents for Fluorine Magnetic Resonance Imaging. Magma Magn. Reson. Mater. Phys. Biol. Med. 2019, 32, 115–122. 10.1007/s10334-018-0721-9. PMC6514088 30498883

[ref52] Blahut J.; Bernášek K.; Gálisová A.; Herynek V.; Císařová I.; Kotek J.; Lang J.; Matějková S.; Hermann P. Paramagnetic 19 F Relaxation Enhancement in Nickel(II) Complexes of N -Trifluoroethyl Cyclam Derivatives and Cell Labeling for 19 F MRI. Inorg. Chem. 2017, 56, 13337–13348. 10.1021/acs.inorgchem.7b02119. 29048157

[ref53] Cakić N.; Savić T.; Stricker-Shaver J.; Truffault V.; Platas-Iglesias C.; Mirkes C.; Pohmann R.; Scheffler K.; Angelovski G. Paramagnetic Lanthanide Chelates for Multicontrast MRI. Chem. Commun. 2016, 52, 9224–9227. 10.1039/C6CC04011J. 27291157

[ref54] Bertini I.; Luchinat C.; Parigi G.; Ravera E. NMR of Paramagnetic Molecules: Applications to Metallobiomolecules and Modelsetallobiomolecules and Models, 2017.

[ref55] Mudryk Y.; Rogl P.; Paul C.; Berger S.; Bauer E.; Hilscher G.; Godart C.; Nol H. Thermoelectricity of Clathrate I Si and Ge Phases. J. Phys. Condens. Matter 2002, 14, 7991–8004. 10.1088/0953-8984/14/34/318.

[ref56] Blahut J.; Bernášek K.; Gálisová A.; Herynek V.; Císařová I.; Kotek J.; Lang J.; Matějková S.; Hermann P. Paramagnetic 19 F Relaxation Enhancement in Nickel(II) Complexes of N -Trifluoroethyl Cyclam Derivatives and Cell Labeling for 19 F MRI. Inorg. Chem. 2017, 56, 13337–13348. 10.1021/acs.inorgchem.7b02119. 29048157

[ref57] Bloembergen N. Proton Relaxation Times in Paramagnetic Solutions. J. Chem. Phys. 1957, 27, 572–573. 10.1063/1.1743771.

[ref58] Bloembergen N.; Morgan L. O. Proton Relaxation Times in Paramagnetic Solutions. Effects of Electron Spin Relaxation. J. Chem. Phys. 1961, 34, 842–850. 10.1063/1.1731684.

[ref59] Westlund P.-O. A Generalized Solomon-Bloembergen-Morgan Theory for Arbitrary Electron Spin Quantum Number S. Mol. Phys. 1995, 85, 1165–1178. 10.1080/00268979500101741.

[ref60] Suturina E. A.; Mason K.; Geraldes C. F. G. C.; Chilton N. F.; Parker D.; Kuprov I. Lanthanide-Induced Relaxation Anisotropy. Phys. Chem. Chem. Phys. 2018, 20, 17676–17686. 10.1039/c8cp01332b. 29932451

[ref61] Parker D.; Suturina E. A.; Kuprov I.; Chilton N. F. How the Ligand Field in Lanthanide Coordination Complexes Determines Magnetic Susceptibility Anisotropy, Paramagnetic NMR Shift, and Relaxation Behavior. Acc. Chem. Res. 2020, 53, 1520–1534. 10.1021/acs.accounts.0c00275. 32667187 PMC7467575

[ref62] Pintacuda G.; Hohenthanner K.; Otting G.; Müller N. Angular Dependence of Dipole-Dipole-Curie-Spin Cross-Correlation Effects in High-Spin and Low-Spin Paramagnetic Myoglobin**. J. Biomol. NMR 2003, 27, 115–132. 10.1023/a:1024926126239. 12913408

[ref63] Miao Q.; Nitsche C.; Orton H.; Overhand M.; Otting G.; Ubbink M. Paramagnetic Chemical Probes for Studying Biological Macromolecules. Chem. Rev. 2022, 122, 9571–9642. 10.1021/acs.chemrev.1c00708. 35084831 PMC9136935

[ref64] Mäler L.; Mulder F. A. A.; Kowalewski J. Paramagnetic Cross-Correlation Effects in the Longitudinal Proton Relaxation Ofcis-Chloroacrylic Acid in the Presence of Nickel(II) Ions. J. Magn. Reson., Ser. A 1995, 117, 220–227. 10.1006/jmra.1995.0734.

[ref65] Orton H. W.; Kuprov I.; Loh C.-T.; Otting G. Using Paramagnetism to Slow Down Nuclear Relaxation in Protein NMR. J. Phys. Chem. Lett. 2016, 7, 4815–4818. 10.1021/acs.jpclett.6b02417. 27934036

[ref66] Pintacuda G.; Kaikkonen A.; Otting G. Modulation of the Distance Dependence of Paramagnetic Relaxation Enhancements by CSA×DSA Cross-Correlation. J. Magn. Reson. 2004, 171, 233–243. 10.1016/j.jmr.2004.08.019. 15546749

[ref67] Madhu P. K.; Mandal P. K.; Müller N. Cross-Correlation Effects Involving Curie Spin Relaxation in Methyl Groups. J. Magn. Reson. 2002, 155, 29–38. 10.1006/jmre.2001.2496. 11945030

[ref68] Garg S. K.; Ripmeester J. A.; Davidson D. W. Analysis of NMR Line Shapes of Rigid-lattice Multispin Systems. V. 19 F Shielding Tensors of CF 3 Groups Bonded to Carbon. J. Chem. Phys. 1983, 79, 4101–4105. 10.1063/1.446358.

[ref69] Ostfeld D.; Cohen I. A. A Cautionary Note on the Use of the Evans Method for Magnetic Moments. J. Chem. Educ. 1972, 49, 829 10.1021/ed049p829.

[ref70] Saragi T.; Permana B.; Therigan A.; Sinaga H. D.; Maulana T.; Risdiana R. Study of Magnetic Properties and Relaxation Time of Nanoparticle Fe3O4-SiO2. Materials 2022, 15, 1573 10.3390/ma15041573. 35208111 PMC8877505

[ref71] Koenig S. H.; Brown R. D.; Spiller M. The Anomalous Relaxivity of Mn3+(TPPS4). Magn. Reson. Med. 1987, 4, 252–260. 10.1002/mrm.1910040306. 3574059

[ref72] Caravan P.; Ellison J. J.; McMurry T. J.; Lauffer R. B. Gadolinium(III) Chelates as MRI Contrast Agents: Structure, Dynamics, and Applications. Chem. Rev. 1999, 99, 2293–2352. 10.1021/cr980440x. 11749483

[ref73] Viswanathan S.; Kovacs Z.; Green K. N.; Ratnakar S. J.; Sherry A. D. Alternatives to Gadolinium-Based Metal Chelates for Magnetic Resonance Imaging. Chem. Rev. 2010, 110, 2960–3018. 10.1021/cr900284a. 20397688 PMC2874212

[ref74] Fries P. H.; Belorizky E. Electronic Relaxation of Paramagnetic Metal Ions and NMR Relaxivity in Solution: Critical Analysis of Various Approaches and Application to a Gd(III)-Based Contrast Agent. J. Chem. Phys. 2005, 123, 124510 10.1063/1.2011389. 16397947

[ref75] Krishnan V. V.; Cosman M. An Empirical Relationship between Rotational Correlation Time and Solvent Accessible Surface Area. J. Biomol. NMR 1998, 12, 177–182. 10.1023/a:1008226330666. 20700691

[ref76] Koenig S. H. Brownian Motion of an Ellipsoid. A Correction to Perrin’s Results. Biopolymers 1975, 14, 2421–2423. 10.1002/bip.1975.360141115.

[ref77] Tóth É.; Helm L.; Merbach A. E. Relaxivity of MRI Contrast Agents; Springer, 2002, pp 61–101.

[ref78] Dixon A. M.; Larive C. K.; Nantsis E. A.; Carper W. R. Direct Determination of NMR Correlation Times: Analysis of the Cd-CyDTA Complex by the Relaxation Rate Ratio Method. J. Phys. Chem. A 1998, 102, 10573 10.1021/jp9826423.

[ref79] Bain A. D. Chemical Exchange in NMR. Prog. Nucl. Magn. Reson. Spectrosc. 2003, 43, 63–103. 10.1016/j.pnmrs.2003.08.001.

[ref80] Joos J. J.; Smet P. F.; Seijo L.; Barandiarán Z. Insights into the Complexity of the Excited States of Eu-Doped Luminescent Materials. Inorg. Chem. Front. 2020, 7, 871–888. 10.1039/c9qi01455a.

[ref81] Peterson K. L.; Srivastava K.; Pierre V. C. Fluorinated Paramagnetic Complexes: Sensitive and Responsive Probes for Magnetic Resonance Spectroscopy and Imaging. Front. Chem. 2018, 6, 160 10.3389/fchem.2018.00160. 29876342 PMC5974164

[ref82] Mizukami S.; Matsushita H.; Takikawa R.; Sugihara F.; Shirakawa M.; Kikuchi K. 19F MRI Detection of β-Galactosidase Activity for Imaging of Gene Expression. Chem. Sci. 2011, 2, 1151 10.1039/c1sc00071c.

[ref83] Keliris A.; Mamedov I.; Hagberg G. E.; Logothetis N. K.; Scheffler K.; Engelmann J. A Smart19F And1H MRI Probe with Self-Immolative Linker as a Versatile Tool for Detection of Enzymes. Contrast Media Mol. Imaging 2012, 7, 478–483. 10.1002/cmmi.1470. 22821882

[ref84] Hu J.; Wu Q.; Cheng K.; Xie Y.; Li C.; Li Z. A 19F NMR Probe for the Detection of β-Galactosidase: Simple Structure with Low Molecular Weight of 274.2, “Turn-on” Signal without the Background, and Good Performance Applicable in Cancer Cell Line. J. Mater. Chem. B 2017, 5, 4673 10.1039/C7TB00616K. 32264309

[ref85] Li A.; Tang X.; Gong X.; Chen H.; Lin H.; Gao J. A Fluorinated Bihydrazide Conjugate for Activatable Sensing and Imaging of Hypochlorous Acid by 19 F NMR/MRI. Chem. Commun. 2019, 55, 12455–12458. 10.1039/c9cc06622e. 31565704

[ref86] Xie D.; King T. L.; Banerjee A.; Kohli V.; Que E. L. Exploiting Copper Redox For19F Magnetic Resonance-Based Detection of Cellular Hypoxia. J. Am. Chem. Soc. 2016, 138, 2937–2940. 10.1021/jacs.5b13215. 26906216

[ref87] Xie D.; Kim S.; Kohli V.; Banerjee A.; Yu M.; Enriquez J. S.; Luci J. J.; Que E. L. Hypoxia-Responsive19F MRI Probes with Improved Redox Properties and Biocompatibility. Inorg. Chem. 2017, 56, 6429 10.1021/acs.inorgchem.7b00500. 28537705

[ref88] Basal L. A.; Bailey M. D.; Romero J.; Ali M. M.; Kurenbekova L.; Yustein J.; Pautler R. G.; Allen M. J. Fluorinated Eu II -Based Multimodal Contrast Agent for Temperature- and Redox-Responsive Magnetic Resonance Imaging. Chem. Sci. 2017, 8, 8345–8350. 10.1039/c7sc03142d. 29780447 PMC5933353

[ref89] Gambino G.; Gambino T.; Pohmann R.; Angelovski G. A Ratiometric 19F MR-Based Method for the Quantification of Ca2+ Using Responsive Paramagnetic Probes. Chem. Commun. 2020, 56, 3492 10.1039/C9CC09977H. 32129333

[ref90] Harvey P.; Chalmers K. H.; De Luca E.; Mishra A.; Parker D. Paramagnetic 19F Chemical Shift Probes That Respond Selectively to Calcium or Citrate Levels and Signal Ester Hydrolysis. Chem. Eur. J. 2012, 18, 8748–8757. 10.1002/chem.201200737. 22689478

[ref91] Pinto S. M.; Tomé V.; Calvete M. J. F.; Castro M. M. C. A.; Tóth É.; Geraldes C. F. G. C. Metal-Based Redox-Responsive MRI Contrast Agents. Coord. Chem. Rev. 2019, 390, 1–31. 10.1016/j.ccr.2019.03.014.

[ref92] Yu M.; Bouley B. S.; Xie D.; Enriquez J. S.; Que E. L. 19 F PARASHIFT Probes for Magnetic Resonance Detection of H 2 O 2 and Peroxidase Activity. J. Am. Chem. Soc. 2018, 140, 10546–10552. 10.1021/jacs.8b05685. 30052043

[ref93] Kadakia R. T.; Xie D.; Martinez D.; Yu M.; Que E. L. A Dual-Responsive Probe for Detecting Cellular Hypoxia Using 19 F Magnetic Resonance and Fluorescence. Chem. Commun. 2019, 55, 8860–8863. 10.1039/c9cc00375d. PMC6650319 31219109

[ref94] Tanaka K.; Kitamura N.; Takahashi Y.; Chujo Y. Reversible Signal Regulation System of 19F NMR by Redox Reactions Using a Metal Complex as a Switching Module. Bioorg. Med. Chem. 2009, 17, 3818–3823. 10.1016/j.bmc.2009.04.039. 19423355

[ref95] Gale E. M.; Jones C. M.; Ramsay I.; Farrar C. T.; Caravan P. A Janus Chelator Enables Biochemically Responsive MRI Contrast with Exceptional Dynamic Range. J. Am. Chem. Soc. 2016, 138, 15861–15864. 10.1021/jacs.6b10898. 27960350 PMC5328420

[ref96] Kahn O. Spin-Crossover Molecular Materials. Curr. Opin. Solid State Mater. Sci. 1996, 1, 547–554. 10.1016/s1359-0286(96)80070-2.

[ref97] Thorarinsdottir A. E.; Gaudette A. I.; Harris T. D. Spin-Crossover and High-Spin Iron(Ii) Complexes as Chemical Shift19F Magnetic Resonance Thermometers. Chem. Sci. 2017, 8, 2448–2456. 10.1039/c6sc04287b. 28694955 PMC5477811

[ref98] Mashiach R.; Cohen D.; Avram L.; Harris T.; Pinkas I.; Houben L.; Allouche-Arnon H.; Bar-Shir A. Inducing Defects in 19 F-Nanocrystals Provides Paramagnetic-Free Relaxation Enhancement for Improved In Vivo Hotspot MRI. Nano Lett. 2020, 20, 7207–7212. 10.1021/acs.nanolett.0c02549. 32897716 PMC7564093

