# Supplementary material for: Design Principles of Responsive Relaxometric 19F Contrast Agents: Evaluation from the Point of View of Relaxation Theory and Experimental Data
Source: Inorg Chem. 2022 Nov 16;61(48):19524–42. doi: 10.1021/acs.inorgchem.2c03451 (PMC9727736; doi:10.1021/acs.inorgchem.2c03451)
Supplement: Supplementary file 1 — ic2c03451_si_001.pdf [file ic2c03451_si_001.pdf]

Supporting Information

DESIGN PRINCIPLES OF RESPONSIVE RELAXOMETRIC ^{19}F CONTRAST AGENTS. EVALUATION FROM THE POINT OF VIEW OF RELAXATION THEORY AND EXPERIMENTAL DATA

*Mariusz Zalewski, Dawid Janasik, Adrianna Wierzbicka, Tomasz Krawczyk**

Department of Chemical Organic Technology and Petrochemistry, Faculty of Chemistry, Silesian University of Technology, Krzywoustego 4, 44-100 Gliwice

*Tomasz.Krawczyk@polsl.pl

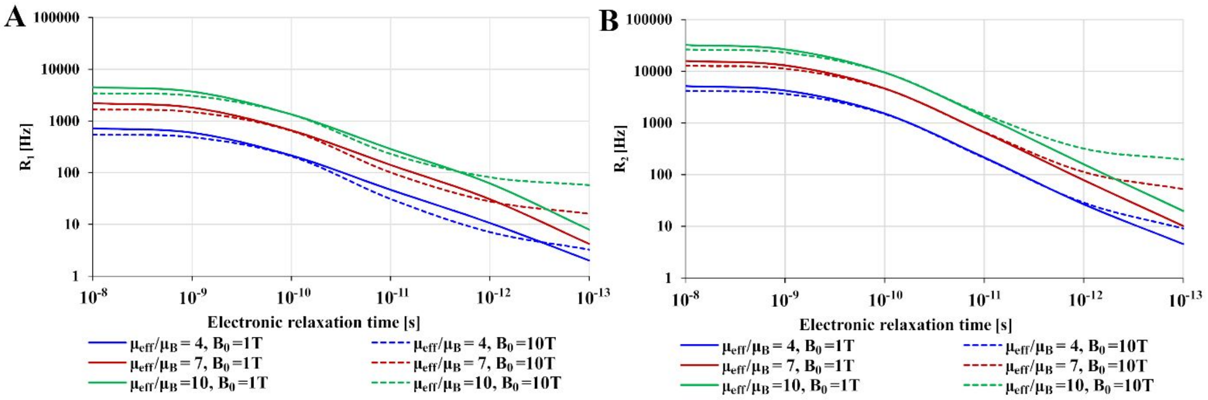

Figure S1. Theoretical relationship between observed relaxation rate (**A** = R_1, **B** = R_2) and electronic relaxation time for ions of high, medium and low effective magnetic moment at different magnetic fields. τ_R = 0.25 ns, $T_{1diamagnetic}$=1 s, $T_{2diamagnetic}$ = 0.5 s.

The loss function used in least squares fitting had the form:

$$loss\ function = (logR_{1(\mathrm{exp.})} - logR_{1(calc.)})^2 + (logR_{2(\mathrm{exp.})} - logR_{2(calc.)})^2 \quad \text{(S1)}$$

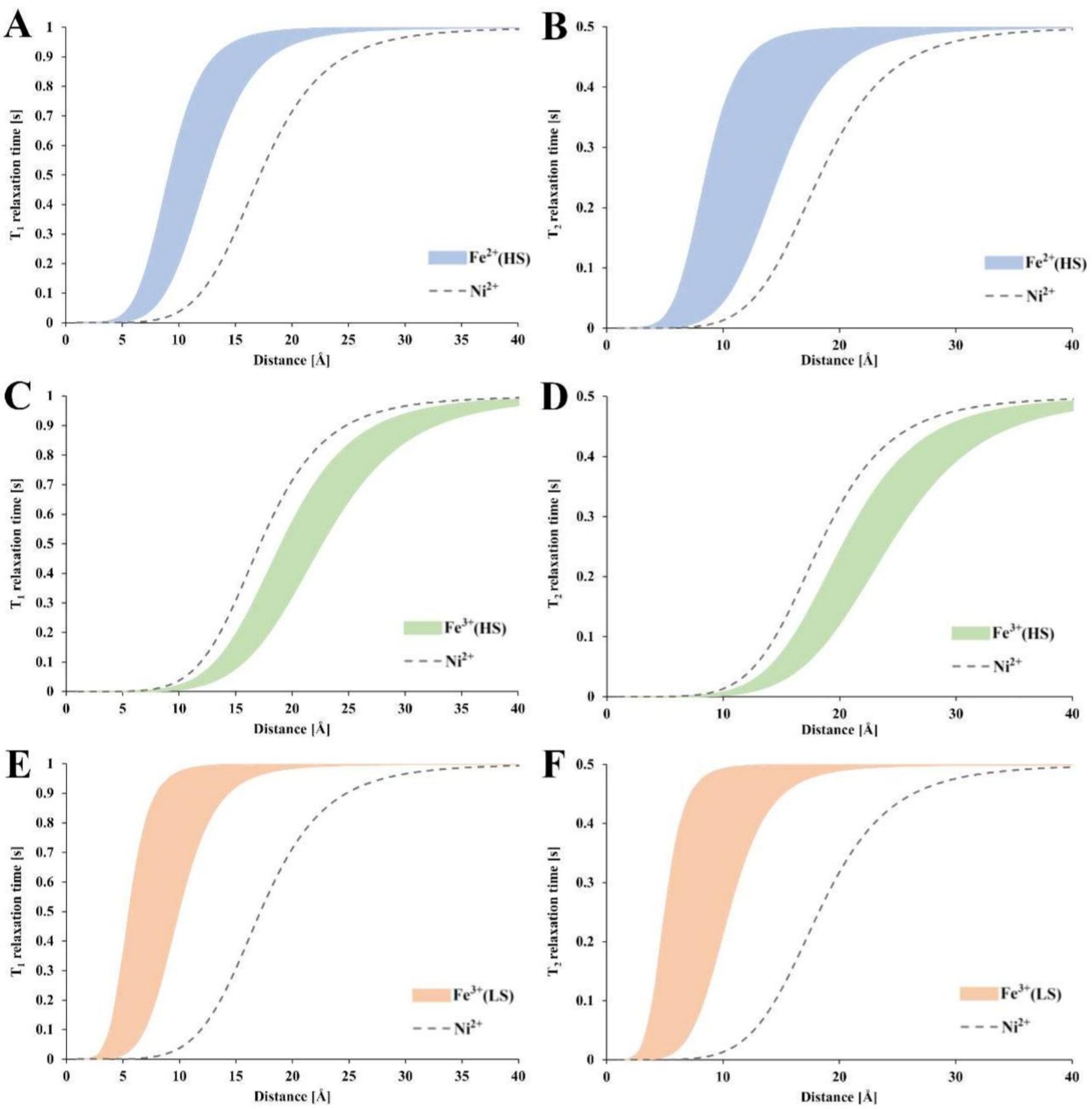

Figure S2. Theoretical relationship between the Fe^{3+} and Fe^{2+}-fluorine nucleus distance and the relaxation time T_1 (**A, C, E**) or T_2 (**B, D, F**). Relaxation times of diamagnetic references: T_1 =1 s and T_2 =0.5 s, B_0= 9.4 T, T= 300 K, τ_R= 0.25 ns. The reported range of electronic relaxation times and magnetic moments were used: **A, B** – $\mu_{eff}\cdot\mu_B^{-1}$= 5.1-5.7, T_e= $1\cdot10^{-11}$-10^{-13} s. **C, D** – $\mu_{eff}\cdot\mu_B^{-1}$= 5.6-6.1, T_e= $1\cdot10^{-9}$-10^{-10} s. **E, F** – $\mu_{eff}\cdot\mu_B^{-1}$= 1.8-2.1, T_e= $1\cdot10^{-11}$-10^{-13} s.

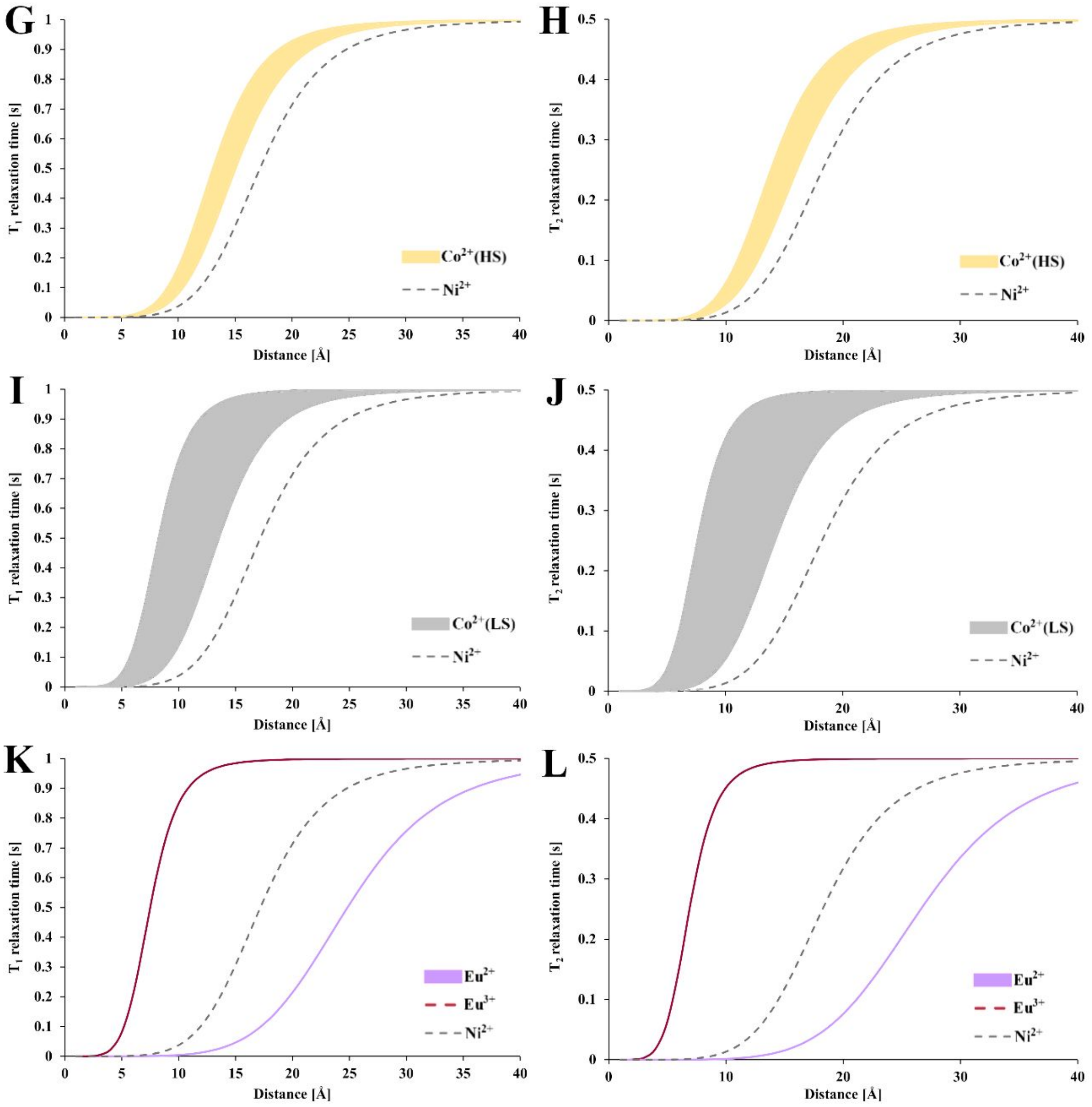

Figure S2 cont. Theoretical relationship between the cobalt ion-fluorine nucleus distance and the relaxation time T_1 (**G, I**) or T_2 (**H, J**). Relaxation times of diamagnetic references: T_1 =1 s and T_2 =0.5 s, B_0= 9.4 T, T= 300 K, τ_R= 0.25 ns. The reported range of electronic relaxation times and magnetic moments were used: **G, H** – $\mu_{eff}\cdot\mu_B^{-1}$= 1.8, T_e= $1\cdot10^{-9}$-10^{-10} s. **I, J** – $\mu_{eff}\cdot\mu_B^{-1}$= 4.2-5.2, T_e= $1\cdot10^{-11}$-10^{-13} s. **K, L** – Eu^{2+}: $\mu_{eff}\cdot\mu_B^{-1}$= 7.63-7.93, T_e= $1\cdot10^{-8}$ s.

Table S1. Differences between the rotational correlation times calculated by HYDRONMR, Stokes-Einstein-Debye equation, and the calculations based on the BRW theoretical equations.

Designation in the original publication	hydrodynamic calculations		BRW calculations*	
	τ_R (Hydro) [ns]	τ_R (Stokes-Einstein-Debye) [ns]	Distance [Å]	τ_R [ns]
**[HL¹]**[44]	0.43	0.32	7.430±0.006	0.25±0.01**
**[HL²]**[44]	0.33	0.28	8.92±0.01	0.25±0.01**
**[L¹]**[20]	0.61	0.52	6.51±0.01	0.284±0.006
**[H_3L^2]**[20]	0.40	0.30	7.02±0.02	0.31±0.02
**[H_3L^{6a}]**[20]	0.40	0.34	5.97±0.02	0.18±0.02
**[H_3L^7]**[20]	0.39	0.46	6.88±0.02	0.32±0.02
**[L^{2b}]**[30]	0.41	0.32	6.62±0.01	0.20±0.01
**[FC]**[25]	0.56	0.41	9.51±0.06	0.101±0.008
**DOTP-tfe**[50]	0.35	0.49	6.3±0.1	0.25±0.01**
**DOTAm-F_{12}**[27]	0.45	0.38	6.21±0.03	0.25±0.01**
**[L^a]**[52]	0.72	0.52	9.0±0.1	0.10±0.02
**[L^b]**[45]	0.64	0.57	7.30±0.03	0.25±0.01**
**[L^c]**[48]	-	-	10.2±0.2	0.4±0.1
**7**[46]	-	-	13.23±0.04	0.29±0.02
**H_2te2f2a** [55]	0.28	0.25	5.24±0.01	0.25±0.01**
**te2f2ae**[55]	0.28	0.26	4.93±0.01	0.250±0.002**
**te2f2py**[55]	0.36	0.31	5.31±0.03	0.25±0.01**
**te2f**[55]	0.31	0.22	5.66±0.03	0.25±0.01**
**H_4te2f2p**[55]	0.29	0.27	4.98±0.03	0.25±0.01**
**[L^2]**[49]	0.73	0.43	6.82±0.01	0.83±0.01
**[L^2-chitosan]**[49]	-	-	6.48±0.05	0.38±0.3
**[L^3]**[49]	0.60	0.47	6.63±0.01	0.53±0.01
**[L^3-chitosan]**[49]	-	-	6.51±0.05	0.20±0.01
**[L^{1a}]** [30]	0.46	0.38	7.02±0.02	0.268±0.01
**H_4te2f2p**[26]	0.38	0.29	5.247±0.004	0.25±0.01**
**H_2te2f2a**[26]	0.38	0.28	5.262±0.002	0.25±0.01**
**[H_3L^1] (orto)**[19]	0.43	0.32	7.265±0.001	0.25±0.01**
**[H_3L^1] (meta)**[19]	0.43	0.32	9.781±0.03	0.25±0.01**
**L^{11}**	0.36	0.28	8.2±0.1	0.19±0.03
**L^{12}**	0.41	0.30	8.44±0.03	0.25±0.01**

* uncertainties correspond to the maximum change in the loss function of 1% resulted by the change of $\boldsymbol{\tau_R}$ or **d.**

** assumed τ_R due to lack of relaxation data

<u>ESI-MS Analysis of L^{11} and L^{12}complexes:</u>

L^{11}:

GdL^{11} – ESI-MS: m/z calculated dla $C_{22}H_{28}N_4O_6F_3{}^{1158}GdNa^+$ $[M+Na]^+$ 682.1105, found 682.1100.

CuL^{11}– ESI-MS: m/z calculated dla $C_{22}H_{28}N_4O_6F_3{}^{63}CuNa_2{}^+$ $[M+2Na]^+$ 610.1052, found 610.1045.

CoL^{11} – ESI-MS: m/z calculated dla $C_{22}H_{29}N_4O_6F_3{}^{59}CoNa^+$ $[M+Na]^+$ 584.1269, found 584.1269.

NiL^{11} – ESI-MS: m/z calculated dla $C_{22}H_{28}N_4O_6F_3{}^{58}NiNa_2{}^+$ $[M+2Na]^+$ 605.1110, found 605.1111.

Fe(III)L^{11} – ESI-MS: m/z calculated dla $C_{22}H_{28}N_4O_6F_3{}^{56}FeNa^+$ $[M+Na]^+$ 580.1208, found 580.1205.

Fe(II)L^{11} – ESI-MS: m/z calculated dla $C_{22}H_{28}N_4O_6F_3{}^{56}FeNa_2{}^+$ $[M+2Na]^+$ 603.1106, found 603.1119.

HoL^{11} – ESI-MS: m/z calculated dla $C_{22}H_{28}N_4O_6F_3{}^{1165}HoNa^+$ $[M+Na]^+$ 689.1162, found 689.1149.

TbL^{11} – ESI-MS: m/z calculated dla $C_{22}H_{28}N_4O_6F_3{}^{1159}TbNa^+$ $[M+Na]^+$ 683.1112, found 683.1120.

DyL^{11} – ESI-MS: m/z calculated dla $C_{22}H_{28}N_4O_6F_3{}^{1163}DyNa^+$ $[M+Na]^+$ 687.1149, found 687.1147.

PrL^{11}– ESI-MS: m/z calculated dla $C_{22}H_{28}N_4O_6F_3{}^{11411}PrNa^+$ $[M+Na]^+$ 665.0937, found 665.0917.

NdL^{11} – ESI-MS: m/z calculated dla $C_{22}H_{28}N_4O_6F_3{}^{114112}NdNa^+$ $[M+Na]^+$ 666.0934, found 666.0920.

MnL^{11}– ESI-MS: m/z calculated dla $C_{22}H_{28}N_4O_6F_3{}^{55}MnNa_2{}^+$ $[M+2Na]^+$ 618.1086, found 618.1073.

YL^{11} – ESI-MS: m/z calculated dla $C_{22}H_{28}N_4O_6F_3{}^{89}YNa^+$ $[M+Na]^+$ 613.0917, found 613.0912.

EuL^{11} – ESI-MS: m/z calculated dla $C_{22}H_{28}N_4O_6F_3{}^{1153}EuNa^+$ $[M+Na]^+$ 677.1072, found 677.1067

ErL^{11} – ESI-MS: m/z calculated dla $C_{22}H_{28}N_4O_6F_3{}^{1168}ErNa^+$ $[M+Na]^+$ 692.1188, found 692.1180.

CeL^{11} – ESI-MS: m/z calculated dla $C_{22}H_{28}N_4O_6F_3{}^{1140}CeNa^+$ $[M+Na]^+$ 664.0913, found 664.0900.

SmL^{11} – ESI-MS: m/z calculated dla $C_{22}H_{28}N_4O_6F_3{}^{115112}SmNa^+$ $[M+Na]^+$ 654.1237, found 654.1225.

CrL^{11}– Assessment based on disappearance of substrate - no visible MS signal.

L^{12}:

GdL^{12} – ESI-MS: m/z calculated dla $C_{23}H_{29}N_5O_7F_3{}^{1158}GdNa^+$ $[M+Na]^+$ 725.1163, found 725.1151.

CuL^{12}– ESI-MS: m/z calculated dla $C_{23}H_{29}N_5O_7F_3{}^{63}CuNa_2{}^+$ $[M+2Na]^+$ 653.1110, found 653.1106.

NiL^{12} – ESI-MS: m/z calculated dla $C_{23}H_{30}N_5O_7F_3{}^{58}NiNa^+$ $[M+Na]^+$ 626.1348, found 626.1334.

Fe(III)L^{12} – ESI-MS: m/z calculated dla $C_{23}H_{29}N_5O_7F_3{}^{56}FeNa^+$ $[M+Na]^+$ 623.1266, found 623.1262.

HoL^{12} – ESI-MS: m/z calculated dla $C_{23}H_{29}N_5O_7F_3{}^{1165}HoNa^+$ $[M+Na]^+$ 732.1220, found 732.1216.

YL^{12} – ESI-MS: m/z calculated dla $C_{23}H_{29}N_5O_7F_3{}^{89}YNa^+$ $[M+Na]^+$ 656.0975, found 656.0969.

CoL^{12}– Assessment based on disappearance of substrate - no visible MS signal.

CrL^{12}– Assessment based on disappearance of substrate - no visible MS signal.

Table S2. Relaxation rates of L^{11} (this work) and L^{12} (this work) complexes (T=298 K, B_0=9.4 T).

Complex	R_1 [Hz]	R_2 [Hz]	Chemical shift [ppm]	Signal width [ppm]
**L^{11}**	0.89±0.04	11±0.5	-62.20	0.13
**YL^{11}**	1.08±0.05	19±1	-62.17	0.14
**GdL^{11}**	597±30	1087±54	-62.14	1.14
**CuL^{11}**	74.9±3.8	108±6	-62.17	0.14
**ErL^{11}**	26.5±1.3	384±19	-65.83	0.75
**CoL^{11}**	16.4±0.8	29±1.5	-63.29	0.14
**Fe(II)L^{11}**	20.4±1	40±2	-60.66	0.12
**Fe(III)L^{11}**	64.9±3.3	137±7	-62.17	0.13
**NiL^{11}**	52.9±2.6	113±6	-62.57	0.15
**HoL^{11}**	39.9±2	92±4.5	-67.35	0.67
**CrL^{11}**	201±10	298±15	-62.85	0.51
**MnL^{11}**	324±16	510±26	-62.94	0.92
**DyL^{11}**	27.3±1.4	58±2.9	-62.11	0.22
**NdL^{11}**	1.72±0.09	51±2.5	-62.87	0.15
**EuL^{11}**	1.32±0.07	9.4±0.5	-61.37	0.16
**PrL^{11}**	1.50±0.08	92±5	-64.03	0.16
**YbL^{11}**	2.91±0.15	-	-60.61	0.20
**CeL^{11}**	1.38±0.07	42±2.1	-62.97	0.15
**SmL^{11}**	1.40±0.07	34±1.7	-62.35	0.12
**YL^{12}**	1.27±0.06	7.3±0.4	-62.49	0.13
**GdL^{12}**	412±21	862±43	-62.52	1.06
**CuL^{12}**	39.4±2	126±6	-62.18	0.10
**ErL^{12}**	27.7±1.4	106±6	-58.99	0.08
**CoL^{12}**	6.96±0.34	18±1	-62.82	0.03
**Fe(III)L^{12}**	181±9	215±11	-62.29	0.15
**NiL^{12}**	35.7±1.8	55±3	-62.33	0.11
**HoL^{12}**	43.5±2.2	92±5	-68.35	0.38
**CrL^{12}**	141±7	277±14	-62.81	0.55
**MnL^{12}**	312±16	1239±62	-62.16	0.63

Chemical shift anisotropy-anisotropic dipolar shielding cross correlation (CSA×DSA)[65]

$$R_1^{CSA \times DSA} = \frac{1}{4\pi}\left[2\xi_{CSA}\xi_{DSA}\frac{3(cos\theta^{CSA,DSA})^2 - 1}{2}\right]\left(\frac{\tau_R}{1 + (\omega_F\tau_R)^2}\right) \quad \text{(S2)}$$

$$R_2^{CSA \times DSA} = \frac{1}{24\pi}\left[2\xi_{CSA}\xi_{DSA}\frac{3(cos\theta^{CSA,DSA})^2 - 1}{2}\right]\left(4\tau_R + \frac{3\tau_R}{1 + (\omega_F\tau_R)^2}\right) \quad \text{(S3)}$$

$$\xi_{CSA} = \sqrt{\frac{8\pi}{15}}\gamma_F B_0 \Delta_{\sigma_{CF3}^{CSA}} \quad \text{(S4)}$$

$$\xi_{DSA} = \sqrt{\frac{8\pi}{15}}\left(\frac{\mu_0}{4\pi}\right)\frac{\gamma_F B_0 \mu_B^2 g_e^2 S(S+1)}{r^3 kT} \quad \text{(S5)}$$

Table S3. Predicted longitudinal relaxation rates [Hz] arising from CSA×DSA cross correlation. B_0 = 9.4 T, τ_r = 0.25 ns, $\Delta_{\sigma_{CF3}^{CSA}} = 100\ ppm$,[68] T = 300 K.

ion	$\theta^{CSA,DSA}$ = 0°			$\theta^{CSA,DSA}$ = 30°			$\theta^{CSA,DSA}$ = 60°			$\theta^{CSA,DSA}$ = 90°		
	**10 Å**	**8 Å**	**6 Å**	**10 Å**	**8 Å**	**6 Å**	**10 Å**	**8 Å**	**6 Å**	**10 Å**	**8 Å**	**6 Å**
**Ce^{3+}**	1.4	1.8	2.4	0.9	1.1	1.5	-0.2	-0.2	-0.3	-0.7	-0.9	-1.2
**Pr^{3+}**	2.9	3.6	4.8	1.8	2.3	3.0	-0.4	-0.5	-0.6	-1.4	-1.8	-2.4
**Nd^{3+}**	2.9	3.7	4.9	1.8	2.3	3.1	-0.4	-0.5	-0.6	-1.5	-1.8	-2.4
**Sm^{3+}**	0.2	0.2	0.3	0.1	0.1	0.2	0.0	0.0	0.0	-0.1	-0.1	-0.1
**Eu^{3+}**	1.5	1.9	2.6	1.0	1.2	1.6	-0.2	-0.2	-0.3	-0.8	-1.0	-1.3
**Tb^{3+}**	21.3	26.6	35.5	13.3	16.6	22.2	-2.7	-3.3	-4.4	-10.6	-13.3	-17.7
**Dy^{3+}**	25.5	31.9	42.6	16.0	20.0	26.6	-3.2	-4.0	-5.3	-12.8	-16.0	-21.3
**Ho^{3+}**	25.4	31.7	42.3	15.9	19.8	26.4	-3.2	-4.0	-5.3	-12.7	-15.9	-21.1
**Er^{3+}**	20.7	25.8	34.5	12.9	16.2	21.5	-2.6	-3.2	-4.3	-10.3	-12.9	-17.2
**Tm^{3+}**	12.9	16.1	21.5	8.0	10.1	13.4	-1.6	-2.0	-2.7	-6.4	-8.0	-10.7
**Yb^{3+}**	4.6	5.8	7.7	2.9	3.6	4.8	-0.6	-0.7	-1.0	-2.3	-2.9	-3.9

Table S4. Predicted transverse relaxation rates [Hz] arising from CSA×DSA cross correlation. B_0 = 9.4 T. τ_r = 0.25 ns. $\Delta_{\sigma_{CF3}^{CSA}} = 100\ ppm$,[68] T = 300 K.

ion	$\theta^{CSA,DSA}$ = 0°			$\theta^{CSA,DSA}$ = 30°			$\theta^{CSA,DSA}$ = 60°			$\theta^{CSA,DSA}$ = 90°		
	**10 Å**	**8 Å**	**6 Å**	**10 Å**	**8 Å**	**6 Å**	**10 Å**	**8 Å**	**6 Å**	**10 Å**	**8 Å**	**6 Å**
**Ce^{3+}**	2.0	2.5	3.3	1.2	1.6	2.1	-0.2	-0.3	-0.4	-1.0	-1.2	-1.7
**Pr^{3+}**	4.0	5.0	6.6	2.5	3.1	4.1	-0.5	-0.6	-0.8	-2.0	-2.5	-3.3
**Nd^{3+}**	4.0	5.1	6.7	2.5	3.2	4.2	-0.5	-0.6	-0.8	-2.0	-2.5	-3.4
**Sm^{3+}**	0.2	0.3	0.4	0.1	0.2	0.2	0.0	0.0	0.0	-0.1	-0.1	-0.2
**Eu^{3+}**	2.1	2.7	3.6	1.3	1.7	2.2	-0.3	-0.3	-0.4	-1.1	-1.3	-1.8
**Tb^{3+}**	29.4	36.7	48.9	18.4	22.9	30.6	-3.7	-4.6	-6.1	-14.7	-18.4	-24.5
**Dy^{3+}**	35.2	44.0	58.7	22.0	27.5	36.7	-4.4	-5.5	-7.3	-17.6	-22.0	-29.4
**Ho^{3+}**	35.0	43.7	58.3	21.9	27.3	36.4	-4.4	-5.5	-7.3	-17.5	-21.9	-29.1
**Er^{3+}**	28.5	35.6	47.5	17.8	22.3	29.7	-3.6	-4.5	-5.9	-14.3	-17.8	-23.8
**Tm^{3+}**	17.8	22.2	29.6	11.1	13.9	18.5	-2.2	-2.8	-3.7	-8.9	-11.1	-14.8
**Yb^{3+}**	6.4	8.0	10.7	4.0	5.0	6.7	-0.8	-1.0	-1.3	-3.2	-4.0	-5.3

Table S5. Predicted R_1 and R_2 arising from dipolar and Curie spin relaxations. The mathematical model encompasses Equations 1-6, 12 and 15. T_{1e} values calculated from Table 4. B_0 = 9.4 T. τ_R = 0.25 ns. T = 300 K.

Ion	10 Å		8 Å		6 Å	
	R_1	R_2	R_1	R_2	R_1	R_2
Ce^{3+}	1	2	2	3	4	5
Pr^{3+}	1	2	2	3	5	6
Nd^{3+}	1	2	2	4	9	11
Sm^{3+}	1	2	1	2	1	2
Eu^{3+}	1	2	1	3	3	5
Tb^{3+}	12	24	44	86	241	474
Dy^{3+}	15	30	54	108	297	596
Ho^{3+}	12	20	44	72	241	393
Er^{3+}	9	17	33	59	182	320
Tm^{3+}	6	11	19	35	100	188
Yb^{3+}	2	3	4	5	15	19

Table S6. Comparison between fluorine a_{HFC} obtained from DFT calculation and optimized fluorine a_{HFC} based on observed relaxation rates and BRW theoretical equations.

Complex symbol	a_{HFC} (DFT) [MHz]	a_{HFC} (fitted) [MHz]
**MnL[11]**	1.33	0
**CuL[11]**	0.03	0
**CoL[11]**	0.88	0.19
**NiL[11]**	0.81	0
**MnL[12]**	0.009	0.006
**CuL[12]**	0.004	0.008
**[NiL]**[45]	0.18	0.1
**[NiL^1]**[44]	0.35	0
**[NiL^2]**[44]	0.25	0
**trans-[Co^{II}(Hte2f2p)]$^-$** [26]	0.28	0.43
**trans-[Co^{II}(te2f2a)]**[26]	0.28	0
**[FC-Ni^{2+}]** [25]	0.0009	0.22
**[FC-Cu^{2+}]** [25]	0.0003	0.14
**[FC-Fe^{3+}]** [25]	0.0003	0.01

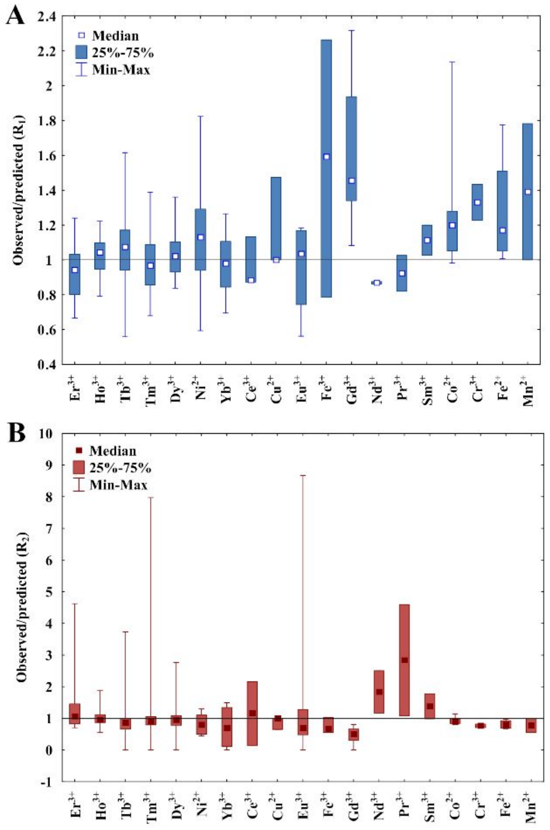

Figure S3. Deviations between the calculated and experimental relaxation times for all investigated ions expressed as the observed/calculated ratio.

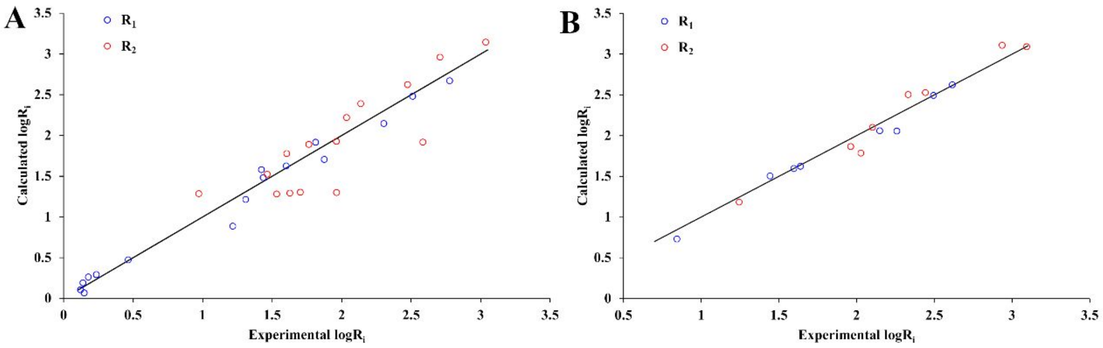

Figure S4. Observed and predicted relaxation times of **A** – ML^{11}. **B** – ML^{12} complexes.

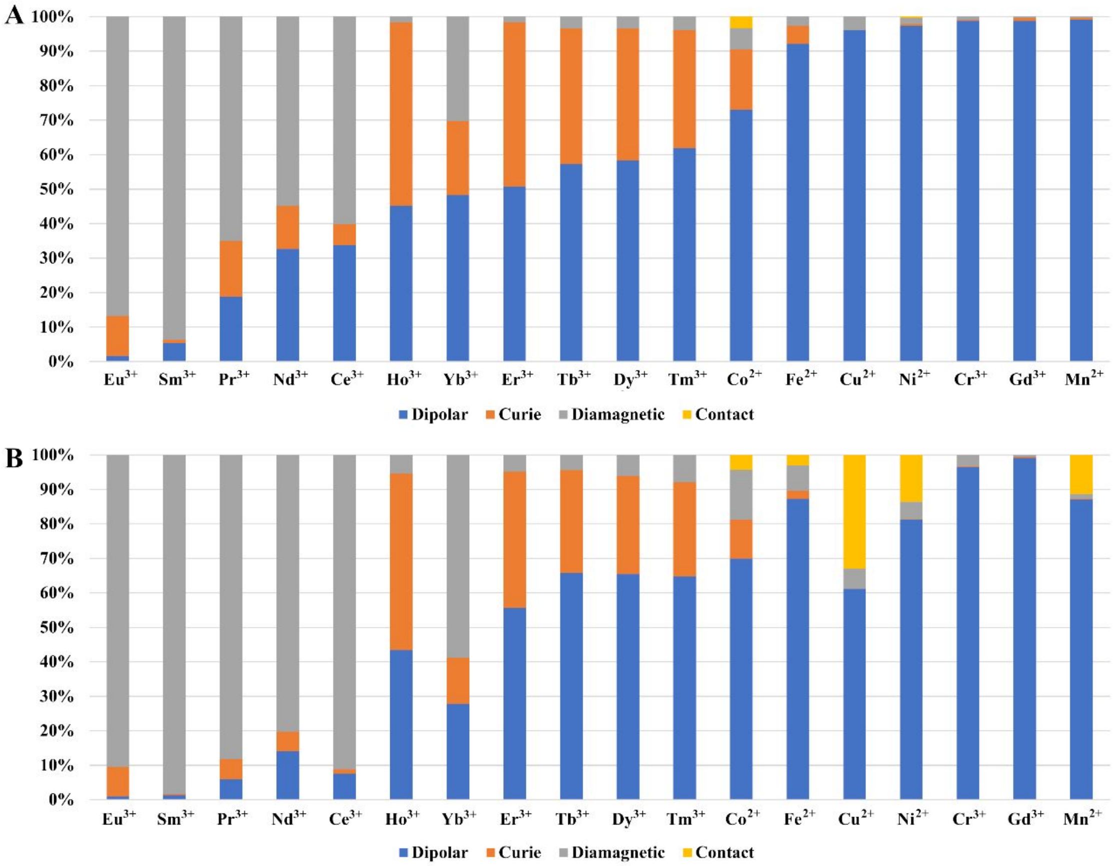

Figure S5. Average contribution of the main relaxation mechanisms for investigated complexes (Figure 1) of paramagnetic ions. **A**- R_1. **B**- R_2. The data were obtained by averaging the contribution ~~for~~ of each mechanism for each investigated complex.

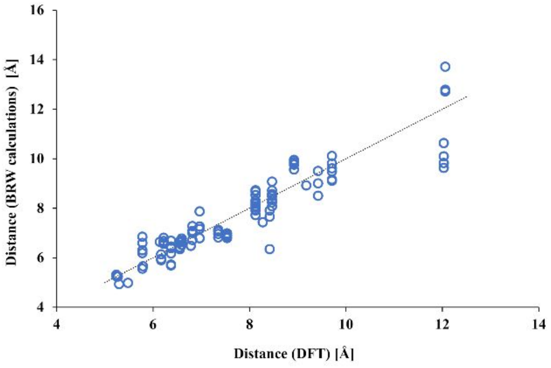

Figure S6. Comparison between DFT distances (Gd^{3+} complexes) and optimized distances calculated based on BRW equations for each individual complex.

Table S7. Summary of literature data used in the calculations of the model. $R_{i(ref)}$ indicate relaxation rates of diamagnetic references. $R_{i(exp)}$ indicate relaxation rates from original publications.

Designation in the original publication	Ion	B_0 [T]	T [K]	$R_{1(ref)}$	$R_{2(ref)}$	$R_{1(exp)}$	$R_{2(exp)}$	Reference
**7**	Dy^{3+}	11.7	310	1.75	1.19	4.8	13	46
**7**	Tb^{3+}	11.7	310	1.75	2.00	2.1	5.4	46
**7**	Eu^{3+}	11.7	310	1.27	1.19	0.72	0.8	46
**7**	Gd^{3+}	11.7	310	0.68	2.00	75	116	46
**[L^3]**	Dy^{3+}	4.7	295	1.00	2.00	110	217	49
**[L^3]**	Dy^{3+}	9.4	295	1.00	2.00	186	440	49
**[L^3-chitosan]**	Dy^{3+}	4.7	295	1.00	2.00	108	176	49
**[L^3-chitosan]**	Dy^{3+}	9.4	295	1.00	2.00	183	367	49
**[L^2]**	Ho^{3+}	4.7	295	1.00	2.00	59	173	49
**[L^2]**	Ho^{3+}	9.4	295	1.00	2.00	123	292	49
**[L^2-chitosan]**	Ho^{3+}	4.7	295	1.00	2.00	56	741	49
**[L^2-chitosan]**	Ho^{3+}	9.4	295	1.00	2.00	100	807	49
**[L^1]**	Er^{3+}	16.5	295	0.78	-	200	-	20
**[L^1]**	Er^{3+}	11.7	295	0.78	-	143	-	20
**[L^1]**	Er^{3+}	9.4	295	0.78	-	109	-	20
**[L^1]**	Er^{3+}	4.7	295	0.78	-	46	-	20
**[L^1]**	Ho^{3+}	16.5	295	0.78	-	341	-	20
**[L^1]**	Ho^{3+}	11.7	295	0.78	-	250	-	20
**[L^1]**	Ho^{3+}	9.4	295	0.78	-	192	-	20
**[L^1]**	Ho^{3+}	4.7	295	0.78	-	84	-	20
**[L^1]**	Tb^{3+}	16.5	295	0.78	-	282	-	20
**[L^1]**	Tb^{3+}	11.7	295	0.78	-	224	-	20
**[L^1]**	Tb^{3+}	9.4	295	0.78	-	185	-	20
**[L^1]**	Tb^{3+}	4.7	295	0.78	-	115	-	20
**[L^1]**	Tm^{3+}	4.7	295	0.78	-	33	-	20
**[L^1]**	Tm^{3+}	9.4	295	0.78	-	60	-	20
**[L^1]**	Tm^{3+}	11.7	295	0.78	-	76	-	20
**[L^1]**	Tm^{3+}	16.5	295	0.78	-	102	-	20
**[$H_3(L^2)$]**	Dy^{3+}	9.4	295	1.00	3.20	139	220	20
**[$H_3(L^2)$]**	Dy^{3+}	4.7	295	1.00	3.20	75	116	20
**[$H_3(L^2)$]**	Er^{3+}	9.4	295	1.00	3.20	65	157	20
**[$H_3(L^2)$]**	Er^{3+}	4.7	295	1.00	3.20	29	75	20
**[$H_3(L^2)$]**	Ho^{3+}	9.4	295	1.00	3.20	109	151	20
**[$H_3(L^2)$]**	Ho^{3+}	4.7	295	1.00	3.20	48	69	20
**[$H_3(L^2)$]**	Tb^{3+}	9.4	295	1.00	3.20	116	119	20
**[$H_3(L^2)$]**	Tb^{3+}	4.7	295	1.00	3.20	72	61	20
**[$H_3(L^2)$]**	Tm^{3+}	9.4	295	1.00	3.20	51	91	20
**[$H_3(L^2)$]**	Tm^{3+}	4.7	295	1.00	3.20	26	79	20
**[L^{6a}]_1**	Er^{3+}	16.5	295	1	-	313	-	20

Designation in the original publication	Ion	B_0 [T]	T [K]	$R_{1(ref)}$	$R_{2(ref)}$	$R_{1(exp)}$	$R_{2(exp)}$	Reference
**[L^6a]_1**	Er^{3+}	4.7	295	1	-	59	-	[20]
**[L^6a]_1**	Er^{3+}	11.7	295	1	-	209	-	[20]
**[L^6a]_1**	Er^{3+}	9.4	295	1	-	152	-	[20]
**[L^6a]_1**	Ho^{3+}	11.7	295	1	-	333	-	[20]
**[L^6a]_1**	Ho^{3+}	4.7	295	1	-	91	-	[20]
**[L^6a]_1**	Ho^{3+}	9.4	295	1	-	233	-	[20]
**[L^6a]_1**	Ho^{3+}	16.5	295	1	-	588	-	[20]
**[L^6a]_1**	Tb^{3+}	4.7	295	1	-	149	-	[20]
**[L^6a]_1**	Tb^{3+}	11.7	295	1	-	323	-	[20]
**[L^6a]_1**	Tb^{3+}	9.4	295	1	-	250	-	[20]
**[L^6a]_1**	Tb^{3+}	16.5	295	1	-	565	-	[20]
**[L^6a]_1**	Tm^{3+}	4.7	295	1	-	53	-	[20]
**[L^6a]_1**	Tm^{3+}	9.4	295	1	-	100	-	[20]
**[L^6a]_1**	Tm^{3+}	11.7	295	1	-	135	-	[20]
**[L^6a]_1**	Tm^{3+}	16.5	295	1	-	294	-	[20]
**[L^6a]_2**	Er^{3+}	16.5	295	1	-	161	-	[20]
**[L^6a]_2**	Er^{3+}	4.7	295	1	-	45	-	[20]
**[L^6a]_2**	Er^{3+}	9.4	295	1	-	88	-	[20]
**[L^6a]_2**	Er^{3+}	11.7	295	1	-	110	-	[20]
**[L^6a]_2**	Ho^{3+}	11.7	295	1	-	172	-	[20]
**[L^6a]_2**	Ho^{3+}	9.4	295	1	-	137	-	[20]
**[L^6a]_2**	Ho^{3+}	4.7	295	1	-	71	-	[20]
**[L^6a]_2**	Ho^{3+}	16.5	295	1	-	313	-	[20]
**[L^6a]_2**	Tb^{3+}	11.7	295	1	-	179	-	[20]
**[L^6a]_2**	Tb^{3+}	9.4	295	1	-	147	-	[20]
**[L^6a]_2**	Tb^{3+}	4.7	295	1	-	100	-	[20]
**[L^6a]_2**	Tb^{3+}	16.5	295	1	-	192	-	[20]
**[L^6a]_2**	Tm^{3+}	4.7	295	1	-	37	-	[20]
**[L^6a]_2**	Tm^{3+}	9.4	295	1	-	56	-	[20]
**[L^6a]_2**	Tm^{3+}	11.7	295	1	-	68	-	[20]
**[L^6a]_2**	Tm^{3+}	16.5	295	1	-	152	-	[20]
**[L^7]**	Dy^{3+}	4.7	295	1.00	-	89	156	[20]
**[L^7]**	Dy^{3+}	9.4	295	1.00	-	162	355	[20]
**[L^7]**	Dy^{3+}	11.7	295	1.00	-	201	543	[20]
**[L^7]**	Dy^{3+}	16.5	295	1.00	-	286	740	[20]
**[L^7]**	Er^{3+}	4.7	295	1.00	-	34	120	[20]
**[L^7]**	Er^{3+}	9.4	295	1.00	-	75	198	[20]
**[L^7]**	Er^{3+}	11.7	295	1.00	-	102	251	[20]
**[L^7]**	Er^{3+}	16.5	295	1.00	-	144	317	[20]

Designation in the original publication	Ion	B_0 [T]	T [K]	$R_{1(ref)}$	$R_{2(ref)}$	$R_{1(exp)}$	$R_{2(exp)}$	Reference
**[L^7]**	Ho^{3+}	4.7	295	1.00	-	46	87	[20]
**[L^7]**	Ho^{3+}	9.4	295	1.00	-	124	192	[20]
**[L^7]**	Ho^{3+}	11.7	295	1.00	-	169	267	[20]
**[L^7]**	Ho^{3+}	16.5	295	1.00	-	239	441	[20]
**[L^7]**	Tb^{3+}	4.7	295	1.00	-	74	124	[20]
**[L^7]**	Tb^{3+}	9.4	295	1.00	-	133	206	[20]
**[L^7]**	Tb^{3+}	11.7	295	1.00	-	162	271	[20]
**[L^7]**	Tb^{3+}	16.5	295	1.00	-	211	407	[20]
**[L^7]**	Tm^{3+}	4.7	295	1.00	-	23	53	[20]
**[L^7]**	Tm^{3+}	9.4	295	1.00	-	47	89	[20]
**[L^7]**	Tm^{3+}	11.7	295	1.00	-	58	112	[20]
**[L^7]**	Tm^{3+}	16.5	295	1.00	-	74	168	[20]
**[L^{1a}]**	Dy^{3+}	4.7	295	1.00	22.22	92	96	[30]
**[L^{1a}]**	Dy^{3+}	9.4	295	1.00	22.22	158	213	[30]
**[L^{1a}]**	Er^{3+}	4.7	295	1.00	22.22	33	78	[30]
**[L^{1a}]**	Er^{3+}	9.4	295	1.00	22.22	81	164	[30]
**[L^{1a}]$^+$**	Ho^{3+}	4.7	295	1.00	22.22	47	72	[30]
**[L^{1a}]**	Ho^{3+}	9.4	295	1.00	22.22	91	159	[30]
**[L^{1a}]**	Tb^{3+}	4.7	295	1.00	22.22	100	41	[30]
**[L^{1a}]**	Tb^{3+}	9.4	295	1.00	22.22	94	132	[30]
**[L^{1a}]**	Tm^{3+}	4.7	295	1.00	22.22	24	84	[30]
**[L^{1a}]**	Tm^{3+}	9.4	295	1.00	22.22	56	102	[30]
**[L^{2b}]**	Dy^{3+}	9.4	298	1.20	15.00	185	251	[30]
**[L^{2b}]**	Dy^{3+}	7	298	1.20	12.00	144	191	[30]
**[L^{2b}]**	Dy^{3+}	4.7	298	1.20	10.00	104	135	[30]
**[L^{2b}]**	Er^{3+}	9.4	298	1.20	15.00	109	138	[30]
**[L^{2b}]**	Er^{3+}	7	298	1.20	12.00	91	102	[30]
**[L^{2b}]**	Er^{3+}	4.7	298	1.20	10.00	71	67	[30]
**[L^{2b}]**	Ho^{3+}	9.4	298	1.20	15.00	120	143	[30]
**[L^{2b}]**	Ho^{3+}	7	298	1.20	12.00	88	108	[30]
**[L^{2b}]**	Ho^{3+}	4.7	298	1.20	10.00	58	74	[30]
**[L^{2b}]**	Tb^{3+}	9.4	298	1.20	15.00	147	267	[30]
**[L^{2b}]**	Tb^{3+}	7	298	1.20	12.00	113	183	[30]
**[L^{2b}]**	Tb^{3+}	4.7	298	1.20	10.00	84	102	[30]
**[L^{2b}]**	Tm^{3+}	4.7	298	1.20	10.00	47	60	[30]
**[L^{2b}]**	Tm^{3+}	9.4	298	1.20	15.00	63	84	[30]
**[L^{2b}]**	Tm^{3+}	7	298	1.20	12.00	56	72	[30]
**DOTAm-F_{12}**	Dy^{3+}	7	295	1.75	2.50	169	455	[27]
**DOTAm-F_{12}**	Er^{3+}	7	295	1.75	2.50	71	114	[27]

Designation in the original publication	Ion	B_0 [T]	T [K]	$R_{1(ref)}$	$R_{2(ref)}$	$R_{1(exp)}$	$R_{2(exp)}$	Reference
**DOTAm-F_{12}**	Ho^{3+}	7	295	1.75	2.50	132	185	27
**DOTAm-F_{12}**	Tm^{3+}	7	295	1.75	2.50	38	63	27
**DOTAm-F_{12}**	Tb^{3+}	7	295	1.75	2.50	159	769	27
**DOTAm-F_{12}**	Eu^{3+}	7	295	1.75	2.50	2.8	24	27
**[DOTAm-F_{12}**	Fe^{2+}	7	295	1.75	2.50	175	179	27
**DOTAm-F_{12}**	Gd^{3+}	7	295	1.75	2.50	83	7143	27
**DOTAm-F_{12}**	Yb^{3+}	7	295	1.75	2.50	7.1	18	27
**[HL^1]**	Ni^{2+}	7	298	1.00	2.00	99	125	44
**[HL^2]**	Ni^{2+}	7	298	1.00	2.00	34	41	44
**te2f**	Ni^{2+}	0.94	298	2.00	-	36	-	55
**te2f**	Ni^{2+}	7	298	1.22	-	357	-	55
**te2f**	Ni^{2+}	9.4	298	2.67	-	556	-	55
**FC**	Ni^{2+}	11.7	298	0.66	1.25	18	200	25
**FC**	Dy^{3+}	11.7	298	0.66	1.25	13	20	25
**FC**	Er^{3+}	11.7	298	0.66	1.25	8.0	13	25
**FC**	Ho^{3+}	11.7	298	0.66	1.25	9.1	17	25
**FC**	Tb^{3+}	11.7	298	0.66	1.25	12	20	25
**FC**	Ce^{3+}	11.7	298	0.66	1.25	0.80	2	25
**FC**	Cu^{2+}	11.7	298	0.66	1.25	11	1000	25
**FC**	Eu^{3+}	11.7	298	0.66	1.25	0.83	2	25
**FC**	Fe^{3+}	11.7	298	0.66	1.25	125	200	25
**FC**	Gd^{3+}	11.7	298	0.66	1.25	256	200	25
**FC**	Nd^{3+}	11.7	298	0.66	1.25	1.0	2.2	25
**FC**	Pr^{3+}	11.7	298	0.66	1.25	0.95	1.7	25
**FC**	Sm^{3+}	11.7	298	0.66	1.25	0.71	1.3	25
**FC**	Yb^{3+}	11.7	298	0.66	1.25	1.39	2.9	25
**[L^c]**	Dy^{3+}	9.4	298	0.80	2.10	-	15	48
**[L^c]**	Dy^{3+}	7	298	0.80	2.10	-	63	48
**[L^c]**	Tb^{3+}	9.4	298	0.80	2.10	-	12	48
**[L^c]**	Tb^{3+}	7	298	0.80	2.10	-	84	48
**[L^c]**	Tm^{3+}	9.4	298	0.80	2.10	-	5.9	48
**[L^c]**	Tm^{3+}	7	298	0.80	2.10	-	83	48
**[L^c]**	Eu^{3+}	9.4	298	0.80	2.10	-	1.1	48
**[L^c]**	Eu^{3+}	7	298	0.80	2.10	-	2.8	48
**[L^c]**	Gd^{3+}	9.4	298	0.80	2.10	-	300	48
**[L^c]**	Gd^{3+}	7	298	0.80	2.10	-	463	48
**[L^c]**	Dy^{3+}	5.9	298	0.80	2.10	10	-	48
**[L^c]**	Dy^{3+}	7	298	0.80	2.10	12	-	48
**[L^c]**	Tb^{3+}	5.9	298	0.80	2.10	8.5	-	48

Designation in the original publication	Ion	B_0 [T]	T [K]	$R_{1(ref)}$	$R_{2(ref)}$	$R_{1(exp)}$	$R_{2(exp)}$	Reference
**[L^c]**	Tb^{3+}	7	298	0.80	2.10	9.8	-	48
**[L^c]**	Tm^{3+}	5.9	298	0.80	2.10	4.5	-	48
**[L^c]**	Tm^{3+}	7	298	0.80	2.10	4.9	-	48
**[L^c]**	Eu^{3+}	5.9	298	0.80	2.10	1.0	-	48
**[L^c]**	Eu^{3+}	7	298	0.80	2.10	1.0	-	48
**[L^c]**	Gd^{3+}	5.9	298	0.80	2.10	344	-	48
**[L^c]**	Gd^{3+}	7	298	0.80	2.10	330	-	48
**DOTP-tfe**	Dy^{3+}	7	298	1.20	53.80	145	256	50
**DOTP-tfe**	Ho^{3+}	7	298	1.20	53.80	102	122	50
**DOTP-tfe**	Tm^{3+}	7	298	1.20	53.80	154	204	50
**DOTP-tfe**	Ce^{3+}	7	298	1.20	53.80	3.5	8.1	50
**DOTP-tfe**	Yb^{3+}	7	298	1.20	53.80	13	14	50
**[L^a]**	Tb^{3+}	7	298	1.00	2.00	32	48	52
**[L^a]**	Eu^{3+}	7	298	1.38	2.00	1.40	18	52
**[L^a]**	Gd^{3+}	7	298	1.38	2.00	182	385	52
**[H_3L^1] (meta)**	Dy^{3+}	4.7	298	0.90	-	12	-	19
**[H_3L^1] (meta)**	Ho^{3+}	4.7	298	0.90	-	8.1	-	19
**[H_3L^1] (meta)**	Tb^{3+}	4.7	298	0.90	-	11	-	19
**[H_3L^1] (meta)**	Tm^{3+}	4.7	298	0.90	-	5.6	-	19
**[H_3L^1] (meta)**	Yb^{3+}	4.7	298	0.90	-	1.7	-	19
**[H_3L^1] (orto)**	Dy^{3+}	4.7	298	0.90	-	77	-	19
**[H_3L^1] (orto)**	Ho^{3+}	4.7	298	0.90	-	49	-	19
**[H_3L^1] (orto)**	Tb^{3+}	4.7	298	0.90	-	90	-	19
**[H_3L^1] (orto)**	Tm^{3+}	4.7	298	0.90	-	16	-	19
**[H_3L^1] (orto)**	Yb^{3+}	4.7	298	0.90	-	4.6	-	19
**[L^b]**	Ni^{2+}	1.4	310	1.00	1.19	16	18	45
**[L^b]**	Ni^{2+}	9.4	296	1.00	1.19	69	82	45
**[L^b]**	Co^{2+}	1.4	310	0.64	1.19	22	24	45
**[L^b]**	Co^{2+}	9.4	296	0.64	1.19	24	34	45
**[L^b]**	Fe^{2+}	1.4	310	0.64	1.19	18	20	45
**[L^b]**	Fe^{2+}	9.4	296	0.64	1.19	26	56	45
**H_4te2f2p**	Co^{2+}	7	300	1.00	1.00	81	104	26
**H_4te2f2p**	Co^{2+}	9.4	300	1.00	1.00	84	112	26
**H_2te2f2a**	Co^{2+}	7	300	1.00	1.00	62	91	26
**H_2te2f2a**	Co^{2+}	9.4	300	1.00	1.00	64	96	26
**H_2te2f2a**	Ni^{2+}	0.94	298	1.23	-	83	-	55
**H_2te2f2a**	Ni^{2+}	7	298	1.61	-	500	-	55
**H_2te2f2a**	Ni^{2+}	9.4	298	1.85	-	667	-	55
**te2f2ae**	Ni^{2+}	0.94	298	1.37	-	167	-	55

Designation in the original publication	Ion	B_0 [T]	T [K]	$R_{1(ref)}$	$R_{2(ref)}$	$R_{1(exp)}$	$R_{2(exp)}$	Reference
**te2f2ae**	Ni^{2+}	7	298	1.41	-	588	-	[55]
**te2f2ae**	Ni^{2+}	9.4	298	1.97	-	833	-	[55]
**H_4te2f2p**	Ni^{2+}	0.94	298	0.94	-	200	-	[55]
**H_4te2f2p**	Ni^{2+}	7	298	0.91	-	581	-	[55]
**H_4te2f2p**	Ni^{2+}	9.4	298	1.40	-	588	-	[55]
**te2f2py**	Ni^{2+}	0.94	298	1.41	-	56	-	[55]
**te2f2py**	Ni^{2+}	7	298	1.89	-	556	-	[55]
**te2f2py**	Ni^{2+}	9.4	298	2.19	-	714	-	[55]

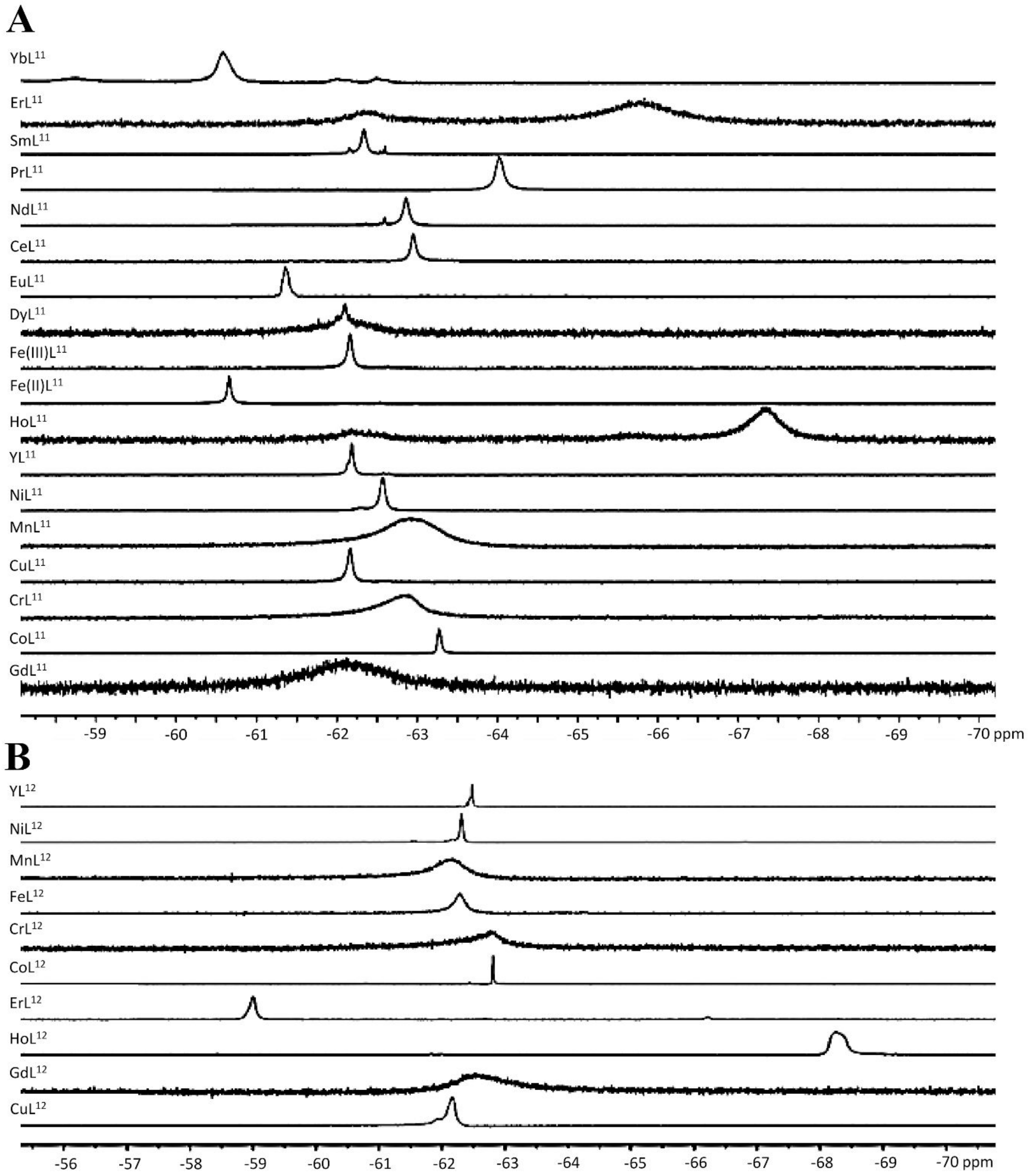

Figure S7. ^{19}F NMR spectra. **A**- L^{11} **B**- L^{12} complexes.

Optimized cartesian coordinates for ground-state of Gd^{3+} complexes of L^{11} and L^{12} ligands:

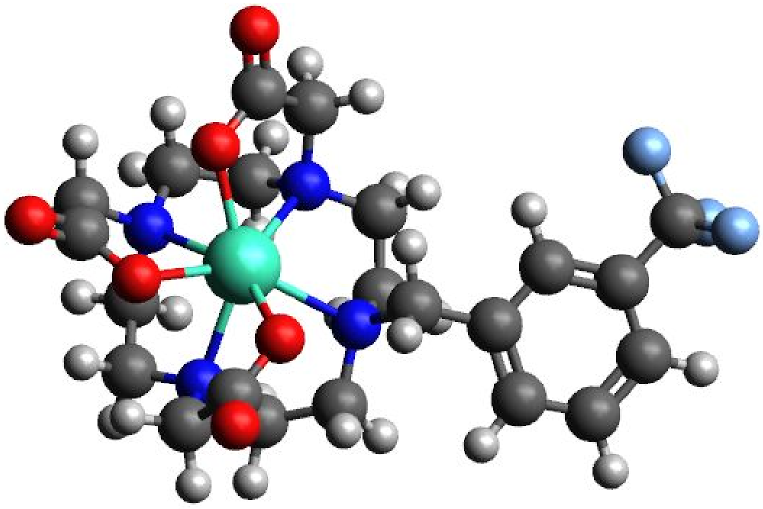

GdL^{11}

N	-3.101	2.909	0.199
C	-4.062	3.066	1.335
C	-4.697	1.705	1.793
H	-4.916	3.724	1.085
H	-3.548	3.556	2.192
N	-5.457	0.940	0.738
H	-3.847	1.106	2.162
H	-5.339	1.876	2.686
C	-5.654	-0.474	1.106
C	-4.383	-1.277	0.856
H	-6.455	-0.941	0.499
H	-5.982	-0.565	2.166
N	-3.859	-1.144	-0.538
H	-3.622	-0.919	1.572
H	-4.538	-2.341	1.148
C	-2.500	-1.700	-0.646
C	-1.506	-0.632	-0.286
H	-2.267	-2.027	-1.676
H	-2.372	-2.605	-0.010
N	-1.710	0.591	-1.098
H	-0.467	-1.019	-0.358
H	-1.646	-0.393	0.784
C	-0.969	1.782	-0.604
C	-1.736	2.448	0.573
H	-0.801	2.546	-1.389
H	0.034	1.483	-0.231
H	-1.808	1.705	1.388
H	-1.141	3.288	0.994
C	-3.053	4.178	-0.605
C	-2.944	3.880	-2.077
H	-2.206	4.819	-0.276

H	-3.966	4.801	-0.498
O	-2.963	2.597	-2.461
O	-2.774	4.771	-2.893
C	-1.335	0.312	-2.533
C	-4.794	-1.953	-1.394
C	-5.854	-1.128	-2.018
H	-5.239	-2.823	-0.857
H	-4.272	-2.391	-2.273
O	-6.731	-1.644	-2.693
O	-5.862	0.182	-1.800
C	-6.765	1.632	0.472
C	-6.669	2.678	-0.614
H	-7.193	2.066	1.403
H	-7.523	0.913	0.091
O	-5.474	2.926	-1.160
O	-7.637	3.358	-0.914
C	0.005	-0.380	-2.764
H	-1.297	1.227	-3.152
H	-2.150	-0.288	-2.986
C	0.033	-1.674	-3.320
C	1.253	-2.336	-3.551
C	2.436	-0.399	-2.690
C	1.223	0.253	-2.463
C	2.453	-1.687	-3.227
C	1.302	-3.725	-4.139
F	1.931	-4.579	-3.253
F	0.035	-4.219	-4.401
F	2.016	-3.697	-5.324
H	-0.898	-2.164	-3.571
H	1.250	1.255	-2.065
H	3.404	-2.177	-3.398
H	3.369	0.097	-2.454
Gd	-4.040	1.238	-1.078

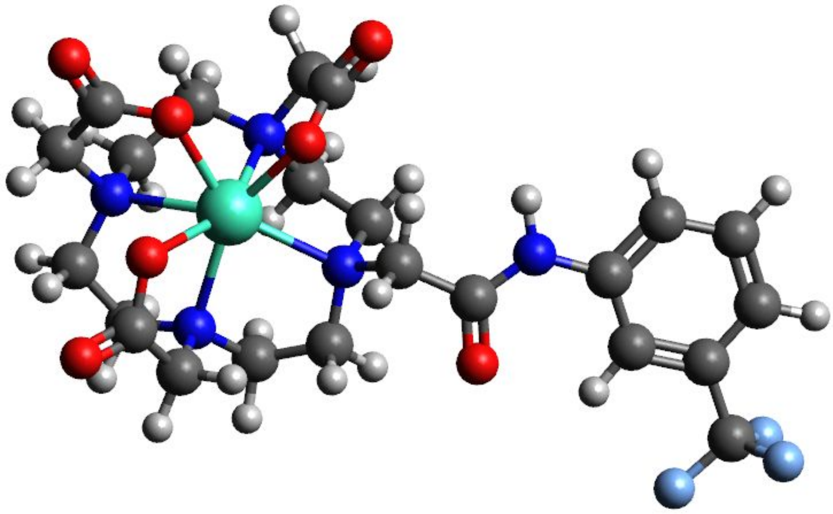

GdL[12]

N	-0.233	0.303	-0.047
C	0.117	1.099	-1.271
C	1.248	2.121	-1.048
H	0.476	0.465	-2.095
H	-0.780	1.607	-1.690
N	2.523	1.515	-0.569
H	0.843	2.829	-0.293
H	1.433	2.703	-1.978
C	3.296	0.775	-1.585
C	4.467	-0.010	-0.934
H	3.696	1.476	-2.351
H	2.640	0.078	-2.122
N	4.014	-0.938	0.144
H	5.170	0.745	-0.525
H	5.053	-0.544	-1.714
C	3.789	-2.330	-0.342
C	2.438	-2.548	-0.989
H	3.849	-3.047	0.501
H	4.580	-2.651	-1.054
N	1.317	-2.087	-0.137
H	2.310	-3.619	-1.265
H	2.481	-2.000	-1.947
C	0.121	-1.958	-1.011
C	-0.885	-0.966	-0.456
H	-0.357	-2.946	-1.198
H	0.396	-1.598	-2.020
H	-1.377	-1.459	0.401
H	-1.706	-0.787	-1.188
C	3.356	2.564	0.121
C	3.729	2.234	1.567
H	2.842	3.550	0.162
H	4.283	2.778	-0.453

O	3.267	1.131	2.198
O	4.443	3.008	2.182
C	-1.183	1.048	0.831
C	-0.465	1.775	1.898
H	-1.891	0.384	1.376
H	-1.811	1.753	0.242
O	0.839	1.554	2.070
O	-1.062	2.528	2.651
C	1.076	-3.004	1.008
C	0.213	-2.342	2.041
H	2.023	-3.173	1.549
H	0.687	-3.999	0.695
O	0.432	-1.042	2.312
O	-0.728	-2.930	2.551
C	5.031	-0.995	1.242
C	6.399	-1.527	0.820
H	4.647	-1.602	2.092
H	5.195	0.015	1.648
O	6.493	-2.691	0.472
N	7.494	-0.705	0.865
C	8.856	-1.020	0.519
C	9.813	0.005	0.649
C	11.154	-0.213	0.335
C	11.573	-1.461	-0.116
C	10.654	-2.508	-0.260
C	9.298	-2.286	0.057
H	9.518	0.987	0.997
H	12.619	-1.612	-0.355
H	11.871	0.591	0.443
H	8.626	-3.112	-0.067
C	11.147	-3.848	-0.753
F	10.126	-4.777	-0.843
F	12.106	-4.333	0.118
F	11.713	-3.696	-2.006
H	7.327	0.263	1.180
Gd	1.916	-0.060	0.966
